# Facilitators and barriers to physical activity in people with chronic low back pain: A qualitative study

**DOI:** 10.1371/journal.pone.0179826

**Published:** 2017-07-25

**Authors:** Laura Boutevillain, Arnaud Dupeyron, Caroline Rouch, Emilie Richard, Emmanuel Coudeyre

**Affiliations:** 1 Service de Médecine Physique et de Réadaptation, Centre Hospitalier Universitaire, Clermont-Ferrand, France; 2 INRA, Université Clermont Auvergne, Clermont-Ferrand, France; 3 Service de Médecine Physique et de Réadaptation, Faculté de Médecine Montpellier-Nîmes, Centre Hospitalier Universitaire Carémeau, Nîmes, France; 4 Euromov, Université de Montpellier, Montpellier, France; University of Edinburgh, UNITED KINGDOM

## Abstract

**Background:**

For medical teams, one of the main objectives of rehabilitation for people with chronic low back pain is adherence to physical activity (PA).

**Objective:**

The objective of this study was to identify PA barriers and facilitators in this population.

**Methods:**

This qualitative study included 4 discussion groups and 16 semi-structured interviews conducted among people with non-specific chronic low back pain who were involved in a specific rehabilitation program or seen in primary care settings.

**Results:**

Three main themes were identified: physical factors, psychological factors and socio-environmental factors. The main barrier to PA practice is pain. Psychological barriers were associated with the difficulty in integrating PA in the person’s daily life. Environmental barriers were dominated by lack of time. Facilitators identified associated the supervised nature of the physical activity (supervision by professionals) and group practice, which improved people’s adherence.

**Conclusion:**

The results of this study will allow teams to target relevant educational objectives for these people and develop dedicated self-management programs.

## Introduction

Physical activity is an integrant part of the care management of several chronic diseases (e.g. multiple sclerosis, cancer, coronary diseases, obesity) [1], and constitutes one of the objectives of multidisciplinary programs for non-specific chronic low back pain (CLBP) [2,3]. PA is described by the World Health Organization (WHO) as “any bodily movement produced by skeletal muscles that requires energy expenditure” [4]. This includes sports activities but also other daily activities such as housework or gardening.

The National Institute for Care and Excellence (NICE) and the European working group on CLBP integrated physical activity into their guidelines [5,6]. Physiotherapy management usually includes therapeutic education for patients on exercises they can perform alone at home in order to transition towards physical activities adapted to each patient’s condition in order to decrease the functional impact of CLPB. PA’s effectiveness in the care management of people with CLBP depends, among other things, on a person’s adherence to the home exercise program and sustainable efforts on the long term, as well as resuming additional physical activities [7].

The rate of non-adherence to home exercise programs in this population reaches 50% and sometimes even more [8], yet these data are hard to quantify. In 1993, Sluijs and colleagues evaluated the adherence of patients to a physical activity program and highlighted certain main factors for non-adherence (barriers perceived by people with CLBP, disease-related level of disability, lack of PA positive effect) [9].

As a matter of fact, people with CLBP often have inaccurate beliefs regarding their condition and its treatment [10], which in turn influences their adherence to exercise programs [11], especially regularity in practicing a physical activity.

Qualitative studies have been published analyzing the satisfaction and experience with CLBP [12], type of exercise [13], or even factors influencing the adherence to exercise programs [8,11] in people with low back pain. However, these studies did not solely focus on people with CLBP, but also included persons with neck pain [8,11]. In 2014, a review of the literature on qualitative studies focusing on PA-related beliefs in people with non-specific CLBP highlighted several barriers and facilitators to PA practice in this population [14], but did not specifically explore barriers and facilitators to PA; therefore, there was a need to explore it in more details in this study.

The objective of our study was specifically identifying barriers and facilitators to PA practice in people with non-specific CLBP, both in primary and secondary care, in order to validate and enrich the array of barriers and facilitators previously identified in previous studies.

## Materials and methods

### Type of study

This is a cross-sectional qualitative study with two data collection methods, based on semi-structured individual interviews and focus groups (semi-structured group interviews) conducted in Clermont-Ferrand, France from January 2012 to April 2014 (individual interviews were conducted from January to April 2012 and focus groups were conducted from February to April 2014).

The objective of the individual interviews was to identify barriers to physical activity in people with CLBP. Focus groups were conducted later to corroborate the barriers unveiled in the individual interviews and identify PA facilitators. The use of the focus group method enabled, via group interaction, to explore and better refine the opinion of each participant [15].

This type of qualitative study was chosen according to the principle of the grounded theory [16], to study and better understand how beliefs and thoughts in people with CLBP influence their behaviors towards PA, in order to formulate new hypotheses regarding predictive factors of regular physical activity in people with CLBP, and improve the care management in this population.

This study was conducted and reported according to the Consolidated Criteria for Reporting Qualitative Research (COREQ) [17].

### People with CLBP

#### Recruitment

For individual interviews, people with CLBP were recruited via three primary care physicians (independent from researchers). They had to identify in their daily practice people with CLBP who met the study’s inclusion criteria (purposive sampling method). People with CLBP were then contacted by phone to schedule a face-to-face interview. Other people with CLBP were recruited at the Physical Medicine and Rehabilitation (PM&R) Department of the Clermont-Ferrand University Hospital, directly by the study investigator during the course of their care management in the service. People with CLBP were then contacted by phone to set up an interview.

For focus groups, recruitment took place within the Physical Medicine and Rehabilitation (PM&R) Department of Clermont-Ferrand, University Hospital, France via the functional spine rehabilitation program, also using the purposive sampling method.

Inclusion criteria for this study were: age > 18 with non-specific CLBP, as defined by pain and discomfort, localized below the costal margin and above the inferior gluteal folds, with or without referred leg pain, persisting for at least 12 weeks and not attributable to a recognizable, known specific pathology (e.g. infection, tumor, osteoporosis, fracture, structural deformity, inflammatory disorder (e.g. ankylosing spondylitis), radicular syndrome or cauda equina syndrome) [6]. Exclusion criteria consisted in: presence of physical or mental impairment preventing the person’s participation in a focus group/individual interview or filling-out written questionnaires, not speaking French and specific low back pain.

#### Data collection on people with CLBP

Nominative data were collected for each person with CLBP. They also filled out evaluation scales on low back pain: The Back Belief Questionnaire (BBQ; assessing patients’ knowledge) [18], the Quebec Back Pain Disability Scale [19] (evaluating the impact of low back pain on daily life), Fear Avoidance Beliefs Questionnaire [20] (FABQ that evaluates fears and beliefs) and the Numeric Pain Rating Scale.

### Interview guide

Two interview guides were developed in order to structure the individual interviews and focus groups. These guides were designed based on data from the literature, rehabilitation and occupational therapy experience and advice of low back pain experts (EC, AD). They included several main topics: low back pain, physical activity, barriers and facilitators (only for the focus group interview guide) to physical activity. These guides were not tested prior to the beginning of the study.

### Study protocol

The purpose of individual interviews was to allow people with CLBP to speak freely about factors limiting their physical activity practice. Four interviews took place in a home setting, four others in a primary care setting, and the last nine interviews in a PM&R department setting (and in that latter case, the location of the interview was chosen by the person with CLBP). These interviews were conducted by a primary care (PC) resident. The person in charge of individual interviews had to lead the interview and encourage participants to express themselves freely.

Focus groups were organized in a room within the Clermont-Ferrand university hospital PM&R department. Each of the focus group started first with a review of the study objectives, and then people with CLBP were invited to share their experiences with low back pain and PA. A moderator and an observer were present in each focus group (PM&R resident (LB) and adapted physical activity trainer (CR) working within the PM&R department).

The interviewers (for both the focus groups and individual interviews) did not have a relationship with people with CLBP prior to recruitment and were not involved in their care management.

The role of the focus group moderator was to manage the group dynamics and goals. The focus group observer was responsible for the recording material and taking notes. Interview guides were used as the main thread for conducting individual and focus group interviews, recorded with a dictation recorder and transcribed in their entirety (verbatim). The study ended when data saturation was reached (“point at which no new information or themes were observed for the data” [21]).

The three persons (ER, LB and CR) who conducted the individual and focus group interviews received a specific training on qualitative research prior to the study.

#### Data analysis

Data were analyzed manually by coding them according to the data triangulation method: the first step consisted in reading the verbatim transcriptions of the different interviews/focus groups, and file each sentence/idea into different categories. The second step consisted in grouping the categories into themes. The third step was dedicated to assembling these themes to form more general concepts [22]. These themes and categories were inductively generated during the research, according to the grounded theory, method so they can name most accurately what the data were suggesting [16]. Data were analyzed progressively alongside the interviews and focus groups (i.e. data collection and analysis were performed simultaneously), with an adjustment of the themes and categories according to the data collected, according to the constant comparative analysis principle. The individual interview guide was refined during the study, in light of the first emerging results, and data stemming from the individual interviews helped design the focus group- interview guide. The literature research was conducted at the end of the study, in order for the data analysis to remain neutral and not influenced by themes existing in other studies.

Two persons [CR, LB] performed independently the data analysis and their conclusions were merged. When they disagreed on a result, the project manager [EC] made the final call.

### Ethics considerations

Oral and written information on the study was given out to each people with CLBP. An informed and written consent form was collected for each participant not opposed to the study. People with CLBP were warned that they were free to accept or refuse to participate in the study and that it would in no way affect their quality of care. Data were anonymized by a third person who deleted the first and last names of participants and replaced them by a number.

Data were then stored on the server of the Clermont-Ferrand University Hospital, authorized to store healthcare-related data. The study received the approval from the Comité d’Ethique des Centres d’Investigation Clinique de l’inter-région Rhône-Alpes-Auvergne, centre de Grenoble, on March 22, 2012 (N° IRB 5044).

The study was reported on the Clinical Trials website on June 5, 2015, under the number NCT02466360.

## Results and analysis

**Fig 1:**
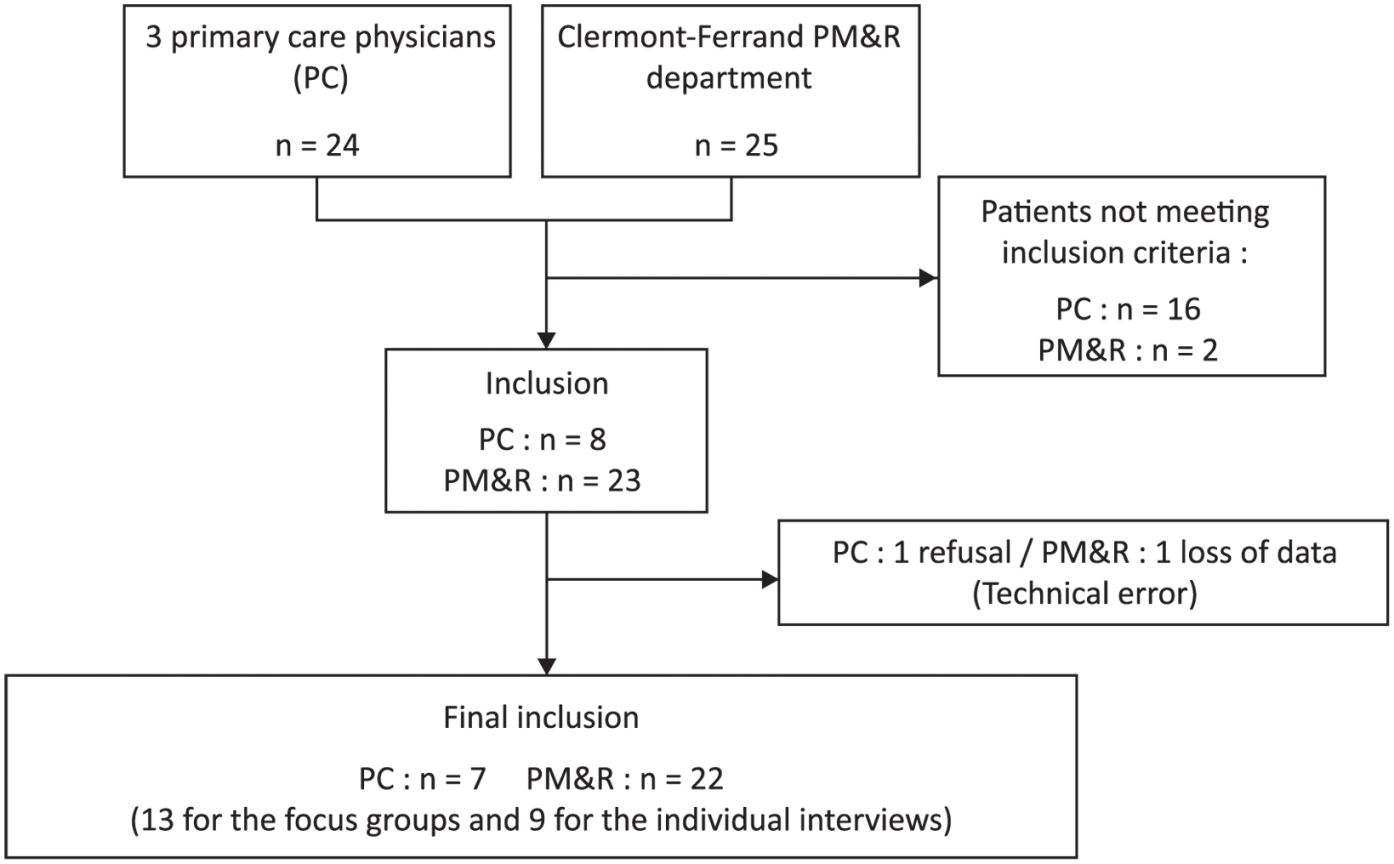
Flow diagram.

Twenty-nine people with CLBP participated in the study (Fig 1): 16 for the individual interviews and 13 for the focus groups (4 focus groups: 4 persons for focus group 1, then 3 persons for the other 3 focus groups). The mean duration of individual interviews was 20 minutes (7 15-minute interviews, 2 interviews that lasted about 20 min and 6 interviews that lasted longer than 20 min). The mean duration of the focus groups was 27 minutes (30 min, 22 min, 39 min and 18 min). Characteristics of the study population are summed up in Table 1.

**Table 1:** Characteristics of the study population.

Variables	Number of people with CLBP (n = 29)
Men / Women	19 / 10
20–30 years / 31–40 years / 41–55 years	3 / 10 / 16
Time since the onset of low back pain/year	
< 5 years / 6–10 years / >10 years	13 / 8 / 8
Occupational type	
Sedentary / physical / combination of both	7 / 19 / 3
Physical activity practice	
Yes / no	14 / 15
Physical FABQ (/24)	
< 14 / >14	9 / 20
Occupation FABQ (/42)	
< 34 / > 34	22 / 7
QUEBEC (/100)	
<40 / > 40	15 / 14
BBQ (/45)	
<23 / > 23	6 / 23
Numeric scale (/10)	
<5 / >5	9 / 20

Three main categories of barriers and facilitators to PA practice were identified: physical, psychological and environmental/professional. Sub categories were highlighted within each of these three main themes.

### Barriers

#### Physical barriers

Pain: Pain appears to be the main barrier to physical activity. It was reported by most study participants. Pain intensity can sometimes be quite important: *“any minimal physical activity*, *standing still in one spot*, *is torture”* (line 1683); *“when I am in pain*, *I do not do any physical activity”* (line 499); *“at times*, *just standing at the kitchen counter and peeling 3 vegetables is enough to trigger the pain*, *a pain so unberable I could cry”* (line 1293). Pain does seem to impact not only physical activity but activities of daily living: *“simple daily chores*, *washing dishes or shopping in general is torture in fact”* (line 1680). Pain also has a psychological impact, bearing a negative influence on physical activity leading to a feeling of disability, social isolation, and loss of self-confidence: *“after a while it affects our well-being…everything seems quite negative in fact”* (line 1669); *“if my back hurts*, *I don’t do any activity that’s for sure*, *I am not going to the garden and do some digging*, *that is out of the question*! *I have two children*, *if I am in pain and they want to play*, *my back hurts and I can’t play with them*. *My back hurts I can’t do it*. *It’s not that I don’t want to it is just that I cannot*. *I am unable to”* (line 29).

Comorbidities: For some people with CLBP, other back pain-related pathologies are responsible for the decrease in physical activity, especially osteoarthritis *“I am unable to do certain physical activities*: *running in particular*. *I have knee osteoarthritis as well*, *so I can’t run”* (line 1043). Being overweight and having headaches also represent barriers for certain people with CLBP: *« What really helped before was running*, *now my weight gain really prevents me from enjoying it as I used to”* (line 451). The presence of other painful spots also affect PA practice: *“When I go walking*, *I realize that the pain is in my hips and in the sole of my feet*.*”* (line 465).

#### Psychological barriers

Lack of motivation and will to exercise: A large proportion of people with CLBP brought up their lack of motivation and will to exercise *“I don’t have any desire to exercise*. *A lack of motivation*, *even apprehension”* (line 390); *“there needs to be this spark to get motivated*, *and I just don’t have it”* (line 1335). This lack of motivation is sometimes pain-related: *“motivation sometimes is…When one hurts the motivation goes down”* (line 1791); or fatigue: *“some days we are motivated*, *but other days the fatigue takes over…”* (line 1883).

Kinesiophobia and anticipatory anxiety: Another consequence of pain is kinesiophobia, with or without anticipatory anxiety: *“when I’m in pain*, *I don’t do much*, *I stay on the couch*! *Rest*! *It’s a fold-up couch*! *I move around a little but I am careful about the movements I make*” (line 1420); *“I need to be aware for any movement I make” (*line 1581). People with CLBP interviewed brought up the fear of aggravating the pain: *“The pain and sometimes even if it doesn’t hurt right away*, *knowing we will be in pain later*, *in 2 hours or right after*, *and 3 hours later the pain will be intense”* (line 2035); or they fear the pain will have an impact on their professional activity: *“If I resume exercising and sports and then I hurt myself I will no longer be able to work*.*”* (line 2532).

False beliefs: Not all participants recognize PA as an integrant part of low-back pain treatment: *“Physicians who prescribe walking for low back pain*: *it’s useless”* (line 1713). In that case PA is perceived as a factor aggravating the pain: “*Playing sports is probably not great for my back*. *We keep jumping around so I think it might not be so great and often after a game my back hurt*, *and I think that’s what caused it”* (line 1407). Loading was often brought up by people with CLBP as an aggravating factor for pain: *“When I go grocery shopping*, *I can’t carry heavy things*, *for example 6-packs of milk bottles are impossible to carry*, *I must take the bottles one by one”* (line 1584); *“Standing still*, *without moving much*, *walking*, *sports*, *exercising*, *physical things that make the pain worse*. *Carrying a load such as a computer*, *groceries*.*”* (line 94).

Lack of perceived benefits: It appears that several people with CLBP tried to resume an exercise routine, but this experience was fruitless, aggravating the pain or not bringing any improvement: *“Sometimes I try to exercise and then I’m in pain*, *looking back had I known it would hurt I would probably not have done it”* (line 2037), *“my primary care physician recommended swimming*, *I did not know that breaststroke was not adapted to low back pain*, *since that is the only way I swam I was in pain and I gave up*.*”* (line 960). This sometimes leads to false beliefs with people with CLBP believing that PA can be harmful: *“It can be harmful*, *I give you an example*: *I have a colleague with low back problems*, *similar to mine*, *and she loves to take step classes*, *but each time she exercises too much*, *she is in pain but continues*. *I think she should stop*, *it is quite dangerous for her”* (line 378).

False interpretations: Some people with CLBP reported misunderstanding the terms used by the medical team: *“The term chronic low back pain*, *makes me think of recurrent back pain… it is frequently used by physicians*, *I have the feeling it is rather a “catch-all” term”* (line 90). Interviews showed that certain people with CLBP wrongly interpreted their additional examinations: *“I limited my activities*, *additional examinations for my acute back pain revealed a herniated disc*, *and spinal osteoarthritis …”* (line 24). Some people interpreted their pain as a sign of disease severity: *“I was really scared*, *I felt this close to being paraplegic*, *my back could not hold my weight*, *at 41 it was quite a shock*.*”* (line 246).

#### Socio-environmental and occupational barriers

Lack of time: This is one of the main socio-environmental barriers. Programming PA in a daily agenda requires scheduling efforts: *“but it’s impossible*, *these classes are at 6pm and I am often still at work*, *so I miss a class*, *really it was quite complicated and I did not go further”* (line 410). People with CLBP who have long working hours bring up the difficulty in finding time to exercise: *“Sometimes the days are just too short*. *When one starts at 6 am and finishes at 8pm it is …”* (line 1793); *“I’ve been told that swimming would help*, *but I don’t have any time*. *Time is the issue”* (line 37).

Occupation: Working is often reported by people with CLBP. Some see it as a triggering or aggravating factor of their low back pain: *“Yes I do think that working (well really there might be a genetic part) but work had to…Then at first we did not have all these technical aids*, *automated beds*, *Hoyer patient lifts*, *transfer stands*, *we did not have all this equipment so of course it affected our health*. *This is why most of my colleagues from this generation have problems*.*”* (line 417). Job dissatisfaction was also reported as a barrier to exercise, just like the workload: *“What could prevent me from continuing my exercises would be to not to find another job*, *my goal is to find a job where I am happy to go to work in the morning*, *it must be your case I believe*.*”* (line 1287). Certain people with CLBP fear that their condition will have a negative impact on their work, mainly they are afraid of being laid off.

Some people with CLBP showed a lack of motivation to exercise after an intense workday, especially those with a physically demanding occupation: *“For me*, *my job is physically demanding*, *so in the evenings I don’t really want to exercise”* (line 1698).

False recommendations from healthcare professionals: Some people with CLBP cited medical prescriptions and recommendations from healthcare professionals that went against promoting PA for low back pain: *“In my case they stopped everything*. *The physiotherapist because the pain was too severe*. *They told me to stop exercising at home because it wasn’t right either…”* (line 2386); *“they told me*, *do you want to finish in a wheelchair*?*” “Continue like this and you will end up in a wheelchair; then it will be over*, *you will no longer be able to run”* (line 2491), *“Yes I tried to see if I could run again but I was told not to”* (line 78).

Family environment: People with CLBP are sometimes encouraged by their closed ones to rest, and they describe a rather paternalistic and protective attitude: *“In fact my wife tells me to go easy etc*., *you are going to hurt your back*. *My wife tries to limit my activities”* (line 558). Family members often have a patronizing approach: *“Don’t do this”*, *“Don’t chop wood or your back will hurt”* (line 1861*)*, *“My girlfriend gets more worried*. *She is more inclined to help*, *if I don’t feel good she tells me to wait and she will do it for her*.*”* (line 2160). In the long run this attitude can maintain people with CLBP in a vicious circle of physical inactivity.

Barriers less frequently reported: Other barriers to PA were less frequently reported: the monotonous nature of the exercises *(“I would like to exercise but after a while you get really bored*, *it is no longer fun*.*”* (line 2509)), the environment, absence of prior sporting activity *(“It really will be hard*. *Well if you never did any sports before*. *It is really hard to stick with it*. *I can see it every year*, *I try and then I give up*, *because…”* (line 1894)), anxiety regarding the diagnosis’ lack of precision *(“And before that I was completely discouraged*, *in fact you don’t know what you have*, *you are in pain but do not know why*. *Every night it is impossible to sleep*, *my wife can’t sleep either*, *anxiety sets in”* (line 1258)), lack of interest for physical exercise, poor weather, and unwillingness to exercise alone *(“If I don’t go walking it is because no one will come with me*.*”* (line 1385)).

### Facilitators

#### Physical facilitators

Very few people with CLBP interviewed thought that a back support could improve their level of physical activity: “It relieves my pain especially when carrying heavy loads” (line 1925), “It bring more support really” (line 1926).

#### Psychological facilitators

The will to engage in physical activity: A great number of people with CLBP interviewed want to engage in PA, and some do in spite of the pain *“even the permanent pain does not prevent me from doing things*. *At home I do a lot of DIY”* (line 1681), *“I would rather do something else*, *even walking for half-hour even if I will be in pain*, *rather than stay put and do nothing*.*”* (line 1721). This will to exercise also seems related to the positive image of PA: *“When you see people who are well and do not exercise I feel…*. *It makes me sick”* (line 2060).

The desire to recover their prior physical aptitude / level of physical activity: Still related to the impact of pain on their daily life activities, people with CLBP expressed their wish to resume activities they had to stop due to the low back pain: *“Being able to resume leisure activities I used to do*, *being able to do them again if possible”* (line 1964); and getting back to their previous health status: *“I just want to get back to what I had before*. *Nothing more”* (line 1876).

#### Socio-environmental facilitators

Supervision by healthcare professionals end teaching people with CLBP to perform self-exercises: Many people with CLBP shared their fear of executing the wrong exercise movements and were more willing to engage in exercise therapy if it were monitored by healthcare professionals, who would ensure that the exercises were done correctly: *“At home we might be doing the wrong movements*, *or gestures we should not do*, *that is why I think it is better to be supervised by healthcare professionals”;* (line 1837) *“Plus it is important to have a coach who motivates us*, *helps us exercise*, *and corrects our postures”* (line 1738), *“At the gym*, *you do cardio for 45 or 90 minutes*, *you go there and cycle*. *You get bored and continue*.*”* (line 1840).

PA follow-up: Regular monitoring of PA practice seems to be a great source of motivation: *“There is increased motivation behind*. *Thinking that after two months we will see progress…*.*it is a type of motivation”* (line 1888). Only one people with CLBP expressed an opinion regarding follow-up practical modalities: *“A follow-up*. *Even by post or a phone call*. *Even an email follow-up”* (line 1850), *“That way we can fix ourselves a goal and try to stick to it*. *It really depends on one’s motivation really*.*”* (line 1853).

Group practice: The notion of group exercise was regularly reported: *“It is always more motivating to be with someone*, *not necessarily a group*, *just a colleague*, *there is shared motivation*. *Because if you have to go alone you will find excuses for not going that night and pushing it back to the next day”* (line 1829). Family PA practice was also reported a few times: *“In the evening when she gets home not too late*, *we go for a walk”* (line 1870).

Multimedia support: There seems to be a certain reticence in using multimedia supports, people with CLBP are often critical regarding the type of exercises offered on these supports: *“Ok but only if there are good exercises*. *Because all the games right now on the Wii…”* (line 1920). Participants with CLBP see it more as additional supervision: *“I would say it is in addition to my routine*, *for example during busy weeks*, *I can do a half-hour of that thing*. *I can try to do it”* (line 1922).

Daily life obligations: Participants with CLBP sometimes report feeling “obligated*”* to engage in PA when it is part of daily life: *“Of course we do stuff me must do because one must earn a living*, *and then some like gardening because nobody else is going to do if for me”* (line 1980),

The notion of pleasure: From the focus groups, it emerged that the nature of PA plays an important role, and when it is a pleasurable activity for the people with CLBP, it improves adherence: *“It is really different to exercise to keep in shape like we do here for example when we are alone*, *compared to horse-riding*, *or playing sports*, *or walking around*, *for that I don’t need anything… It is obvious”* (line 2556); *“If there were mushrooms all year long I would walk all the time (laughs)”*; (line 2565).

#### Others facilitators

Other facilitators reported in group interviews and rarely brought up were: summer season (*“with the nice weather it is much easier”* (line 1822)), the will to feel better (*“If I felt better exercising I think I would stick to it”* (line 2513)), and explanations delivered by healthcare professionals on the benefits of PA for their low back pain (*“If they tell me that I need to do the same movements every morning for example during one hour because it will help my condition*, *I will do it and I will adapt”* (line 1947)).

The main results according to the two acquisition methods are summarized in Table 2.

**Table 2:** Main barriers and facilitators.

		Individual interviews (n)	Focus group (n)
Physical barriers			
	Low back pain	12	16
	Feeling of disability	5	5
Psychological barriers			
	Psychological impact of the pain	5	9
	Kinesiophobia / fear of pain	10	14
	Lack of perceived benefits	4	6
	Lack of motivation / physical occupation	9	12
	False beliefs	6	3
Socio-occupational barriers			
	Occupational PA: aggravating factor	12	1
	Erroneous medical information	6	4
Environmental barriers			
	Lack of time	5	9
	Immediate family	4	9
Psychological facilitators			
	Will to engage in PA		14
	Desire to recover previous physical aptitude		4
Socio-environmental factors			
	Supervision/Self-exercise training		10
	Follow-up		6
	Group practice		7
	Multimedia supports		4
	Daily life obligations		6
	Notion of pleasure		4
	Weather season		4

n = number of citations

## Discussion

This study unveiled that there are multiple factors influencing PA practice in people with CLBP, varied, and often intertwined together. Furthermore, they differ from one person with CLBP to the next. Nevertheless, it seemed important to better refine these factors in order to detect them early on and adapt therapeutic strategies.

Our study unveiled three main categories of barriers and facilitators: physical, psychological and socio-environmental. The main physical barrier was pain. Regarding the main psychological barriers, we found that kinesiophobia and lack of motivation were at the forefront. The main socioenvironmental barrier was having a physically-demanding work. The will to practice a PA and supervision by a healthcare professional were the main facilitators to PA practice.

In our study, pain appeared to be a major barrier to participants’ adherence to a PA program. However, the main effect of exercise therapy is not to alleviate pain but to bring functional improvement with decreased disability and incapacity. This unveiled also the lack of information delivered by healthcare professionals to people with CLBP regarding the expected benefits of this care management. This adds up to another barrier found in our study, which is the absence of perceived improvement when exercising. Improving the information delivered to people with CLBP regarding the expected benefits of staying active associated or not to therapeutic self-management sessions could be a relevant pathway. It would be important to ensure that people with CLBP have correctly understood the set objective, i.e. not to have their pain disappear but rather to learn how to cope with it to promote functional improvements. Damsgard *et al* reported in their 2011 study that there were two main criteria influencing staying active in spite of chronic musculoskeletal pain: benefits of physical activity, possibility of resuming a social life thanks to PA [23].

However, the role of pain intensity in fear-avoidance of PA should not be neglected as mentionned by Bunzli et al in their 2015 study [24].

Our study highlighted that physical barriers and facilitators appear to be minor compared to psychological barriers, mainly fears (kinesiophobia and fear of pain) and false beliefs (related essentially to physical activity prescribed by healthcare professionals, reported to increase the pain according to a great number of people with CLBP interviewed).

This could partly be explained by the fact that participants in our study had a slightly higher VAS score and false belief score than other French studies on populations of people with CLBP, on the other hand the Functional Independence Measure (FIM) was comparable [25]. This meets the fear-avoidance model of people with chronic pain developing avoidance strategies essentially based on positive punishment (PA leads to pain, which in turn progressively diminishes PA frequency until it stops completely) [10]. Since the care management of people with CLBP is based on the bio-psycho-social model [26], the principle of therapeutic self-management becomes highly relevant [27]. The therapeutic self-management supports, mainly written ones such as information booklets [27], deliver information to people with CLBP on their condition and its treatment. Some studies did already show, in acute low back pain, that adherence to PA was better when the verbal description of the exercises was associated with written guidelines [28]. Furthermore, graduated exposure to AP could be interested in these people with CLBP, even more so since they have a high score of false beliefs. Implementing individual or collective self-management sessions, appears like a good solution to improve compliance to physical activity in people with CLPB [27]. The efficacy of individual self-management in people with acute or subacute LBP seems to be effective. For people with CLBP, the effectiveness of individual education is still unclear [29]. The main challenge remains to implement self-management strategies in everyday’s practice.

The influence of family members was brought up both as barriers and facilitators in our study. This underlines the great variability of the role played by family members. Friends and family appear as a potential source of increased adherence to PA, but only if they are educated on the subject. The implication of family members is even more important because the implementation of a regular exercise routine is often perceived as a great disruptor for the daily life organization, which is sometimes why patients give up their PA routine. Involving family members in the care management of people with CLBP, could alleviate certain barriers, especially lack of motivation.

The positive role of social support on PA adherence was already reported in the literature [30]. However, in 2009, Medina-Mirapeix *et al* showed that the influence of social interactions was more important than family support in regards to PA adherence in people with chronic pain [11]. Thus, this fact seems important to consider, especially since in 2013, *Bunzli et al* conceptualize the experience of “biographical suspension” in people with CLPB [31]. Family members could also have a negative impact in reinforcing sick behaviors [24].

It would be interesting to put an emphasis on the relevance of group exercise, which appeared in our study as a major facilitator.

Structured and supervised PA associated with people with CLBP education on self-exercises at home seems to be a source of motivation for several people with CLBP in our study as well as in the literature [32]. Hayden *et al* found in their study a better efficacy of the supervised exercises with an individualized program vs. simple exercises, associated with decreased pain scores and functional improvement [33]. Bronfort *et al* reported in 2011 increased satisfaction with supervised exercises, vs. home-based exercises [34]. The nature of the exercises taught to people with CLBP also seemed to influence their adherence, this is also confirmed in the literature [8]. The notion of pleasure in PA must also be accounted for. In light of these results, it seems relevant to initially propose an individual management, in order to offer a personalized exercise program for each person with CLBP.

The use of multimedia does not seem to appeal to most participants interviewed in our study. However, the emergence of new technologies such as virtual reality seem quite interesting for further exploration, to promote people with CLBP adherence to PA thanks to their recreational aspects. Results reported in the literature diverge. Miller *et al* concluded in their 2009 study that the use of video supports, such as DVDs was useful, mainly for the time period between two rehabilitation sessions [35]. Studies were conducted on “serious games” in people with chronic pain [36], but no game was specifically validated in people with non-specific CLBP [37].

The importance of follow-up by healthcare professionals is underlined in our study. Some people with CLBP explained that a follow-up helped them to set objectives. Coppack *et al* did not find any significant difference regarding adherence to an exercise routine between a group of people with CLBP who received set objectives to reach and another one who only had to do exercises supervised by a physiotherapist [38]. Liddle *et al* did highlight in their focus group that people with CLBP were in favor of a structured exercise program associated with personalized advice and follow-up [39]. According to the transtheoretical approach model developed by Proshaska [40], acquiring a positive behavior or changing a problematic behavior requires several steps, over a variable period of time. An appropriate follow-up by a healthcare professional would enable, in people with CLBP, to adapt the interventions to one’s exact stage of behavioral change.

The attitude of healthcare professionals is also an important issue [8]. This study underlines the relevance of offering, on top of education, a specific training dedicated to healthcare professionals on self-care for CLBP and PA, in order to improve the quality of the information delivered to people with CLBP and limit the transmission of erroneous elements. The objective being for people with CLBP to acquire the bases for coping with their CLBP. Nevertheless, to reach this level of autonomy, it seems that people with CLBP need comprehensive information on their condition and advice on adapted PA [41], in order to understand the efficacy mechanisms of the treatments proposed and increase their compliance [42]. In 2010, Crowe *et al* showed that patients developed coping mechanisms, based on their own experience and advice from healthcare professionals [43]. Darlow B *et al* reported in a systematic review of the literature that attitudes and beliefs of people with CLBP were closely related to the attitude and beliefs of their consulting physician [44].

Our results match those of the literature review proposed by Slade *et al*. [14]. People with CLBP show individual preferences regarding the type of exercise, and the playful nature of the latter would seem to increase PA compliance. The importance of the physician-patient relationship and subsequent communication was also highlighted in their article as a factor improving the impact of therapeutic education on the people with CLBP. PA facilitators identified in their study and ours were similar (exercise supervision, follow-up, individually-tailored exercise programs). This is also the case for barriers (lack of time, kinesiophobia). However, in our study no financial barrier was identified. The review of the literature published by Jordan *et al* also noted an improved exercise compliance when the program was supervised or tailored to individual needs, and when patients were taught self-management techniques [45].

### Limitations

Our study bears a certain number of biases. The main one being the absence of results’ generalization, the sample of people with CLBP in our study was not designed to be representative of the general population. The focus group method can also be a bias in itself, comparatively to the individual interview method, if the group interaction is less than satisfactory. It is also important to note that most people with CLBP recruited were involved in a functional spine rehabilitation program and thus presented with a higher functional disability level. Furthermore, these people with CLBP followed in a PM&R department tended to be better informed on their condition and the relevance of PA compared to the general population, which might have influenced the results. In the table listing the characteristics of participants recruited we can also note that men were predominant in our study.

### Implications

Our study unveiled the main barriers and facilitators to PA in people with CLBP. All these results converge towards defining a personalized approach for treating people with CLBP via self-management. This will help identify barriers and facilitators that vary from one person with CLBP to the next, and consequently adapt the care management to each individual person in order to promote the best PA compliance by reinforcing facilitators and correcting barriers. Encouraging the autonomy of people with CLBP with personalized self-exercise programs at home could also allow a reduction of costs related to this condition. This study could be a preliminary step for a wider-scale study to evaluate the efficacy of therapeutic education sessions on the compliance to PA of people with CLBP.

## Conclusion

Barriers and facilitators to PA practice in people with CLBP identified in this qualitative study could lead to new care management strategies, through self-care programs and supervised physical sessions that could lead to decrease barriers to PA and reinforce facilitators. It seems essential to assess barriers and facilitators, which are specific for each person with CLBP, at early stages in order to implement an individualized rehabilitation program.

## Supporting information

S1 File Ethics committee authorization. (PDF)

S2 File Interview guides. (PDF)

S3 File Verbatim individual and focus group interviews. (PDF)

S4 File Consent procedure. (PDF)
