# Supplementary material for: Facilitators and barriers to physical activity in people with chronic low back pain: A qualitative study
Source: PLoS One. 2017 Jul 25;12(7):e0179826. doi: 10.1371/journal.pone.0179826 (PMC5526504; doi:10.1371/journal.pone.0179826)
Supplement: S1 File — (PDF) [file pone.0179826.s001.pdf]

Comité d'Ethique des Centres d'Investigation Clinique de l'inter-région Rhône-Alpes-Auvergne

Dr Jean-Luc Cracowski
Chair
Centre de Grenoble
IRB n°00005921
cic@chu-grenoble.fr
Tél. 04 76 76 92 60
Fax 04 76 76 92 62

Dr Christian Dualé
Chair
Centre de Clermont-Ferrand
IRB n°00005891
cduale@chu-clermontferrand.fr
Tél. 04.73.17.84.18
Fax 04.73.17.84.12

Dr Behrouz Kassaï
CIC Lyon
bk@upcl.univ-lyon1.fr
Tél. 04 72 35 72 31

Pr Hervé Decousus
CIC Saint Etienne
cic@chu-st-etienne.fr
Tél. 04 72 12 08 26
Fax 04 77 12 78 20

Inserm
Institut national de la santé et de la recherche médicale

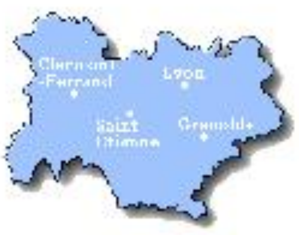

Grenoble, le 22/03/2012

Cher confrère,

Nous vous prions de prendre connaissance de l'évaluation de votre projet présenté au Comité Technique du CIC en date du 22/03/2012. Cette décision a été rendue après revue de votre projet selon la loi Française sur la recherche biomédicale [1] et la Déclaration d'Helsinki de l'Association Médicale Mondiale [2].

[1] Chapitre Ier du titre II du livre Ier de la première partie du Code de la Santé Publique relatif aux recherches biomédicales.
[2] Déclaration d'Helsinki de l'Association Médicale Mondiale. Principes éthiques applicables aux recherches médicales sur des sujets humains [http://www.wma.net/f/policy/b3.htm].

Avec nos sentiments les meilleurs.

Dr Matthieu Roustit – Pharmacien Dr Jean-Luc CRACOWSKI - Médecin Délégué

Date de la réunion	22/03/2012
N° IRB	5044
Membres du CT présents	Bettega Georges, Paris Adeline, David-tchouda Sandra, Faure Patrice, Gonnet Nicolas, Jean-Luc Bosson, Ego Anne, Sylvie Rossignol, Cracowski Jean-Luc, Roustit Matthieu, Pelloux Herve, Christophe Mendoza
Membres du CT excusés	Benhamou Pierre-yves, Sabbah-guillaume Helene
Rapporteur du projet	Anne Ego
Titre du projet soumis	COUDEYRE : Les freins à l'activité physique chez les patients lombalgiques chroniques
Nom de l'investigateur principal	Emmanuel COUDEYRE
N° de dossier IRB	CE-CIC-GREN-12-03
Service	CIC
CHU de rattachement	Grenoble
Autres destinataires du courrier	Emilie Richard ; C Duale

CRITERES D'EVALUATION DU PROJET	Valide	Non valide	Imprécis	NA
**Ethique médicale**				
Justification de l'étude (bibliographie)	☐	☐	☒	☐
Caractère courant de la pratique clinique	☒	☐	☐	☐
Utilisation courante des médicaments / dispositifs	☐	☐	☐	☒
Caractère courant de la surveillance	☒	☐	☐	☐
Définition du critère de jugement principal	☐	☐	☒	☐
Justification du nombre de sujets inclus	☐	☐	☒	☐
**Autres éléments éthiques concernant le participant**				
Formulaire d'information	☒	☐	☐	☐
Accord du patient pour l'exploitation des données	☒	☐	☐	☐
Procédure d'anonymisation des données	☒	☐	☐	☐
Déclaration nominative CNIL	☒	☐	☐	☐
Autres (à préciser) :	☐	☐	☐	☐

Cadre règlementaire de la recherche	
Recherche biomédicale (RBM)	☐
Recherche sur les soins courants	☐
Collection biologique	☐
Recherche observationnelle hors RBM	☒

Décision du CECIC	
Avis consultatif favorable	☒
Réserves de modifications à soumettre au CECIC	☐
Réserves majeures en termes d'éthique	☐
Reclassement en RBM ou équivalent	☐

Commentaires

Avis éthique consultatif favorable, avec néanmoins quelques points à préciser :

- L'étude semble avoir débutée en septembre 2011
- Les objectifs pourraient être exposés en début de protocole et de manière plus claire. La stratégie d'analyse des données qualitatives de l'entretien et des données quantitatives recueillies à partir de différentes échelles ou scores spécifiques (FABQ, TAMPA, QUEVEC, Bacl Belief Questionnaire) n'est pas suffisamment explicitée.

Le formulaire d'information du patient est par contre rédigé avec beaucoup de précision et emploie un vocabulaire adapté.

Un avis éthique consultatif favorable a été obtenu le 22/03/2012 (CECIC Rhône-Alpes-Auvergne, Grenoble, IRB 5044.

Study ethics approval was obtained on 22 March 2012 (CECIC Rhône-Alpes-Auvergne, Grenoble, IRB 5044.
