# Supplementary material for: Facilitators and barriers to physical activity in people with chronic low back pain: A qualitative study
Source: PLoS One. 2017 Jul 25;12(7):e0179826. doi: 10.1371/journal.pone.0179826 (PMC5526504; doi:10.1371/journal.pone.0179826)
Supplement: S2 File — (PDF) [file pone.0179826.s002.pdf]

Interview guide n° 1 for individual interviews

→Tell me about your low back pain (you back pain, tell me more about how you are living with it)

LOW BACK PAIN

Knowledge: - Low-back pain

Fears and beliefs: - At the beginning? (Cause)
- What did you do to get better?
--- What do you mean by rest? How important is it for you?
--- How do you see your back pain progressing?
--- When you are in pain, what do you think about (what do you tell yourself about your low back pain)?

PHYSICAL ACTIVITY

Definition - What is physical activity?

Benefits/Risks - Generally, what impact does practicing a physical activity have on your health? And on your back pain?

- What is the place of physical activity in your daily life?

BARRIERS

General - How do you practice a physical activity?
--- What prevents you from having a regular physical activity?

Intrinsic factors

Medical*:* - How did you adjust your physical activity since your low-back pain started?

Psycho-social - How do you adjust your life habits when the pain gets worse?

Cognitive-Behavioral --- What level of activity do you keep during your low-back pain episodes?
--- How does your low back pain affect your morale, mood and physical state?
--- How does your back pain change your relationships with others?
--- What impact has your back pain on your behavior with others?

Extrinsic factors

Environmental

Social - In your environment, what facilitates engaging in a physical activity? And what limits it?
--- What are your motivations to engage in a physical activity or conversely to not engage in a physical activity?

--- Which conditions are necessary for you to engage in a physical activity?

Occupational impact:
- How does your back pain impact your work?
- Does the level of work-related physical activity have an impact on your back pain?

--- In your agenda, what place to you dedicate to a physical activity? How were you able to adjust your agenda since the beginning of your back pain?

Impact of friends and family:

- How do people in general perceive your back pain?

--- How do your closed ones perceive your back pain? What advice can they give you?

--- How do healthcare professionals (physician, physiotherapist, nurse..) help you manage your back pain?

--- How did you seek information, on your own, regarding managing low-back pain?

<u>Interview guide n° 2 for individual interviews</u>

General and demographic information:
Gender, age, occupation, leisure activities, level of physical activity, medical, surgical and family history (low-back pain), ongoing pharmaceutical treatments

Low-back pain history
1/ Progression duration, recurrent/permanent episode,
number of episodes.
2/ duration of the ongoing episode
3/treatment

<u>Initial guideline</u>: Tell me more about your low-back pain.

1/ BARRIERS RELATED TO THE LOW-BACK PAIN

1. <u>Knowledge</u>
2. <u>Fears and beliefs</u>

2/ BARRIERS RELATED TO THE PHYSICAL ACTIVITY

1. <u>Definition</u>
2. <u>Benefits/risks</u>

3/ OTHER BARRIERS

<u>1. Intrinsic barriers</u>

--- *Medical*
--- *Psychological*
--- *Cognitive--behavioral*

<u>2. Extrinsic barriers</u>

--- *Environmental*
--- *Social*

4. <u>Occupation impact</u>

5. <u>Friends and family</u>

Focus group interview guide

- Tell us about your low-back pain (history, duration, treatment)
- What is the impact on your daily life?
- What was the impact on your job?
- What is physical activity (PA) for you? (relationship to PA?)
- What prevents you from regularly engaging in a physical activity?
- How did you change your PA habits since the onset of your low-back pain?
- What would help you/motivate you to engage in a physical activity? (Environment? Love ones? Back brace? Weather? Multimedia support?)
