# Supplementary material for: Facilitators and barriers to physical activity in people with chronic low back pain: A qualitative study
Source: PLoS One. 2017 Jul 25;12(7):e0179826. doi: 10.1371/journal.pone.0179826 (PMC5526504; doi:10.1371/journal.pone.0179826)
Supplement: S3 File — (PDF) [file pone.0179826.s003.pdf]

Verbatims consolidés le 02 janvier 2017

ENTRETIENS INDIVIDUELS

Entretien n°1

Moi l'activité physique j'en pratique pas parce que je n'ai pas le droit pour le moment, peut être qu'après j'aurais le droit.

Déjà moi dans mon boulot oui ; Je ne peux pas dire que c'est de l'activité physique physique mais bon. Quand on arrive à la période d'hiver pour moi oui c'est physique. Porter des pneus toute la journée c'est pas … le soir on n'a pas envie de faire de la gymnastique !

On marche mais comme je disais à Romain on piétine plus qu'on marche. Après le soir j'essaye d'aller marcher un peu.

Oui oui oui j'essaye, vu que j'ai un jardin j'essaye d'y aller à pied.

Le jardinage oui c'est de l'activité physique, les travaux ménagers non, passer l'aspirateur (rires)

Ben oui, je pense oui, que l'activité physique en général peut améliorer la santé. Néfaste non pour moi non, je ne pense pas

Déjà ça me ferait du bien au niveau des muscles, des cuisses, des abdominaux, du dos. C'est pour ça qu'on m'a conseillé de faire de la piscine. On va essayer d'y aller.

La tout de suite je pourrais, mais avant je ne pouvais pas, j'avais trop mal au dos, je pouvais pas, il y a des journées je ne peux pas à cause de la douleur. Quand vous avez vraiment mal vous n'avez pas envie d'aller courir.

Oui j'ai freiné mes activités, depuis que je me suis bloqué le dos net, que j'ai passé des examens et qu'ils m'ont dit que j'avais une hernie discale, une lombalgie arthrose …

Je me suis bloqué une fois, après c'est reparti, puis après le deuxième épisode j'étais au boulot à Issoire et après je ne pouvais plus, je suis rentré.

J'ai fais des séances de massage mais bon…

Si j'ai mal au dos je ne fais pas d'activité ça s'est sur, je ne vais pas aller au jardin et bêcher, c'est hors de question ! (rires) j'y arrive pas. J'ai 2 gamins si j'ai mal et qu'ils veulent jouer j'ai mal au dos je ne peux pas. C'est pas que je ne veux pas, c'est que je ne peux pas .

Je pense pas, non, c’est pas que je ne suis pas capable, dans ma tête je me dis que peut être aller à la piscine ca me ferait du bien mais …non je suis pas apte.

J’ai peur de faire un exercice et que ça aggrave quelque chose.

Silence Ah. Vu que j’ai jamais essayé je peux pas le savoir, je peux pas vous dire.

Penser mais pas essayer, ça aurait peut être fait du bien je dis pas le contraire.

Bah déjà faire du sport non, c’est sur. De la piscine on me l’a déjà dit, mais j’ai pas le temps. Le temps c’est le problème.

Il y a des périodes ou c’est plus calme, je pourrais. Mais la on va arriver à une période d’hiver j’aurais pas le temps. Il y a des gars ils portent des pneus toute la journée, c’est sur que quand ils rentrent chez eux ils mangent et puis hop au lit ! Pourtant c’est dommage je suis sur que ce serait bien d’aller à la piscine pour se détendre après une bonne journée de travail. Avant j’allais chez un kiné à la plaine, des fois quand j’avais mal je l’appelais et puis hop, il avait un petit bassin avec un lit, ça me massait le dos. J’en suis presque à 1000 séances de kiné ! quand même (rires).

Il y a des jours, je lui dis au chef, je lui dis aujourd’hui je peux pas. Je prends la ceinture lombaire. Il y a que ça qui me soulage, je la met jusqu’à midi puis après c’est chaud c’est décontracté.

L’activité du boulot c’est sur qui si on fait pas attention oui ça aggrave, si on fait attention et qu’on travaille correctement non, en prenant des postures. J’ai appris dans un stage d’entreprise geste et posture, mais c’est vieux ça. Ca permet d’apprendre à soulever une charge, un pneu.

Oui , c’est sur ça soulage. J’en vois plein qui travaille chez nous quand je les vois travailler ….

Ma femme oui des fois elle me conseille, parce qu’elle aussi elle a un métier un peu, mais …

« Porte pas ça comme ça ». Mais je sais ce que j’ai à faire, moi les conseils maintenant !

Non chacun se débrouille ! Par contre moi je leur donne des conseils.

« Bah de plier les genoux, de mettre les genoux parterre », je leur dis « tu forceras deux fois moins ! »

Y’en a un qui joue avec moi à la pétanque, il doit passer une IRM parce qu’il a mal au dos, je lui dis faut y aller faut pas avoir peur ! Puis si il faut faire de la rééducation physique faut y aller ! Mais l’activité physique il en fait pas.

Je dis il faut marcher, même qu’une demi heure, mais il peut pas, le mec il a pas fait de sport depuis des années, il fume comme un pompiers…

Oui quand je suis en vacances, le soir c’est dur après au niveau physique.

J’ai rien senti. Même en vacances quand je marche je n’ai pas mal au dos.

Même en période douloureuse si je vais marcher je n’ai pas mal au dos.

Régulièrement je veux bien mais faut avoir le temps !

Non je ne me suis jamais informé sur comment on soigne le mal de dos, non jamais. Mon gamin il a mal au dos des fois je lui est dis : » je t’ai ramené un bouquin tu vas le lire »

Je viens de changer de médecin (parce que l’autre est parti à la retraite) et après le kiné ben non, je sais pas quoi dire là.

Par des médicaments j’ai pas le choix. Si y a la douleur faut prendre des médicaments, après c’est faire de la rééducation, des séances de massages.

Enfin c’est pas la première fois qu’on me dit qu’il faut faire de la natation mais quand il m’a dit qu’il fallait que j’y aille trois fois par semaine je lui est dit j’aurais pas le temps, vu mes horaires je peux pas !

Oui j’ai essayé de voir si je pouvais recourir mais on m’a dit non. Même quand j’essaye de jouer au foot avec les gamins quand l’autre il rappelle à l’ordre je m’arrête. On va voir avec cette semaine puis la continuité de ce que j’ai appris la, et la piscine peut être que ça va faire quelque chose, on verra après !

Ben oui quand j’ai mal au dos ça retentit sur le moral, c’est sur que des fois le moral est pas très bon.

Je suis moins motivé c’est sur pour travailler déjà ! Et puis j’ai 2 enfants je peux pas jouer avec … c’est dur d’avoir mal au dos

Entretien n°2

La lombalgie je pense que c’est une douleur dorsale, répétée…

C’est un mot qui est très fréquemment employé par les médecins, j’ai l’impression que ça fait « fourre-tout » un peu pour moi, oui.

Des lombalgies ce sont des douleurs j’imagine dorsales, mais non je ne suis pas aller voir sur doctissimo ou je ne sais quoi, voilà.

La station debout, la sensation de piétiner, la marche, le sport, enfin l’exercice, les choses physiques aggravent. Porter un poids type : un ordinateur, des courses. La voiture de façon évidente. Et ce que j’appel le «twist» j’ai pas de mot en français pour dire ça.

Les rotations mais ça peut être quelqu'un qui me prend la main droite et qui va la tirée vers lui en m’emmenant vers lui, comme un enfant qui va se mettre à serrer la main pour montrer quelque chose, ce genre de choses.

Ah oui ! J’essaie de faire en sorte que ça ne me fasse pas mal.

Non non je n’appréhende pas plus que ça…C’est juste que je fais naturellement attention voilà.

Ce qui pourrait me soulager c’est le fait d’être allongée, oui la station allongée. Si vraiment j’ai mal je m’allonge et la douleur se calme. Et évidemment les médicaments.

Alors j’essai quand même de m’allonger le moins possible. Pendant longtemps j’ai fait ça, c’est à dire de ne pas m’allonger du tout.

C’était les conseils oui qu’on m’avait donné. Et en fait je travaillais et ça a été une espèce de boucle, une spirale infernale parce que effectivement je tenais debout et à 18 heures je n’avais plus aucune possibilité d’être debout. Donc là les médecins ont fait du rétropédalage et ont dit au contraire « il faut vous reposer ! »« Trouvez des moments pour vous poser ». Donc ce que je fais généralement le matin j’ai une activité que je qualifierai d’à peu près normale : tournicotant dans la maison faire 2-3 courses etc . Et en revanche l’après midi d’être assise et de faire attention, si vraiment j’ai mal par exemple je m’allonge 2 heures. Je ne dors pas c’est juste la position allongée. Ce qui me permet d’avoir du jus pour le soir quand tout le monde rentre être a peu prêt correcte et dynamique. Et le soir je me rallonge assez tôt.

Oui c’est pas automatiquement à 14 heures je m’allonge !

Douleurs est synonyme d’aggravation, non je pense pas, les 2 sont complètement distincts. Non.

Oui tout à fait oui, le curseur est différent je pense en fonction de chaque personne. Comme j’étais très active avant physiquement dans la mesure ou c’est vrai que je faisais beaucoup. On habitait en centre ville à Clermont, je marchais beaucoup, je faisais même tout à pied et on marchait en plus tous les week end. Donc évidemment aujourd’hui mon activité physique elle est réduite à peau de chagrin ! Parce que voilà parce que je suis bloquée d’avantage, donc mon activité physique en ce moment elle se résume à monter des marches, marcher un peu mais vraiment très peu, parce que si je marche beaucoup la douleur se ré installe immédiatement. Mais je m’efforce à maintenir un semblant d’activité physique, voilà.

Oui je considère les travaux ménagers et le jardinage comme de l’activité physique mais alors ça je suis incapable de faire ! c’est à dire que si par exemple je passe l’aspirateur dans la

maison, pour l’avoir fait une ou deux fois je sais que ça va me couter 2 jours complets allongés. Le jardinage j’adore ça hein, mais heu alors je le fais assise, je désherbe assise ou ce genre de chose mais je peux pas me pencher, ça s’est absolument exclu.

Oui d’une manière générale l’activité physique améliore la santé oui, mais toute activité n’est pas bonne.

Ce qui m’empêche de pratiquer une activité physique régulière c’est ma douleur. Oui parce que je suis plutôt d’un naturel actif

Donc j’aimerais pouvoir en faire plus.

J’ai arrêté la marche à partir du moment ou mon mari a commencé à faire la voiture balai, c’est à dire qu’il a du venir me chercher parce que j’étais incapable de continuer, de venir me chercher en voiture j’entend. C’était il y a 2, 3 ans, donc une fois deux fois trois fois, la quatrième, bon je ne vais plus marcher ou je vais en faire très peu parce que même en réduisant la balade à une demi heure, au delà c’est exclu de continuer.

Oui évidemment ce serait le rêve de reprendre !

J’ai la chance au niveau du travail dans la maison d’être beaucoup aidé. Il y a quelqu’un qui venait avant et qui vient maintenant, donc ça ça n’a pas foncièrement changé. Ce qui a changé ce sont les courses de façon évidente, et puis la vie de tous les jours de ne pas partir en voyage parce que si on part en voiture on l’a fait une ou deux fois, et du coup j’ai tellement mal à l’arrivée qu’une fois sur place je ne peux plus rien faire…donc voilà ça a modifier c’est sur notre quotidien.

J’ai vraiment essayé de conserver une activité physique, parce que je pense avoir du caractère, on va dire ça comme ça et j’ai essayé encore la semaine dernière d’aller marcher à la demande du docteur C., et je l’ai fait 3 jours. Et au 3ème jour la douleur était telle que j’ai du prendre 4 ou 5 actiSkenan en plus de la dose. Donc le lendemain j’y suis retournée en me disant on va quand même ressayer, c’est insupportable ! Donc je veux bien beaucoup de chose, mais c’est pas tenable donc j’ai arrêté et je vais reprendre une fois que l’épisode douloureux sera passé et reprendre petit à petit peut être 5 à 10 minutes par jour.

A mais pour moi ça semble évident, sauf que au jour d’aujourd’hui c’est un peu le chat qui se mord la queue, parce que j’essaye de le faire, j’attend jamais dans mon lit à attendre que ça passe. Mais la limite est très rapidement franchie, j’appréhende par exemple je dois aller faire de la rééducation, je sais pas comment je vais physiquement tenir…

Sur le fond je suis d’accord avec vous, à condition, mais je n’arrive pas à voir comment y arriver.

Ca a un retentissement sur le travail, parce que je travail pas !

Mon travail n’est absolument pas à l’origine de mes douleurs, les aggraver certainement, dans la mesure ou je suis debout beaucoup quand on est prof, par principe, même si j’essai de m’appuyer sur le bureau, parce que être assis sur une chaise pour moi ça c’est absolument pas convenable. Parce que oui il faut que je porte mon ordinateur, mon cartable, les copies, fin… il ya toujours cinquante mille trucs et même si les étudiant sont sympa et m’aide. Je l’ai fait pendant 3 ans et la je suis arrivée à une limite ou c’est absolument ingérable.

Mon entourage … alors oui ils me comprennent, je pensent qu’il sont extrêmement compréhensif sans tomber dans le, je sais pas comment dire, ce que je veux pas c’est être une maladie vous voyez ? Je m’appelle A., au boulot personne ne savais que je souffrais du dos jusqu'à ce que j’arrive avec une canne, et à la maison ou en famille bien sur on en parle surtout qu’en j’en ai marre mais on s’est adapté voilà, on a fait en sorte de s’adapter et de vivre quand même parce que la vie s’arrête pas et heureusement. Pendant 3 ans on n’est pas du tout parti en vacances, alors on a la chance d’avoir une maison de campagne, on s’installait l’été, les enfants partaient avec leurs copains. Parce que c’est vrai que pendant un certain temps il était exclu que je parte en voiture par exemple.

Ah non non , ils me laissent gérer ça, ils savent généralement que je suis active, donc quand je dis j’y arrive pas c’est que vraiment je n’y arrive pas !

Les conseils peut être à mon frère quand je le vois porter son fils, et il dit qu’il a mal au dos alors je lui dit forcément si tu pliais tes jambes

Vous voyez plutôt ce genre de choses que j’ai apprise, quand j’ai fait une rééducation à

Je me suis informée par le centre anti douleurs, par mon médecin, par des kinés, classiquement je dirais…

Internet je dirais que j’évite parce que j’ai vu ce que ça donnait dans mon métier… Je trouve que un les sites de chat et tout ça les gens racontent tout et n’importe quoi je trouve ça assez peu fiable et je suis assez réticente à aller voir les choses médicales sur internet parce que comme j’ai vu ce que ça donnait dans mon boulot et le nombre de contre vérité qu’on pouvait voir, je me suis dit si c’est la même chose en médecine… J’aime beaucoup internet par ailleurs. Je pense qu’un médecin c’est un médecin voilà.

Oui j’essaie de suivre les conseils mais je n’y arrive pas c’est dur, à chaque fois je me retrouve face à l’échec…

Entretien n°3

La lombalgie c’est des douleurs lombaires…

Maintenant je pense pas qu’il y est une durée à cette maladie parce que j’en souffre depuis trop longtemps, et j’en vois pas le bout. Je dirai qu’il y a des périodes ou je souffre moins m’enfin c’est là, ça fait parti de mon quotidien en fait je suis obligé de gérer. Quand on peut oublier, je sais même pas si on peut oublier quand on mal au dos puisqu’on le vit. Mais comment on peut (hésitation) pensez à autre chose si on est obliger de faire attention dans nos gestes dans nos mouvements, enfin, c’est difficile, ça m’a traumatisé moi je crois ! (Sourire)

Je sais qu’au bout d’un moment, je me doute qu’au bout d’un moment, il va y re avoir de nouveau ce genre d’épisode et là je pense qu’il y aura certainement intervention et là je suis pas pressée.

Ce qui pourrait aggraver mes lombalgies c’est le port de charge lourdes, les transferts, ce que je faisais auparavant je peux plus le faire et je je … J’en suis tellement consciente que je chercherai même plus à le faire parce que comme je vous dit j’ai plus besoin de faire grand chose pour sentir que j’ai mal au dos donc … le port de charge lourde

Bien sur je veux dire ne serait ce que des petite choses qu’on fait à la maison, on est obligé de ruser, de porter moins lourds les panières de linges. Je me fais beaucoup plus aider qu’avant. Avant j’étais autonome je dirais, que maintenant je le suis beaucoup moins. Et puis à 40 ans c’est pas flatteur, j’ai vraiment eu une période quand j’ai fêté mes 41 ans clouée au lit, ça m’a vraiment mis un coup de… un électrochoc quoi de…

Ca a retentit sur mon moral, j’étais vraiment angoissée…

La position allongée me soulage en fait, un temps je pouvais plus rester debout enfin, à cette période qui était très lourde en douleurs – parce que j’ai quand même récupérer mais ça n’empêche que , je fais des choses que je pouvais plus faire effectivement.

J’essai de les faire, mais je me rend bien compte que ça me réveille la douleur en fait, donc je m’économise. Je fais encore des choses hein heureusement mais même quand je me baisse je suis moins spontané dans

Ben là actuellement quand on est en arrêt c’est sur que l’on a plus le temps de se reposer, du coup on dort un peu plus longtemps que quand on travaille effectivement.

J’avouerai que la position allongée en fait elle me va bien. Quand je suis assise trop longtemps ça me réveille des douleurs lombaires. La position debout aussi, rien que le fait de me coiffer, je sais pas si c’est que je me cambre mais je suis obliger d’attendre avec mes douleurs, de les gérer, essayer de me redresser un peu mieux. J’ai arrêté de porter des talons.

Je pense enfin je sais pas … je pense pas que douleur soit synonyme d’aggravation parce qu’en fait la douleur depuis notamment l’épisode très sévère elle m’a pas quitté, mais bon je vous avouerais que même l’accident de travail quand j’ai été arrêté un mois et demi quand j’ai repris c’était pas que j’avais plus mal, j’avais encore mal mais je me suis dit ça passera j’avais

quand même une ceinture lombaire des choses comme ça. Je me sentais presque invincible et en fait c'était certainement pas cicatriser ou il y a eu quelque chose ou mon employeur a peut être trop surchargé mon planning et puis moi j'y voyais peut être pas d'inconvénient puisque temps que ça allait ça allait jusqu'au jour ou j'ai été cloué au lit et la j'ai eu très peur

J'ai eu très peur je me sentais à la limite de la paraplégie, mon dos me portais plus, enfin à 41 ans ça choc quoi.

Sincèrement je pense que l'activité physique améliore la santé oui, après faut il pouvoir. Moi je sais que j'aurai besoin d'avoir un dos plus musclé ça c'est sur, parce qu'il a perdu du muscle parce que l'économise mais le souci c'est que je peux pas chercher à le remuscler tant que la douleur n'est pas passée en fait.

Je sais pas je voudrais me remuscler un petit peu, et le fait d'avoir quelque kilos, je voudrais me refaire une ceinture abdominale et dorsale, c'est ça qui me manque. Je ne suis pas excellente en natation, la piscine oui mais sous forme de kiné parce que c'est vrai que ça me faisait du bien

La kiné ça prend sur le temps et il n'y a pas d'évolution, ça guérit pas en fait, au bout d'un an et demi on en a assez, ça fait du bien quand on y est on a l'impression d'avoir moins mal ou d'être plus souple, après on sort, on se sent toujours un peu frêle du dos ça fait pas disparaître complètement les douleurs. C'est peut être aussi dans la tête mais si c'est dans la tête c'est peut être aussi dans mon dos. Des fois j'ai comme l'impression d'avoir comme une bulle qui demande qu'à craquer au niveau du dos.

Ne serait ce que l'entourage quand on parle on entend que la piscine s'est bon pour le dos, après je sais pas peut être que l'on s'enferme dans sa bulle, ou que l'on s'enferme dans sa douleurs.

Après quand j'ai repris le travail même si c'étais en mi temps thérapeutique on en a pour son compte donc on a envie de souffler, on manque de motivation, on a envie de se reposer plus que d'aller se redonner encore. Surtout que moi c'est un métier ou on fait des coupure et on reprend après donc j'ai pas vraiment, la avec le mi temps thérapeutique je servais les repas ou en accompagnement l'après midi donc ça laisse pas beaucoup de temps. Vous me direz votre jour de repos, mais le jour de repos ben du coup on en profite pour faire des petites choses dans la maison qu'on ne fait pas habituellement quand on travaille, surtout que moi je travaille un week end sur deux.

Bien oui je freine mes activités essentiellement liées aux douleurs, un peu d’appréhension…

C’est sur que je suis moins alerte, ça m’oblige à me reposer beaucoup plus, en même temps quand on est en arrêt ou en mi temps thérapeutique, on fond son quotidien sur ce rythme en fait.

Alors ça dépend quelle période douloureuse, la période quasimodo non. Après j’ai mal au quotidien, rien que le fait de me coiffer ou de porter des choses un peu lourdes… ça m’a nettement diminué.

Je vous crois, la marche c’est celle qui me ferait le moins peiner, ça décrante, c’est peut être la position debout et en mouvement, puisque assise c’est pas la position qu’on préfère, stagner debout dans une file d’attente la aussi c’est désagréable. La marche s’est bénéfique ça fait du bien

Oui l’étang de sceau ou des choses comme ça, mais je ferais une randonnées il y a quand même une différence. Une petite marche…

Le travail c’est ce qui m’a donné mon mal de dos et c’est ce qui m’empêche de travailler.

Mon entourage je pense qu’ils se sentent impuissant par rapport à ma situation. Ils me disent fait attention.

J’ai un cousin qui s’est fait opéré, après on sait que c’est le mal du siècle effectivement. Il y a mal au dos et souffrir du dos. Moi je dirais que je souffre du dos

Je suis mal placé pour donner des conseils, a part dire de faire étirement, ça fait du bien, mais le souci c’est que ça guérit pas…

Je dirais qu’on me soigne, mon médecin m’a conseillé la marche, j’y vais par beaux temps. Vous me demandiez aussi ce qui pouvait me faire mal au dos, il y a aussi la voiture. Quand je suis installé ca va mais le fait de montée descendre, ça aussi c’est quelque chose… Moi j’ai l’impression que mon dos il m’attend au tournant et c’est pas dans ma tête c’est réel ; Je sais ce dont je souffre, c’est moi qui le vit, je sais ce qui m’attend au tournant.

La marche c’est pas régulier, c’est de temps en temps, c’est plus appréciable quand il fait beau. Et puis quand il fait un temps mi figue mi raisin on a plus mal au dos, je sais pas si c’est l’humidité ou le changement de temps, ça reveille et amplifie les douleurs. Avec ma famille on va faire un petit tour on apprecie, mais c’est plus une promenade.

La plupart de mes amies exercent la même profession que moi et on a des horaires un peu compliqué. Avant que mon mal de dos s’installe j’avais une amie avec qui on avait envie de s’inscrire dans un club de gym et puis bon après j’ai eu ces problèmes de dos donc c’était plus d’actualité.

J’essai d’économiser et de grader ce que j’ai réussit à récupérer.

Après ma rééducation j’ai eu une période ou c’était massage, exercice. Après l’épisode extrêmement douloureux du mois de juin, quand j’ai repris la kiné c’était uniquement du massage avec cataplasme et ensuite, on a fait de la kiné en bassin, des mouvements dans l’eau, la température de l’eau fait que l’on est aussi quand même très a l’aise 40° je crois. Ca c’était positif mais si j’ai pas renouveler c’est que j’ai des périodes ou j’ai plus ou moins le moral et le fait de me dire que ça me fait du bien quand j’y suis (45 minutes de bassin), on se sent mieux et le fait de sortir du bassin, on grelotte donc ça contracte les lombaires mais ça guérît pas. Et une fois par semaine ça suffit pas.

Moi le souci c’est que quand je nage je nage cambré parce que je suis pas une bonne nageuse. J’ai l’impression que la position est douloureuse. Donc la piscine me ferait plus mal que bien mais la kiné en bassin me fait du bien.

Et une piscine municipal c’est pas un bassin de kiné, il n’y a pas les barres pour se tendre. Ce que j’appréciait vraiment c’était quand j’étais dans le bassin et de pouvoir tenir les barres et tendre mon dos.

Mon quotidien a été modifié comme cette année on est pas partie en vacances parce que après je souffre je préfère être préventive…

Entretien n°4

Ah la lombalgie, déjà il y en plusieurs sortes c’est pas toujours les mêmes. Quand c’est la douleur chronique et bien c’est une gène c’est une espèce de barre qui est toujours présente. Quand c’est le lumbago, moi j’appelle ça toujours le lance flamme dans le dos, comme une brulure.

C’est installé je vis avec, de façon chronique je ne prend rien.

Ce qui aggrave ma lombalgie c'est le travail, le port de charge, le quotidien, le jardinage je suis bloquée je fais pas ce que je voudrais, parce que j'arrête avant d'avoir mal en général. J'ai l'appréhension de la douleur, j'ai adapté mes activités de vie quotidienne

J'accorde au repos une grande place (rire) c'est à dire que quand je travaille le matin, je me lève le matin à 5 heures et demi donc quand je reviens il y a une sieste c'est obligé, je peux pas m'en passer. C'est parce que j'ai commencé de bonne heure. Quand je suis de l'après midi j'ai moins besoin de ça mais c'est pas en rapport avec mes lombalgies chroniques.

Personne n'a trouvé de solution pour me soulager. Parce que en fait j'ai paraît t-il 4 vertèbres qui sont en train de se souder et le rhumatologue m'a dit quand elles seront soudées vous aurez moins mal. Alors j'attends ! Mais personne n'a trouvé de solution. J'ai fait de la kiné, quelques fois j'ai fait des mouvements d'assouplissements et ça m'aide un peu quand je sens que j'ai une tension un peu plus forte si je m'astreint à faire ça un peu régulièrement je sens une amélioration, ça évite de s'aggraver.

Non mais il faudrait que je le fasse régulièrement, quand je pense qu'il y a un peu de tension alors la je vais le faire, sous forme d'étirement.

Rationnellement je pense pas que douleur soit synonyme d'aggravation, je pense que c'est un épisode et je me dis j'en ai eu d'autre je m'en sortirais, et puis c'est un peu déprimant. On a l'impression de repartir en arrière, en plus on a un métier ou on compte beaucoup les unes sur les autres, donc la elles sont plus que 2 et sa tracasse, ça me plait pas d'être arrêter. Et puis je suis anxieuse vis a vis de l'avenir aussi, parce que il faut travailler plus longtemps, et puis j'ai un métier ou il faut être efficace et faut être d'aplomb et des fois j'ai un petit peu d'appréhension.

Etre capable de faire ce qu'on a envie de faire, pas le sport j'aime pas le sport. Ne pas être limité, faire du jardinage, pouvoir marcher sans avoir trop mal pouvoir faire ce que l'on a envie de faire sans limite.

Oui oui l'activité physique peut amélioré la santé j'en suis convaincu.

Elle peut être néfaste pfff peut être, je donne un exemple : j'ai une collègue qui a des problèmes de dos un peu comme moi et elle adore le step, à chaque fois qu'elle en fait un peu

trop elle a mal mais elle continue. Moi j'estime qu'elle devrait arrêter, c'est un peu dangereux pour elle.

Moi je n'aime pas le sport, j'aurai mal en faisant du sport olala je m'arête !

ça dépend lequel, et ça dépend si c'est intensif, ou raisonnable

Plus de 3 fois par semaine, et si la personne à des problèmes… Enfin on peut pas généraliser comme ça.

J'aime pas. C'est à dire que j'aurai quelqu'un avec qui partir faire de la marche ça me plairait. Et par exemple aller faire de la piscine je me dit que c'est quelque chose qui serait bien pour moi mais sortir le soir et prendre la voiture toute seule j'ai pas envie. J'ai pas assez envie pour le faire. Un manque de motivation, une appréhension. Oui déjà à la base ça me plait pas mais en plus le faire seule c'est encore pire ! Faire les magasins seule ça me dérange pas, parce que j'aime bien. Rire

Ca a freiné mes activité Ah bah oui un petit peu parce que bon le jardin j'aime bien mais ça va déclencher la douleur, le travail aussi je suis en ambulatoire ça me plait bien mais je sais que je suis plus capable d'être dans un service.

Je fais que du repos, mon médecin m'a dit : pas de serpillère, pas de balai, pas d'aspirateur, rien, même ton lit faut pas le faire.

Il m'a pas donné de limite de temps et ça fait dix jour que ça dure… C'est chaise c'est la télé c'est les bouquin, mais on étouffe un peu. Même prendre la voiture bon je sors un peu mais c'est pas le top.

ça fait un moment que j'y pense à ça mais je le fait pas, pour les raisons que je vous ai donné tout à l'heure

Non pas du tout, je pense qu'il faudrait même que j'apprenne à nager le dos crawlé, je suis aller à aqualudique pour demander des cours puis me débrouiller toutes seule. Mais c'est pas possible, c'est des cours collectifs à des heures quand c'est à 6 heures le soir et que je travaille et que je peux pas y aller c'est pas rattrapable, enfin c'est compliqué alors j'ai pas été au delà mais je pense qu'il va falloir que j'arrive à ça.

La première fois que j’ai eu mal au dos c’est quand j’étais enceinte de mon fils ainé, il y a 32 ans, mais ça a été pendant la grossesse, ça a pas durer bien longtemps et après j’ai plus rien eu. Oui je pense quand même que le travail (bon il y a peut être le coté génétique) mais le travail a du… Puis au départ on avait pas toute ces techniques, les lits adaptés électriques, les leves malades, les verticalisateurs, on avait pas tt ça donc forcément on en a pâti. C’est pour ca que beaucoup de mes collègue de cette génération on ces problèmes.

Dans mon entourage ils me disent de m’économiser, ils me disent que ça, mes parents, mes enfants. Ils ne sont pas souvent la alors c’est un petit coup de fil, repose toi, prend soin de toi. Les collègues sont plus, par exemple quand je leur dit ça m’embète d’etre arrêter à cette période la elles me disent : « ne t’occupes pas de ça » »pensent à ton avenir » elles comprennent

Le médecin bien sur, le médecin traitant, les rhumatologues, voilà c’est tout. Mon ancien médecin traitant me dit faut faire de la piscine pour conserver mes muscles. Mais on m’impose rien. Est ce qu’on pense que parce que je suis infirmière je dois savoir, j’en sais rien mais…

Puis je pose pas spécialement de questions, quand j’y vais c’est que ça va pas bien et je demande à ce que l’on me soulage.

J’ai bien vu des reportages, mais ça ne m’a pas appris grands choses, parce que comme on avait des cours de manutentions pour l’économie du dos, des postures. Peut être que je me trouve bien informé alors que non… Les cures thermales aussi pourraient faire du bien.

Entretien n°5

La lombalgie c’est je vais dire des douleurs dans le dos, je vais pas dire des lombaires ça me semble tellement flagrant.

Ca peut être le nerf sciatique qui peut être un peu pincé, je dirais ça comme ça.

J’espère que ça se soigne ! Retirer moi pas tous les espoirs, après ça me semble compliqué pour moi parce que j’ai l’impression que c’est multifactoriel et que voilà. Je pense que ça s’aggrave d’ailleurs si on ne trouve pas des solutions rapides, à mon sens.

Ce qui aggrave je dirais pour moi l’usure à force d’utiliser le dos en faisant des mauvaises postures, je dirais la prise de poids, le manque de musculation.

Ce qui soulage c’est la reprise du sport m’avait fait du bien, mais pour que je puisse y arriver il faut que je trouve un moment ou je n’ai pas mal…les douleurs…

Mais il n'y a pas que ça l'organisation à la maison il y a plein de chose qui font que : Le manque de temps, un emploi du temps qui est pas forcément régulier et qui fait que ben je vais m'inscrire à un sport et dans la continuité je vais avoir du mal à le suivre. Ce qui me faisait beaucoup de bien c'était d'aller courir et je suis freinée par la prise de poids parce qu'il faut que je me traine.

C'est ce qui m'a mis un frein la tout de suite et le manque de temps même si je sais que ça c'est une mauvaise excuse.

Je me retranche derrière ça, et puis la j'ai vu effectivement qu'a travers les étirements j'avais trouvé cette activité et je trouvais que c'était une manière d'y retourner tranquillement et en fait dans certain mouvement qui sont assez spontané comme le fait de se baisser pour le coup ça me fait mal et puis je me dis que je sens que c'est très douloureux et qu'il faut que je passe outre certain mouvement. J'ai l'appréhension d'être complètement bloquée.

L'activité physique je dirais que sur du long terme ça me permettrait d'avoir moins mal, maintenant je sais que au moment où je vais pratiquer j'ai encore de la douleur qui vient se rajouter, je fais beaucoup de marche avec les jeunes avec lesquels je travaille.

Ca dépend un peu des jeunes, on peut faire des grandes promenades d'une heure et demi dans les bois, mais c'est pas régulier ca dépend de qui y va donc c'est pas… toute les semaine on va marcher et je me rend compte que c'est des douleurs à la hanche, c'est des douleurs sous les pieds et je sais que c'est douloureux encore après, mais pas une douleur c'est plus une gène. J'accuserai plutôt ça sur la prise de poids et les articulations il faut qu'elle supporte.

La prise de poids, je pense que c'est du au travail de nuit, je travaillais la nuit jusqu'à il y a pas très longtemps et bah l'organisation du travail ; c'est à dire que j'ai jamais trouvé mon rythme de nuit. J'avais fait les nuits il y a très longtemps sans prise de poids j'ai retravaillé de jour, puis j'ai repris les nuit il y a cinq ans et j'ai pris du poids tout doucement. Ni les conseille de la diététicienne ni rien n'y on fait, l'importance du sport aussi m'aidais à réguler tout ça et il se trouve que bah entre l'arrêt du sport, la reprise d'une formation… Le manque de temps

Pour moi l'activité physique c'est l'entretien de mon corps et un outil pour me vider la tête, la course à pied c'était ça, pas forcément courir beaucoup mais courir le temps ou je me vidais la tête. Quand je dis qu'évacuer le stress est important c'était un bon outil pour ça, et puis l'entretien de mon corps. Le jardinage beaucoup aussi, c'est de l'activité physique, je taille des haies beaucoup et je sais que c'est physique en terme de musculature c'est pas rien non plus.

Donc ce qui m'empêche ben en fait l'organisation mais on peut y palier à ça. Après j'ai peut être pas trouvé non plus celui qui me fallait : on me conseille la piscine je pensais aussi à ça et là il faut passer le cap d'y aller la je pense que …

Si j'y vais ce sera pour reprendre une activité c'est tout

Oui de s'organiser puis d'y aller

Mes lombalgies ont freinées mes activités je dirais que maintenant oui, je me rend compte que c’est pas un frein que je ne pourrait pas desserrer, il faut que je trouve quelques chose qui me soulage, l’eau c’est très bien hein pour le coup, parce que quand je vous dis pour le poids l’eau ça porte. Maintenant je sais qu’il y a des mouvements qui vont me freiner ça c’est certain. A la gym quand j’avais commencé l’année dernière d’aller à la gym, j’ai arrêté parce que j’avais du mal à tenir le rythme, au départ j’avais revenue que je ne savais pas si j’allais pouvoir tenir tous les mouvements. Il y avait cette réticence la à la douleur. Certain mouvement je les ai pas fait spontanément au début, je sentais voilà que … je contrôlais les mouvements, j’avais cette appréhension. Après les mouvements je les connaissais, je savais qu’il ne faisait pas mal. Et puis j’avais très mal à la hanche également à cette époque. J’ai l’impression que ça se déplace, ou j’ai mal au dos, ou j’ai mal à la hanche et plus récemment les 2

Quand j’ai très mal je ne fais pas d’activité, mais malheureusement j’ai l’impression qu’il va falloir que je conjugue avec ça, parce que j’ai pas le choix j’ai pas d’autre solution, et activité physique oui il va falloir que je reprenne pour plein de raison, il faut que je trouve, j’ai pas du tout retirer cette idée au contraire. Je sais que c’est une grosse roue de secours pour moi.

Je vous crois par expérience et c’est aussi le discours de mon rhumatologue, parce qu’un moment donné je lui ai demandé si je pouvais, je l’avais consulté juste avant les séances de gym et il m’avais vivement conseillé d’y aller.

Au travail j’ai l’impression que le mal de dos c’est quelque chose que l’on connait déjà toute, donc c’est compris de chacun. J’ai des bons appuis si je peux réfléchir à ma posture et on s’entraide, on a eu une formation. Et je sais aussi par expérience que des fois on n’a pas le temps de se positionner ou d’appeler quelqu’un pour nous aider et euh après si il y a un frein c’est par rapport à moi, je ne m’autorise pas certain mouvement. Généralement je préviens tout le monde en disant pour tel jour je ne vais pas intervenir. Oui c’est dérangeant par contre, ça me dérange moi et on a des gens qui ont des troubles du comportement et que l’on doit manipuler sous contrainte et ça je ne me l’autorise pas, c’est à mon détriment et j’essai de palier et de trouver des solutions. C’est pas trop mal perçu. Les freins c’est moi qui les mets parce que je sais que demain sinon c’est l’arrêt de travail… Mon travail peut aggraver mes douleurs de manière certaine maintenant.

L’activité c’est quelque chose d’important dans la famille. Vivement soutenue par mon mari, avec la prise de poids en plus j’en parle même pas ! Largement conseillé d’y aller. A priori dans ma vie de famille il n’y a à priori pas de freins on continue à tout faire. Les épisodes ou j’ai eu très mal par rapport au lumbago, j’ai évité au enfants d’être présent parce que c’était très vif. Après (hésitations) on va dire que voilà je prends des moments de repos, je les impose un peu à tout le monde dans la journée j’ai pas le choix. Ça fait parti de notre quotidien. Même si la position couché j’y suis très mal. Il y a une position sur le coté mais après je ressent une douleur dans la hanche.

Mon médecin, mon rhumatologue, les conseille de l’infirmière sur le site du travail. Le médecin du travail je dirais que oui et non parce que les premier temps quand je lui disais que j’avais mal c’était un peu banalisé, la dernière fois il a moins banalisé parce que j’ai un peu hurlé ! en disant qu’il fallait faire quelque chose parce que chez nous c’est très important (dans notre travail)

Ostéopathe, le kiné pour le dos jamais.

Entretien n°6

La lombalgie c’est des douleurs en bas du dos et des fois de la zone L5S1 là ou il y a le nerf sciatique autour de cette zone. Ma douleur quand elle se réveille c’est que ça doit toucher le nerf sciatique.

Ce qui aggrave c’est la façon de porter les charges lourdes surtout, la façon de porter les charges lourdes, je pense que c’est ce qui provoque les douleurs.

Non je ne vois pas, parce que l’activité physique je continue, même à petite dose mais je continue…

Non je ne pense pas, je me vois pas rester allongé, je ne pense pas.

Ce qui me soulage se sont des étirements, et puis quelques traitements comme le Voltarène, ça me soulage.

Oui après le déjeuner je vais m’allonger.

Oui dans ma tête, je pense oui, je je suis pas médecin, quand la douleur se réveille je pense que c’est parce que j’ai du faire quelque chose, ça doit s’aggraver.

Il y a des choses que je ne fais plus parce que je me souviens avoir eu mal en les faisant

L’activité physique c’est il faut bouger, toujours être en mouvement, aller dans le jardin, faire enfin je ne sais pas, toujours être en mouvement, des activités de la vie quotidienne quoi.

Oui c’est important oui, l’activité physique peut améliorer la santé, et je ne pense pas que cela puisse être néfaste

Je fais moins d’activité à cause parfois les douleurs, mais je vais toujours faire quelques choses, même quand j’ai mal faut toujours que je fasse quelque chose, je peux pas rester à rien faire et puis dire « ça va passer », faut toujours que je sois en mouvement, voila.

Je ne freine pas vraiment mes activités, (rires), pourtant ma femme elle me dit doucement etc, tu vas te faire mal au dos.

Ma femme elle essaye de me freinez un peu parce que je suis toujours en train de faire quelque chose, elle me dit d’arrêter, d’en profiter que je sois en arrêt pour me reposer, mais je peux pas, je fais le ménage je passe l’aspirateur…

C’est quand je vais me poser sur le canapé que je vais le plus le sentir.

Actuellement ça a un retentissement important sur mon travail oui, parce que je suis magasinier et que soulève souvent des charges lourdes, et peut être que je les porte pas … je fais peut être pas attention en les prenant ou je sais pas.

Avant je faisais de l’activité physique et tout, et c’est une fois que j’ai commencé en temps que magasinier que ça m’a déclenché ma lombalgie. Je savais pas ce que ça voulais dire avant les lombalgies, les problèmes de dos je ne connaissais pas, c’est depuis que je fais ce métier.

Peut être qu’avant j’avais déjà ce problème et que ça l’a réveillé, je sais pas.

Non, moi personne n’est venu me donner de conseil, et puis je suis pas bien placé pour en donner

En rééducation j’ai appris des des trucs que je savais déjà, des étirements, bien s’étirer le soir, comment se tenir aussi bien, en sachant que je connaissais déjà, mais j’ai réappris à les faire correctement.

J’avais déjà travaillé ça avec un kiné, même dans une salle de sport en faisant des étirements avec quelqu’un qui s’y connait, un prof de sport, mais parfois on le fait comme on le sent, c’est pas forcement bien.

Entretien n°7

Comme je vous disais ça a commencé depuis 2 ans. Donc avant c’était vraiment très éloigné les douleurs, c’était par exemple 2 fois par an, et c’est venu après plus rapproché, et ces 2 dernières années ou la c’était quasiment devenu 3 à 4 fois par an et la il fallait que je m’arrête assez régulièrement. Et la depuis le mois de mai de cette année ça m’a pas lâché.

Moi je suis parti au départ en me disant que c’était à cause de mon travail, le fait de soulever de la charge en continu du matin jusqu’au soir, je pense que pour moi ça vient de la, après je sais pas.

Surtout le travail en fait…

Moi je pense, oui parce que avant je n’avais jamais eu de problème de dos

C’est une gène en permanence, voilà. A cause de ça j’ai perdu mon boulot, c’est pas facile à vivre.

Ca évolue en mal, ca me fait de plus en mal, ca me gène sur ma vie sur le fait que je peux faire de moins en moins de chose par rapport à mon enfant, j’aime bien bricoler mais je ne peux pas trop me baisser…Je pense que même psychologiquement… on en parlait avec la psychologue hier…je dois la revoir, c’est une chaine.

Maintenant j’ose même plus rien faire, j’ose même plus sortir parce que je sais très bien qu’au bout de 50 mètres ca va commencer à me faire mal au dos. Donc le week end je peux me poser et je sors pas quoi. Même déjà de prendre la voiture au bout d’une demi heure ça me fait mal au dos, de faire les magasins c’est pareil, enfin ça me gène tout le temps, tout le temps, tout le temps… Donc on essai de faire le moins de chose possible.

A force on devient irritable aussi, des fois on en parle avec ma femme. Pas trop avec mon entourage, ca les regarde pas c’est ma vie, les autres ils le prennent plus à la rigolade, voilà moi je sais ce que j’ai. Des fois le fait de faire du sport avec les collègues ça me manque, des fois ils vont faire du tennis, du ski, et bien moi je ne peux pas ! De toute façon je ne peux pas courir, quand je cours le fait de sauter, ce qui me freine c’est la douleur tout simplement, parce que je sais très bien qu’elles vont arriver. Parce que je serais motivé pour le faire mais je sais très bien ce qui va se passer donc je ne le fais pas.

J’ai une réelle appréhension de la douleur, je ne vais pas aller avec eux parce que je sais très bien qu’au bout de 5 minutes je vais abandonné, je vais dire c’est bon j’arrête j’en peux plus, et après ça m’irrite. Après le moral il est un peu à plat.

Depuis que j’ai perdu mon travail, c’est pire parce que je ne vois plus personne, je reste à m’occuper de mon fils. A la maison j’essai d’en faire un peu quand même je vais pas tout laisser à ma femme, mais il y a des choses comme l’aspirateur rien que le fait de faire des gestes ça me fait mal au bout d’un moment.

Pour moi l’activité physique c’est une horreur (rire), je vois ici j’en fais un peu mais faut venir et faut vraiment être motivé parce que la douleur je l’ai des le début, le soir je prend un bain bien chaud pour décontracter les muscles, enfin c’est pas une partie de plaisir, je le vis pas

bien. Je viens en rééducation parce que je me dis qu'il faut que je le fasse, l'équipe est super ils sont toujours en train de nous forcer à faire des exercices mais faut vraiment être motivé. A peine je commence à faire un mouvement à peine je commence à avoir les douleurs donc c'est pour ça aussi que j'avais demandé à changer mes traitements.

Ici on me dit de faire du sport qu'il faut muscler la partie lombaire, et que après il faut que je m'entretienne quand je partirai de la. Faut prendre soin de soi quoi.

Mais bon pour l'instant le sport je le met un peu de coté c'est vrai ! Mais il va falloir des que je sorte d'ici je fasse du sport. Soit je fais de la musculation pour me remuscler mais il faudrait que je fasse autre chose à coté, c'est vrai que j'aime bien la natation, mais essayer de nager la brasse ça me fait mal au dos, le crawl faudrait que je le fasse sur le dos et je ne suis pas un as de la natation, et tout les autres sports ou il faut courir avec des sursauts je ne peux pas. Pas pour le moment en tout cas.

Et je ne suis pas du tout motivé pour sortir, j'ai pas envie de voir du monde.

Je me dis qu'il n'y a pas que le physique qui fait que j'ai mal au dos en fait, parce que des que je suis contrarié ou que je suis énervé je sens tout de suite mon dos se contracté et je sens tout de suite qu'il y a une barre dans le bas du dos, il faut même quasiment que je prenne un cachet derrière ; j'ai l'impression que ca fait comme un fil conducteur que ca vient tout en bas. Donc des fois je me dis la douleur elle est vraiment dans la tête, des fois il faut que je change de cachet, on me dit que c'est toujours la même chose mais pour moi si je prend pas celui la on je me dis que ca ne marche pas ! Même quand on me disait qu'après 2 ans d'examen on ne me trouvait rien, j'ai vu des médecins, des kinés des ostéo, on m'a dit je vais vous soigner, vous aurez plus mal et au bout de 2 jours j'avais toujours mal, je me suis un moment non mais c'est dans ta tête, franchement je pensais que c'était psychologique la douleur. Et c'est ca qui m'énerve c'est qu'on ne me trouve rien. On sait que je suis lombalgique, mais d'ou ca vient exactement ca on le sait pas et ça ça m'énerve. A part savoir qu'il n'y a rien à faire, même une simple opération pour faire partir cette douleur… mais on me dit que non. C'est stressant parce que du coup j'ai envie de faire des choses comme un métier par exemple, mais il y a pleins de choses qui me sont interdites, soit par la médecine du travail, soit moi, si je veux faire un métier ou il y a de la route et bien je ne peux pas. La aujourd'hui j'ai eu la confirmation de votre collègue que je ne pouvais pas être aide soignant, et moi je peux pas être dans un bureau toute ma vie, moi j'ai envie d'etre au contact des gens de communiquer d'etre dans le social, d'aider quelqu'un, pas être derrière une machine, de faire quelque chose qui a un sens a ma vie, ca me plairait.

J'ai vu ma mère qui a fait une tentative de suicide, et je me suis dis que c'était peut être un peu de ma faute parce que j'ai pas eu le temps de parler. Et maintenant j'ai envie de faire des choses qui me rendent utiles. J'ai envie d'être au service de la personne, mais il y a beaucoup de boulot qui sont des choses physiques et ca je peux pas et ca m'énerve a un point des fois je peux pas en dormir la nuit.. J'en ai parlé au docteur V. qui m'a dit que pour lui valait mieux faire autre chose et j'ai fait la connaissance d'un patient hier, et qui était aide soignant pendant

10 ans et qui est obligé de changer, il est lombalgique chronique aussi et il est obligé de changer de métier.

Ce dos ça me gâche ma vie quoi.

Psychologiquement, physiquement, tout !

La médecine du travail m'a fait perdre mon boulot, je sais qu'ils ont fait ça pour moi, mais maintenant j'ai l'impression que tout ce qui me fait envie ils vont me dire non.

J'aimerais bien refaire du sport ça me manque, j'ai fait de la muscu pendant 3 ans ca me faisait du bien, de la natation, un peu de tennis. Ca fait plus d'un an et demi maintenant que je fais plus rien, ça me manque. Je sors plus de chez moi je reste qu'avec ma femme et mon fils, point de vu social il y a mieux quoi !

Ma femme elle comprend et elle essai de me soutenir, après peut être que je m'ouvre pas assez, mais j'ai envie de m'en sortir par moi même, de faire bouger les choses.

Je sais pas comment ça va évoluer, je focalise tout par rapport à mon dos, si mon dos ça va mieux je pourrais peut être faire plus de choses. Si ça va pas mieux, ça va empirer. Je vais essayer de faire du sport renforcer le dos, moi je sais pas si ça suffira. Entre temps j'ai vu un neurochirurgien, qui m'a confirmé qu'il fallait que je fasse ce stage ici, il m'a dit si ça va pas mieux de retourner le voir pour faire des infiltrations mais bon j'ai pleins de copains qui en ont fait et au bout d'une semaine ça revient donc je vais pas m'amuser à en faire toutes les semaines. Moi de toute façon on veut pas m'opérer, enfin on veut pas, il y a rien à faire, je serais obliger de vivre avec.

Je sais pas comment je serais dans quelques annnées…Même pour jouer avec mon petit je peux pas ça me manque.

Mon petit il me dit « papa bobo » je sais pas ce que je vais lui laisser comme représentation, ça me touche.

Entretien n°8

Ca a débuté au départ en 2006, j'avais fait un genre de lumbago, on m'a donné un traitement anti inflammatoire, antalgique, ça a fait un épisode sur 3 semaines puis ça a passé. C'est revenu 2-3 mois après, donc la rebelote cachet plus après examen complémentaire radio et scanner. Donc la on a vu qu'il y avait des protrusions au départ, après on est resté avec kiné et antalgique un moment. 3 semaines après ça m'a fait un épisode j'avais des pertes de sensation dans les mollets. Re scanner de contrôle, il n'y avait pas eu d'évolution flagrante puis c'est resté comme ça. C'est passé j'avais fait presque 50 ou 60 séances de kiné et enfin de compte ça s'est amélioré jusqu'en 2007, ou j'ai refait un autre épisode qui a duré 5 semaines et la on a refait un scanner je crois, ça avait un tout petit peu évolué mais pas énormément. Et la en juin 2008 pareil , donc scanner et j'ai été dirigé vers un rhumatologue, il m'a precrit le traitement que j'ai en cours plus une IRM, donc la on a bien distingué les 2 hernies, puis j'ai revu le

docteur M. qui m’a envoyé faire de la kiné de réadaptation en me dirigeant vers un autre médecin, c’est pour ça que maintenant je suis hospitalisé pour ça. J’ai fait des exercices en kiné de réadaptation mais bon, il n’y pas vraiment d’amélioration au niveau douleur quoi. Il y a plus de souplesse c’est sur mais la douleur est toujours la.

Et la cause…

J’ai fais ma maison de A à Z au départ qu’on a attaqué en 2004, est ce que c’est ça ou pas qui a finit d’arrangé mon dos, je sais pas . C’est pareil je suis fils d’agriculteur et comme j’ai commencé à travailler petit avec mon frère, ça n’a pas aidé aussi. Forcer petit ça n’a pas été bon, ça a accentué le mal de dos.

Et vous faites quoi quand vous avez des épisodes douloureux ?

C’est les cachets à fond pour essayer de le faire passer et la kiné.

Plutot actif ?

Dans la limite du supportable, beaucoup de massage antalgique et après de la mobilisation pour apporter de la souplesse au dos pour qu’on supporte mieux. Et du repos oui parce qu’on est en arrêt de travail pendant cette période la.

C’est quoi pour vous le repos ?

C’est laissé le dos tranquille ne pas le faire forcer plus.

Et à la maison….vous faites quoi ?

Pas grand chose, au début de la crise on est vachement limité par les mouvement, les déplacements, les … quand on est courbé en permanence que ça fait mal ça tire partout, c’est pas agréable. Il y a un retentissement forcément sur la vie de famille et les enfants, on peut pas les porter. Ma femme travaille la journée donc j’essaie de m’occuper tant bien que mal. Il y a des jours ou je fais un peu le ménage un peu la cuisine un peu tout mais c’est pas le plus facile.

Et le sport l’activité physique ?

Disons que j'ai jamais été habitué petit à en faire, j'étais plus souvent dans les champs en train de travailler plutôt que d'aller faire du foot avec les copains.

L'activité physique c'est quoi pour vous ?

C'est faire du sport !

Et le travail dans les champs…

Maintenant le travail dans les champs ça a vachement évolué, c'est beaucoup plus mécanisé qu'avant en 20 ans ça a fait beaucoup de progrès, ça sollicite beaucoup moins le dos. Du coup le sport ben non, je faisais une autre activité physique (travail d'agriculteur) qui ne musclait pas le dos au contraire qui l'esquintait, rester courber c'est pas le plus facile quoi… (Silence)

Le sport m' a jamais attiré puis que j'ai pas été habitué petit, c'est pas rentrer dans mon train de vie, pour moi ça a plus été le travail. Pour moi le sport c'est un loisir, c'est pas obligatoire.

Vous pensez que ça peut avoir un effet sur la santé ?

N'importe comment le fait de s'entretenir ne peut pas faire de mal !

Et sur le dos ?

C'est toujours pareil après quand on mal on hésite, on est vachement restreint pour faire les choses aussi bien pour faire du sport que pour le travail. Il y a beaucoup de choses qu'on fait en serrant les dents quoi. Il y a un moment ou ça marche plus.

Dans le travail qu'est ce que ça a modifié ?

Temps que l'on peut supporter ça va puis à un moment donné quand on arrive plus à supporter ça craque…

Moralement ?

Non physiquement, au mois de juin quand je me suis arrêté je n'arrivais plus à marcher, c'était vraiment… Pendant la grosse crise j'ai fais ce que j'ai pu puis le jour ou ça a lâché je n'ai plus pu et j'en suis la.

Et sur le moral ?

Le moral ça va dans l’ensemble.

Il n’y a aucun moment ou…

Non (sourire) heureusement !

Et votre entourage…

Ils me disent de me faire soigner au maximum, d’essayer de revenir comme avant, il n’y a pas d’influence sur nos habitudes. A part le patron qui aimerait bien que je sois déjà au boulot mais bon on fait pas toujours comme on veut.

Mais vous avez adapté votre travail ?

La je suis toujours en arrêt, mais il n’y a pas de possibilité d’adaptation, mon poste il y a que moi qui le fait, après les autres c’est que des postes assis courbés. J’ai pas le choix faut que ça revienne normal pour pouvoir rebosser.

Et comment voyez vous l’évolution ?

Pour le moment l’évolution à part avoir gagné de la souplesse c’est pas ça sur les douleurs, c’est toujours pareil, et j’aimerai bien qu’ils trouvent quelque chose pour que ça me calme parce que j’aimerai bien ne pas bouffer des cachets le restant de ma vie non plus !

Et qu’est ce qu’on vous a dit ?

Pour le moment pas grand chose… les médecins doivent se revoir, je vais peut être faire une semaine de plus de kiné de réadaptation mais je ne sais pas à quelle sauce ils vont me manger !

Et vous qu’est ce que vous savez sur la lombalgie ?

Pas grand chose du coup, je sais que ça me fais mal, et j’aimerai ne plus avoir mal, c’est une douleur qui est continue, on s’habitue à la douleur c’est sur mais jusqu'à un certain point. Quand je suis en crise je préférai avoir un doigt de pied cassé, ça fait moins mal que ça !

Et vous vous projeté comment dans l'avenir ?

Que ça revienne normal pour recommencer à travailler comme avant (rires).

Objectif travail…

Ah oui parce que ça manque quand même, et puis je manque à mes collègues, certaines machines je suis le seul à les régler.

Et pendant vos arrêt…

Je fais de la kiné pas mal, je m'occupe des gamins, et puis j'essaie de sortir marcher, de pas rester enfermé non plus quoi, pour essayer de me détendre mais faut pas que ça aggrave non plus. J'essaye quand meme de bouger je reste pas immobile.

Mais de trop forcer ça fait plus mal, c'est pas la solution. Tant que c'est pas remis et ranger comme y faut pas forcer dessus je pense.

Et quand vous marcher

Je vais autour de chez moi il y pas mal de chemins, me balader un peu, à mon rythme, pas en marche soutenue, mais dans la limite du possible.

Et qu'est ce qui vous limite surtout ?

Plus j'avance plus ça tire et plus je fais des poses.

Et avec votre frère vous en discuter ?

Non

Mais je pense qu'on a forcé trop jeune et que notre dos a vieillit trop vite, que l'on était pas assez formé et qu'à la suite on en paye les pots cassés.

Et quand vous aller sortir d'ici qu'est ce que vous allez faire ?

Je vais déjà voir ce qu’ils vont me dire, pour la douleur pour le moment ils ont rien trouvés. Peut être qu’ils vont me proposé de continuer la kiné ici, peut être qu’il vont me faire une infiltration je sais pas.

Et sur l’activité physique en générale qu’est ce qu’ils vous disent ?

Que j’ai gagné beaucoup de souplesse, que c’est beaucoup plus fluide qu’au début, le problème c’est que la douleur est toujours la, ce que j’arrive à faire je le fais mais en fin de soirée si j’ai fais du sport ça tire.

Et dans la vie de tous les jours…

C’est des promenades moins longues des trajets en voiture moins longs, position assise moins longtemps, il n’y a pas raiment de position neutre

Et psychologiquement ?

Ca va, je le prend comme ça vient, que j’aille bien ou pas je n’y changerai rien !

Entretien n°9

Disons que cette lombalgie elle est beaucoup plus forte depuis 2 ans depuis que j’ai porter une patiente qui n’était pas autonome et assez forte et qui avait été opérée d’une PTH, j’ai du porter la patiente longtemps le temps qu’on lui fasse les soins. A la fin de ces soins il y a le médecin qui a voulu voir le pansement, j’ai reporté la patiente, après ça je n’ai pas réussi à me relever. Suite à ça j’ai fais pas mal d’examen qui n’ont pas montré grand chose à part le canal lombaire étroit et la maladie de sheuermann, et j’ai été mis en arrêt de travail. Ce qui se passe d’un point de vue physiologique, c’est que si je ne fais plus rien et que je reste chez moi à ne rien faire je n’ai pas mal au dos. Par contre des que je porte quelque chose des que je prend ma douche, que je me baisse pour me laver les pieds quand je me rase que je me brosse les dents j’ai mal, ça déclenche immédiatement des douleurs. Il y a des choses qui me sont impossibles comme de rester trop longtemps assis sur un tabouret sans dossier. Ce qui est bizarre c’est que quand je suis dans mon canapé, les jambes allongées sur un pouf, là je ne ressens pas de douleurs. Par contre à Noel j’ai mangé chez ma sœur, j’étais assis une chaise toute bête j’ai cru que je ne finissais pas le repas tellement j’avais mal. Je comprend pas trop, par exemple en ergothérapie je fais des exercice ou je dois lever les bras assez au m’étendre ça déclenche la douleur directement !

L’exercice physique…

Oui mais qui n’est pourtant pas violent, je ne force pas beaucoup, mais ça déclenche la douleur très très vite.

C’est ce qui vous freine…

En fait il n’y a rien qui me freine, le je suis très motivé j’ai 39 ans, j’ai toujours été quelqu’un qui fait plein de chose donc je ne viens pas à reculons, quand je viens j’essaie d’en faire le plus possible mais quand la douleur se déclenche ça me fais perdre le sourire et là c’est dur

Moralement ?

Ca a été dur moralement

C’est à dire ?

Disons que il y a un an de ça quand j’ai été mis sous traitement pour mon HIV et qu’en plus j’ai eu ce problème dorsal qui m’a créer des soucis dans mon emploi vu que je suis aide soignant. Je n’avais pas beaucoup de diplôme donc j’avais trouvé une formation et un travail qui me plaisait auprès de patient. Et de me rendre compte petit à petit que je ne vais plus pouvoir faire ça, ça a été très dur à assumer parce que je me disais que déjà le HIV avait des répercussions sur ma vie sentimentale ou amoureuse et sur ma vie de tous les jours plus le problème de dos. Peut être que je vais perdre mon emploi qu’est ce que je vais faire après, ça a remis beaucoup de choses en questions, c’est vrai que pendant un moment j’ai eu le moral dans les chaussettes. Avec le temps ça va un peu mieux, je ne me laisse pas aller. J’en ai parlé à mon médecin traitant quand un jour c’est allé vraiment trop loin, il m’a dit on va attendre l’hospitalisation au chu, et en fonction des résultats je vous mettrais sous antidépresseur. On en a pas reparlé et en fait temps mieux, parce qu’aujourd’hui je n’ai pas besoin d’antidépresseur ça va beaucoup mieux.

Mais bon quelque part c’est toujours dur de ne pas savoir de quoi sera fait l’avenir par rapport au problème de lombalgie.

L’évolution vous la voyez comment ?

Je pense que je vais souffrir toute ma vie du dos, il y a que des traitements antalgiques puissants qui arrive à me soulager, j’essaie de les réduire car je ne suis pas pour les traitements médicamenteux. D’autant plus qu’à un moment mon médecin m’avait mis sous actiskénan 20 ce qui a des répercussions sur mon état (hésitations), ça me crée des problèmes intestinaux, des problèmes de sommeil et je ne me sens pas bien avec ça.

Le retentissement de votre dos sur mode de vie au quotidien…

Pendant un moment je me suis renfermé sur moi même parce que je ne voulais plus trop sortir, j'avais peur d'avoir mal au dos, je me posais aussi des questions par rapport à mon boulot. Je suis resté assez longtemps enfermé chez moi à ne rien faire.

Pas d'activité ?

Non télé c'était tout ce que je faisais. J'adore cuisiner mais lorsque je cuisine après à la fin de la journée j'ai très mal. Donc ça ça a été aussi une grande déception parce que je me disais que je n'étais plus comme avant.

Pour vous l'activité physique c'est quoi ?

C'est l'activité de tous les jours, ça peut être l'activité sportive, mais moins sur l'emploi, maintenant je sais que comme j'ai mal au dos je me projette pas dans l'avenir sur un

Métier qui fasse forcer. Je me suis justement inscris à un bilan de compétence pour justement essayé de voir ce que je pourrais être amené à faire de différent puisque mes employeur ne me trouve pas de poste adapté à mon état de santé.

Et le sport dans votre vie de tous les jours…

J'ai toujours gardé espoir que l'on arriverait à stopper ce mal de dos, mais pour l'instant du sport j'en fais pas, le seul sport que je fais depuis que je suis en hôpital de jour je fais de l'ergothérapie de la kiné de la piscine c'est tout pour l'instant. Ca me fais du bien je le sens.

Ca a des effets sur votre santé ?

Ca me fait du bien, je sens que faire travailler un peu les jambes, apprendre des techniques pour pouvoir porter des charges ou faire de la manutention ca me fais du bien au corps c'est sur, quand je marche ça me fais du bien de travailler et tout ça mais par contre je ne vois aucune évolution par rapport au dos. Le matin j'attaque par l'ergothérapie, le seul fait de monter les bras et de mettre les billes sur les baguettes ça me déclenche une douleur, il n'y a aucune évolution. J'étais peut être un peu trop optimiste par rapport à ça dans le sens ou venir ici et me remuscler le dos, je pensais que justement j'allais être très remusclé du dos et que ça allait me permettre de ne plus forcer sur ma colonne et de ne plus avoir mal, mais ce n'est pas le cas.

Et vous êtes la depuis combien de temps ?

Ca va faire une semaine et 2 jours, c'est peu aussi.

Qu'est ce qu'on vous dit ?

Qu'il faut que je continu que c'est pas en une semaine que ça va tout changer, quand j'aurai fini que je continuerai surement chez un kiné en externe ou à faire un peu de natation ou de chose comme ça, pour muscler le dos, mais pour l'instant j'en ai pas trop parlé au médecin, je ne veux pas être négatif alors que je suis motivé justement.

Et comment vous l'envisagez l'après ici ?

J'attend de voir la fin, je vais me remettre un peu à la piscine en me remotivant un peu et essayer de prendre le moins d'antalgique possible et de reprendre une vie normale. Je suis inscrit à la MDPH mais je ne vois pas du tout handicapé, je sais qu'il y des personnes beaucoup plus mal en point que moi. Je veux garder une vie normale le plus possible et je ferais tout pour ça

Et le repos…

C'est un moyen pour moi un peu d'oublier la douleur au dos parfois, et puis c'est un moyen de se reposer comme on fait tous les jours. Par contre chez moi c'est peut être pas ce qui a de mieux adapté, parce que je me suis acheté un futon ! Tout le monde m'avait parlé des formidables futons et je trouve que c'est une belle merde, c'est bas et je dors très mal sur un futon ! Autrement je mets une couette entre le drap housse et le matelas pour amortir.

Et comme vous êtes en arrêt de travail comment se déroulent vos journées ?

Entre les médecins et spécialistes que je suis allé voir j'ai été pas mal occupé, le kiné un peu de natation, les démarches que j'entreprends. J'ai beaucoup de problème avec la sécurité sociale aussi parce qu'au bout d'un moment je comprend bien qu'ils ne peuvent pas me payer à rien faire. Donc j'ai vu des assistantes sociales pour essayer de m'en sortir un peu parce que je dois bien vivre comme tout le monde. Au début de mon arrêt de travail je ne faisais rien du tout. Je me reposais, j'en avais peut être besoin aussi. Mais la depuis quelques mois je n'ai pas le temps de m'embêter.

La natation vous en faite déjà ?

J’ai essayé sur les conseils de mon médecin traitant, je ne savais pas que la brasse n’était pas adapté pour les lombalgies donc moi je faisais que de la brasse bien évidemment et j’avais mal au dos et j’ai baissé les bras. Et lui me disait « ça peut vous faire du bien » et moi je disais « mais ça peut me faire du mal aussi ». La j’ai appris certaine chose comme le fait de nager sur le dos ou le crawl ou la brasse coulée, ou je pourrais essayer quand je sortirais.

Dans votre environnement…

J’habite pas loin d’une piscine donc ça il y a pas de soucis.

Et une autre activité ?

La marche je ne suis pas très fan des balades en pleine nature, le vélo je déteste. La natation fait marcher tous les muscles c’est bien.

Et votre entourage ?

C’est un peu complexe parce que je n’ai plus beaucoup de rapport avec ma famille mais mes amis comprennent que j’ai mal au dos, ne savent pas trop quoi en dire parce qu’ils ne sont pas dans le milieu médical, se sont fait du soucis pour moi quand ils ont vu que je ne faisais plus grand chose que j’étais chez moi. Mais je garde quand même mes amis.

J’ai mon frère à paris, on a de bonne relation, il a le même problème. Quand on se voit on a d’autre sujet de conversation que de parler de notre mal de dos mais la dernière fois il m’a dit que quand il rangeait son linge dans une étagère il a été complètement bloqué. Je sais que lui souffre aussi. Pour mon entourage ce sera tout.

Entretien n°10

Parlez moi de votre lombalgie

Ma lombalgie elle est ancienne, pendant 30 ans je me suis relativement bien porté, et récemment l’an dernier j’ai commencé à avoir des douleurs chroniques qui se sont installées et gênante dans mon travail. A savoir quand je suis assis la douleur s’installe et ne part pas et sur une chaise quelque soit son confort et ma position, et surtout quand je suis assis en voiture. Donc je cherche à me débarrasser de cette douleur, à l’atténuer de manière la plus douce possible, c’est à dire sans envisager d’intervention chirurgicale.

Et la cause…

Je ne suis pas médecin mais j’ai une petite idée, et puis on en a parlé. Déjà l’âge, au bout de 30 ans le corps s’est modifié, j’ai de l’arthrose sur L4 L5 S1, j’ai une petite hernie discale et une inflammation sur ces dernières vertèbres, et puis le facteurs déclenchant ça a été la voiture.

Dans le cadre du travail ? Vous faite quoi ?

Je suis journaliste.

Et le retentissement sur votre travail…

J’ai du mal à travailler mais bon je ne me suis pas arrêté. Mais c’est difficile parce que je suis toujours assis dans la voiture pour aller faire mes reportages, ça pose problème et puis après il faut que je sois assis au bureau pour rédiger.

Et vous avez fait des adaptations ?

Un, j’ai changé de fauteuil, j’ai acheté un fauteuil le plus adapté à mon dos, et puis j’ai consulté, j’ai le port d’une ceinture lombaire en permanence, ça facilite l’exercice, et puis j’a consulté surtout.

Et qu’est ce qu’on vous a dit ?

Le Pr C. voulait me mettre un corset plus rigide sur mesure, j’ai pensé que c’était peut être un peu tôt. On a changé de ceinture, j’ai pris une ceinture plus grande qui prends le maximum du dos. Il m’a donné un traitement par Paracetamol et AINS pendant 1 mois. J’ai fais attention à mes déplacements aussi pendant 1 mois. Et puis la douleur s’est relativement atténuée. Ce que le Pr voulait c’est éviter que la douleur s’installe et s’auto entretienne, et effectivement ça a marché. Je l’ai toujours cette douleur mais elle est beaucoup moins vive qu’auparavant, là je suis assis depuis 10 minutes mais c’est supportable alors qu’avant c’était insupportable. J’avais un point exquis près de la crète iliaque sans irradiation dans la jambe, qui s’installait très rapidement, et donc j’étais obligé de me lever de marcher de me mettre à quatre pattes pour faire des étirements.

Vous avez modifiez vos habitudes de vie ?

Je me suis mis à en refaire, car comme je vous disais en préambule, l’an dernier j’ai dernier avant de partir en vacances j’ai eu une douleur similaire que j’ai réussi à faire disparaître en faisant des abdominaux des choses comme ça, tous les matins, et donc j’ai recommencé.

Donc vous avez repris une activité physique…

J’en ai toujours eu une, je fais du basket légèrement dans la semaine, je fais de la randonnée sportive (25km) en dénivelé le week end, du ski de fond, du VTT.

Et l’impact que ça a sur votre dos…

C’est plutôt l’inverse, l’impact de mon dos à sur la randonnée, je pouvais plus faire 25Km. Même une petite balade s’était devenu difficile, quand je me baladais dans Paris, j’essayais de marcher et de ne pas prendre le métro. Même sur du plat, au bout d’un moment j’avais le dos en feu. Donc j’ai arrêté pour le moment la randonnée, le basket, le VTT pas de problème aucune douleur, de toute façon le vélo c’est bon pour le dos.

Vous avez plutôt ralenti

Oui j’ai diminué

Et ce qui vous freine ?

Les freins c’est la douleur, que la douleur, après je suis raide du bas du dos.

Vous avez constaté que ça faisait quoi l’activité physique sur votre dos ?

Pour certaine activité physique je ne peux plus courir (problème de gonarthrose également) donc je ne peux pas. Et puis la marche à un certain rythme, je ne pouvais pas marcher de manière dynamique à cause de la douleur.

Et le retentissement sur votre moral ?

Oui, et bien je m’en suis surtout redu compte quand j’ai relu le questionnaire à l’entrée ici, ou on me disait est ce que vous avez l’impression d’être plus irritable, de mauvais humeur… Et je me suis surpris à cocher plein de cases ou j’étais plutôt de mauvaise humeur. Et puis une situation qui me correspondait bien c’est que j’avais l’impression de ne pas avancer, pas forcément physique mais à tout point de vue. De manquer de dynamisme. Alors après il peut y avoir plein de raison…

Et dans vos rapport avec autrui…

Il faut demander à mon entourage mais je peux être surement plus désagréable que je ne suis oui.

Et le repos, vous y accordez une place différente ?

Je suis quelqu'un qui ne me repose pas beaucoup, ça peut être des séances de relaxation ou je fais des étirements pour mon dos, et puis après c'est le repos le sommeil quoi. Dans une journée pour que je m'allonge faut vraiment que ça aille mal.Ca m'est arrivé il n'y a pas longtemps parce que la douleur était vive, j'ai pris un AINS et puis je me suis allongé une heure. Faut dire que je travaille chez moi. Mais c'est épisodique.

Qu'est ce qu'on vous a dit ici…

Et bien on en a pas trop parlé mais si je suis la c'est pour un renforcement musculaire à tout point de vue. Un des objectifs c'est de pouvoir poser ma ceinture lombaire parce que même si cela soulage, ça amoindrit la tonicité des muscles. Et puis l'activité physique, c'est d'avoir les bonnes pratiques en terme d'activité physique pour que ce soit un remède à la douleur plutôt que l'inverse. Et puis dans un environnement surveillé adapté avec des gens qui vont vous dire les bons exercices pour votre dos et pour votre maintien et qui vont vous dire ce qui ne faut pas faire. Et ce que je viens chercher aussi c'est des exercices que je pourrais refaire chez moi de manière régulière.

Donc vous êtes motivé.

Oui et puis c'est moi qui en ai fait la demande.

Et votre entourage ?

Il pleure (rire) non il y a pire comme situation !

Et l'évolution …

Je vois une évolution progressive, parce que ce n'est pas non plus prendre un médicament et la douleur disparaît, retrouver des habitudes de maintien de travail physique pour que j'ai un renforcement adapté et qui vont aider à corriger les causes de ma douleur .

Entretien n°11

Parlez moi de votre lombalgie…

La lombalgie c'est un mal de dos très intense qui se trouve en bas du dos et qui excusez moi l'expression « qui pourri la vie » même des médicaments, et fait que certains médecins n'arrivent pas à comprendre notre douleur, ne gère pas notre douleur, nous donnent des médicaments alors que la plus part du temps ça nous fait plus de mal qu'autre chose. Pour mon cas c'était comme ça.

Et la cause dans votre cas…

La cause c'est du à un accident du travail, j'ai trop forcé ce jour là et il y a eu un craquement, et après c'était comme un coup de poignard tout le bas du dos, je me suis retrouvé en ayant le bassin complètement coincé, je ne pouvais plus marcher, je ne pouvais plus conduire, je ne pouvais aller faire mes courses, j'étais devenu très douloureuse, que je n'arrivais pas à contrôler, il y a eu beaucoup de médicament qu'on m'a donné, j'ai eu 2 infiltrations qui m'ont plus abimés qu'autre chose. D'ailleurs mon rhumatologue m'a dit « mais pourquoi on vous a donné des infiltrations ? ». Il y a beaucoup de chose qui font qu'on vous empoisonne la vie, alors que le seul remède c'est la rééducation.

Qu'est ce que ça a modifié dans vos activités de tous le jours ?

Je vous dis tout, je ne pouvais même plus lever les bras. Il y a mon fils qui a une petite de 18 mois, je ne pouvais plus la porter, ça fait mal. Quand elle demandait à marcher avec son petit doigt que je ne pouvais plus aller me promener avec elle, je ne pouvais plus faire le ménage, je souffrais, je souffrais, je souffrais…

Ca a duré un an et demi, après j'ai repris 3 semaines et au bout de 3 semaines le dos a re-pété et les douleurs sont revenues encore plus intenses. Ce qui fait qu'après je suis partie 1 mois en vacances : première semaine ça a été, deuxième semaine je me suis sentie mal, troisième semaine, j'avais très mal, bloquée du coup jusqu'en bas du dos, et quatrième semaine je me suis retrouvée avec des inflammations… des inflammations… des inflammations puis arrêt à répétition. Dès que le traitement anti inflammatoire s'arretait dans les deux jours qui suivait ça repartait de plus belle.

Donc ça a retenti sur le travail…

Oui j'avais 6 chantiers à moi en CDI et je me retrouve plus qu'avec 3 chantiers, donc le salaire en baisse, l'incapacité de le faire et puis plus l'énergie de le faire...

Et le moral ?

Le moral dans les baskettes. Beaucoup de personnes n'ont pas compris ce que j'avais. Le regard des proches qui changent, des soit disant amis, qui ne comprenaient pas pourquoi j'étais toujours à la maison, j'avais jamais le moral, je sortais pas, c'était catastrophique.

C'était vue d'une manière c'est simple, quand ça a démarré en 2009 que je suis allée voir mon médecin que je lui ai dit comment je me sentais, que j'étais très fatigué et que j'avais très très mal. Il m'a dit « mais comment ça tu as très très mal ! » il m'a sorti « c'est que tu veux pas aller bosser, c'est que tu as des problèmes dans ton boulot » , Je lui ai dit que non, je m'entendais très bien avec mon chef et mes collègues. Il voulait me dire que je n'étais pas capable d'aller bosser parce que je n'avais pas envie alors que c'était la douleur qui me faisait ne pas aller au boulot. Donc j'ai changé de médecin et entre temps mon ancien médecin m'avait prescrit une rhumatologue sur Riom. La rhumato elle m'a fait passer un scanner, donc effectivement il y a une hernie discale, et puis après une IRM et elle m'a dit je ne sais pas lire l'IRM. Elle m'a donné des AINS et prévue des infiltrations. Ca duré comme ça pendant un labs de temps.

Mon mari est atteint de la SEP, il a demandé au neurologue vers qui je pourrais m'orienter. Le Pr C a regarder l'IRM et a dit qu'il y avait un pincement de la moelle épinière et a dit surtout prenez tout de suite rendez vous en rhumato. En l'espace d'une demi heure j'en ai appris beaucoup plus qu'avec mon ancien rhumatologue.

Et après on m'a parlé de la rééducation.

Et l'activité physique…

L'activité physique j'en faisais pas parce que j'ai un métier qui est très physique. Je suis dans une entreprise de nettoyage, on a du matériel très lourd à porter des positions pas très adaptées, on se fait plus de mal qu'autre chose. On a d'énormes poubelles à vider, c'est hallucinant. On porte des seaux de 5 litres, faut les vider, puis les remplir, faut se baisser, toutes les mauvaises positions qu'on a pu prendre. C'est la justement ou ca a péter. C'est très physique, on a des aspirateurs de 5 Kg, c'est lourd, c'est contraignant c'est très physique. Donc ça le sport y en avait !

Et donc à l'extérieur ?

Non, c'était plutôt repos. Télévision, canapé, mes courses, aller flâner, tout ce que je faisais plus quoi.

La marche ?

J'allais régulièrement au marché le samedi, j'y suis pas retourné parce que je marchais plus. Depuis que je suis la il y a une motivation à refaire du sport. C'est une énergie, un bien être.

Ca m'a apporté énormément. Le week end dernier, j'ai fait ma petite heure de gym le matin, l'après midi je me suis reposé, dimanche matin j'ai fais mon sport

Pendant une heure, l'après midi j'ai voulu aller à la piscine mais elle était fermée donc on a été marcher pendant 1H dans les rues. Ca m'a fait un bien être, après j'avais pas de douleur j'étais bien, j'étais apaisé.

Vous pensez que l'activité physique a un effet ?

Il y a un effet, il y a une poussée d'adrenaline, il y a une énergie qui devient débordante. Je me suis même abonnée à la piscine, toute les fin de semaine je vais y aller. C'est motivant, c'est des choses que je n'avais jamais fait toute seule. Des abdominaux j'aurais jamais pensé en faire, je fais du vélo, je fais du rameur, je fais des pompes… Je ne le faisais pas parce que j'avais peur de me faire mal. Au début j'ai montré comment je faisais des pompes, on m'a dit arrêté la parce que les positions ne sont pas bonnes et ne sont pas adapter. J'avais même peur de monter les escaliers à cause de la douleur.

Comment voyez vous l'évolution ?

Positif, si je continu comme ça je me vois bien dans 10 ans, et le moral est au beau fixe, je progresse de jours en jours tout en ayant quelqu'un à coté de moi qui me montre que je peux aller plus loin. Il y a que pour le travail, pour l'instant je sais pas, la je suis en arrêt jusqu'au 29 après normalement je vais être prolongé d'une semaine supplémentaire.

Et après vous vous sentez de re attaquer ?

Non après j'aimerais bien avoir quelques jours, parce que la c'est très très dure. Il y a un bon résultat mais il y a quand même un épuisement et puis même si il y a une énergie votre mental est quand même fatigué.

Et vous allez refaire le même travail ?

Oui j'ai 3 chantiers en attente, je vais les refaire tout en adaptant ce que j'ai appris ici, ceux la ne vont me poser aucun problème. L'avantage que j'ai c'est que je peux modifier mon emploi du temps.

Depuis une semaine je fais de la sophrologie une fois par semaine, comme je suis très stressé et que j'ai beaucoup de problème familiaux, ça ça m'apporte un bien être également et je continuerai chez moi.

Entretien n°12

Parlez moi de votre lombalgie…

Ma lombalgie, elle me prend la plus part du temps au rein, et j’ai mal au milieu c’est atroce, ça me donne même mal au sternum et les cervicales. J’ai une hernie qui coule à droite. Ca fait 30 ans que j’ai mal au dos. Au tout début j’avais 11 ans j’ai sauté pieds joints d’un banc et je suis retombé jambes tendues, la chose à pas faire, et la ça m’a tout détraqué. Et puis à l’époque on allait voir le rebouteux. A 16 ans j’ai commencé à travailler j’ai fais de la charpente, on portait tout sur l’épaule, et à partir de la ça s’est détraqué sans cesse.

Et l’épisode actuel…

Depuis décembre j’ai fait une rechute, je rentrais de vacances, et j’étais à la maison, et la je suis allé voir le Dr R et c’est lui qui m’a dit que il fallait venir la.

En période douloureuse que faite vous ?

Je prends du Myolastan et de l’efferalgan. Myolastan j’en prend pas souvent et Efferalgan j’en prend très souvent, parce que toutes les nuits je m’endors vers 23H et à 5H du matin je suis debout, c’est une horreur.

Et concernant votre activité…

En été je suis à la piscine et l’hiver je suis au service technique alors tout dépend ce que l’on me fais faire. L’année dernière j’ai posé du carrelage pendant 2 mois, à quatre pattes pendant 2 mois à la fin je n’en pouvais plus, j’étais vraiment à bout. Cette année il y a encore du carrelage à faire je leur ai dit que je ne pouvais pas. Ils m’ont bien dit que ils ne m’y mettraient pas mais…

Il y a un retentissement sur le travail ?

Ah oui oui oui c’est certain, suivant les positions qu’on fait.

Et dans vos habitudes au quotidiens…

Rien que le fait de porter des charges de me baisser de m’asseoir ou le reste… Après je marche au travail, je suis obligé de marcher vite mais je ne peux pas le faire trop longtemps.

J'aime bien la marche, je fais beaucoup de marche mais il faut que je marche à mon allure, si il faut marcher vite je ne peux pas.

Et vous faite de la randonnée ?

Je marche tout le temps mais pas en randonnée, je pars tout seul ou avec ma femme et on part tous les jours pendant 1H 1H30, c'est mon plaisir.

Et il y a des choses qui freinent votre activité physique ?

Mon mal de dos des fois mais la plus part du temps il faut que je marche. Et je dirais même que en marchant doucement ça me détend.

Et l'activité physique a un impact sur la santé ?

Ben moi la seule activité physique que je fais c'est la marche, après faire un sport, je pense que si c'est mal fait, je pense que ça a quelque chose à voir. C'est pour ça qu'aller faire du sport tout seul sans quelqu'un qui me conseille je ne peux pas. Je ne le ferais pas, parce que j'aurais peur de faire mal et d'avoir mal au dos après. Bon les exercices que j'ai appris ici je les ferais c'est sur mais après je n'en ferais pas d'autre, j'ai trop mal au dos pour.

Et l'activité sur le mal de dos…

Ca a un effet, tout de suite oui, ça m'apporte du bien être. Il y a qu'une chose que je ne comprend pas trop avant ça me brulait que à droite et maintenant que je suis ici ça me brule tout le long. Est ce que c'est bien, est ce que c'est pas bien je ne sais pas il y a que le médecin qui… Pour ma part je pense que 3 semaines ici comme ça c'est trop intense, moi ce serait plutôt un suivi d'un jour par semaine - parce que ça empêcherait pas les gens de travailler - sur 6 mois. Je pense que ce serait mieux, parce que la c'est 3 semaines intenses, déjà au bout de la première j'étais claqué, maintenant ça va a peut près mais ça dépend des jours. Des que l'on force trop, le lendemain les jambes nous brulent tellement que je n'arrive pas à bien faire. Avec de la récupération et un suivi ce serait mieux.

Et sur le moral…

Ca va un peu mieux mais ce n'est pas… En fin de première semaine, je me disait ce n'est pas possible, ça force vraiment trop. Et avant j'étais démoralisé totalement, ça c'est vraiment quelque chose, en fait on ne sait pas ce que l'on a, on a mal on ne sait pas pourquoi. Toutes

les nuits impossible de dormir, j’empêche ma femme de dormir aussi, une anxiété qui s’installe aussi…

Et sur votre entourage

Et bien moi je réveille ma femme toutes les nuits, moi j’arrive pas à dormir elle non plus, il y en a déjà un de malade ça suffit.

Et vos motivations ?

Mes motivations c’est de reprendre le travail, de toute manière il n’y a pas d’autre solution il faut aller au travail. Les gens à l’extérieur ne comprennent pas que l’on est mal au dos, on m’a déjà demandé si les vacances se passaient bien ! La personne qui n’a pas mal au dos ne comprend pas il faut avoir mal au dos pour comprendre ce que c’est que le mal de dos. Je suis content pour les gens qui n’ont pas mal au dos parce que quand on a mal au dos c’est vraiment invivable. Il y a des jours ou je n’arrive plus à me tenir debout, un coup je m’assoit un coup je suis debout et quand je suis au travail je ne peux pas faire autrement je suis obligé de travailler e de faire ce qu’on me dit de faire même si il y des choses que je devrais pas faire. Et puis moi je suis à la ville il y a tellement de malade la bas que les postes aménagés il y en plus.

Et l’évolution…

Moi je cherche un autre emploi à l’entretien ailleurs, malheureusement les places elles sont chères.

Pour mon mal de dos honnêtement je crois pas que ça s’arrange bien, à moins qu’il est un miracle mais je n’y crois pas.

Et quand je vais sortir je vais continuer la marche et les exercices.

Et qu’est ce que qui pourrait vous freinez à faire de l’activité physique ?

Ce qui pourrait me freinez à poursuivre les exercices c’est peut être d’être dégouté de ne pas trouver un autre emploi, pour moi mon but c’est de trouver un emploi ou je suis content d’aller au boulot le matin, ça doit être votre cas je pense et je crois la moitié de la journée doit être faite.

SILENCE

Entretien n°13

Parlez moi de votre lombalgie, racontez moi votre mal de dos…

C’est par épisode, il va y avoir des périodes ou ça va bien, et il y d’autre période il va me suffire de me mettre sur le plan de travail d’éplucher 3 légumes et d’avoir la barre, une barre qui est insoutenable à en pleurer, ou le fait de repasser par exemple, des petites choses comme ça, mais c’est pas obligé que ce soit longtemps. Meme quand je suis assise à éplucher des légumes l’été ça peut me le faire. Alors des fois la barre est en haut, des fois elle est en bas mais c’est toujours sous forme de barre. La je vais chez le kiné et ca me fait du bien parce que la ça fait 2 mois que je n’est pas mal.

Et les facteurs déclenchants…

Je ne sais pas, je suis très stressée, ça joue peut être aussi, je fais souvent des sciatiques

Je suis une personne très anxieuse, les migraines ont un rapport aussi avec l’anxiété, un magnétiseur est en train de chercher d’ou ça provient. Je suis trop émotive je perçois trop les choses. Maintenant je suis à la maison, avant quand je travaillais ça pouvait se comprendre aussi le fait de rester debout, de porter des charges, mais maintenant, j’ai rien qui m’oblige à faire des choses à part faire la cuisine, mais ça tous le monde le fait ! (rire)

Les gestes de la vie quotidienne ont un retentissement sur votre mal de dos ?

Les mouvements …

Non il vaut mieux que je me repose quand j’ai mal, c’est plus une activité qui va me le déclencher, et le repos me soulage

Qu’est ce que vous entendez par repos ?

Allongé ou là coincé avec des coussins sans bouger.

Et l’activité physique…

Si quand on va marcher ça me fait du bien, ça oxygene l’esprit, puis on a besoin de sortir…

Et sur votre dos ?

L’activité physique sur mon dos ça ne fait rien de particulier, c’est plus sur le moral, l’état général, mais sur le dos ça n’a aucune influence pas pire pas mieux

Et sur la santé en générale ?

Oui Je vois les périodes ou je vais marcher je me sens mieux. Mais bon je suis entourée de gens qui travaillent et je ne veux pas aller marcher toute seule, j’ai quand meme des amis qui sont disponibles et avec qui je fais des choses et puis avec mon mari le week end on part faire un tour.

Il me faut une motivation mais je ne suis pas sportive, loin de la !!! j’ai déjà fais des tentatives mais ca n’a jamais marcher longtemps. Pas en groupe je préfère avec une copine, la gym tout ça ça ne me tente pas du tout…J’ai essayé mais au bout de quelques mois j’ai arrêté parce que ça me gonflait ! Je ne dis pas que ce n’est pas bénéfique sur le corps, mais il faut avoir cette petite envie d’y aller que je n’ai pas.

Et la kiné ?

La kiné par contre c’est une demi heure de massage, elle me masse pendant une demi heure le dos, ça me relaxe, ça me fais du bien, en plus cette kiné la j’ai la chance qu’elle soit ostéopathe, elle manipule, ce qui est un bon point parce que quand je vais la voir pour le dos elle regarde si il n’y a pas un dysfonctionnement et évidemment elle a trouvé. Et puis je suis allée la voir 15 jours avant pour la sciatique. Il y a bien une coïncidence aussi avec le dos.

Après c’est massage allongé, c’est relaxant, c’est une demi heure ou on décompresse en fin de compte.

Le fait est que ça va faire 2 mois que je vais la voir et depuis je n’ai pas eu de nouvelle crise, alors qu’avant des que j’allais au dessus de l’évier j’avais la barre.

Et des étirements ?

Non massage massage, je l’ai vu il y a 2 ans et ça n’avait été que massage, mais moi je trouve que c’est bien parce que dans mon entourage je connais tellement de gens qui ont des kiné qui les laissent seuls travailler et qu’en fin de compte qui ne savent pas si ils font bien leurs gestes ou pas.

Et dans votre entourage d’autre personne souffre du mal de dos ?

Il y a mon mari, vous pouvez le questionner (rire)

Lui il est très têtu, il ne fait pas grand chose en fin de compte la semaine derrière il s'est bloqué en taillant la haie, je lui est bien dit tu prends des cachets, mais il n'en veux pas.

Et qu'est ce qu'il vous conseille ?

Pas grand chose… Les cachets j'en prend déjà assez pour la migraine !

Et pendant vos périodes très douloureuses …

Ca va être par exemple suite à un effort, suite à une posture peut être, le fait de rester debout immobile.

Et qu'est ce qui retentit sur votre moral ?

C'est plutôt les migraines, finalement le dos c'est secondaire par rapport au migraine, et puis le dos c'est épisodique, les migraines c'est journalier.

Et l'évolution pour votre dos vous la voyer comment ?

Je pense que ça ne va pas évoluer, je pense que je vais continuer a avoir des crises épisodiques comme ça, enfin j'espère que ça ne va pas évoluer. Peut être que ça vient du fait que je ne suis pas assez sportive et pas assez musclée aussi, donc des que je force un peu, je ne sais pas, je verrai…J'y pense pas, je vis au jour le jour, tout de suite je vais chez la kiné ca me fais du bien, puis on arrêtera puis ça reviendra….

Et sur votre travail ?

C'est autre chose qui a retentit sur le travail, c'est la migraine et la dépression.

C'est sur que quand on avait une palette de papeterie à mettre en rayon le soir on rentrait on était cassé. Ca s'arrêtait la, j'ai jamais eu d'arrêt de travail pour mon dos ? Je supportais. Des petits arrêts de travail j'en ai jamais eu en fait.

Vos freins à la pratique de l'activité physique ?

De la marche si je n'en fais pas c'est que je n'ai personne pour aller le faire avec moi, parce que sinon aller marcher 3 fois par semaine il y a des périodes ou on arrive à le faire. J'ai une amie qui est infirmière et qui a des semaines de repos. Mais sinon les activités physique c'est

que je n’aime pas ça. J’ai des copines qui vont à la piscine qui font des activités, je pourrais les accompagné mais ça ne me plait pas tout simplement.

J’irai à la piscine je payerai l’entrée, je ferais un aller retour et puis basta !

Entretien n°14

Parle moi de ton mal de dos

Pour moi c’est du à un accident de voiture que j’ai eu il y a 2 ans, depuis ce moment sur le coup je n’ai pas eu mal et un mois après je suis resté bloqué. Je suis allé voir un ostéopathe qui m’a dit que c’était bien l’accident qui avait provoqué ça. J’ai été voir un kiné il y a pas longtemps et puis je suis allé voir un ostéo pour me remettre en place j’avais des points de blocages apparemment.

La première fois que je suis resté bloqué ça a duré 3 jours et puis depuis j’ai des maux de dos continuellement quand je me lève par exemple.

Et l’évolution depuis l’accident ?

J’avais de plus en plus mal mais la le kiné m’a fait du bien, j’y suis allé hier.

Il y a des choses qui déclenchent ?

Je ne sais pas, peut être le temps, après il y le sport forcément pour le dos c’est pas génial. On est sans cesse en train de sauter par tout donc je pense que ça ne doit pas être génial et puis souvent après les matchs j’ai mal au dos donc je pense que ça vient de ça.

Tu ne ressents pas le contraire ?

Non sur mon corps oui ça a un bon effet mais pas sur le dos. Après les matchs quand je prends le volant ça me tire mais ce n’est pas violent non plus.

Le dos t’empêche de pratiquer certaines activités ?

Non je ne pense pas, je le met de coté même quand j’ai mal au baskets je continue, je ne m’arrête pas à ça.

Et pendant les périodes douloureuses ?

Pendant les périodes douloureuses je ne fais pas grand chose, je reste dans le canapé ! repos ! canapé-lit ! je bouge un peu mais j'essaie de faire attention a mes mouvements.

Parce qu'on te l'a dit ?

Oui, il y a les parents derrière ! ils me disent de faire attention de ne pas trop tirer dessus quand je vais au baskets, ils me massent des fois quand j'ai vraiment mal.

Et le kiné ?

Il me fait des massages de dos et me met les électrodes des fois. Ca fait du bien.

Il me mettait dans des postures des fois en me faisant tourner la jambe . Je ne sais pas trop à quoi ça servait.

Et le moral ?

Ca va, je garde la pêché, il n'y a pas de retentissement.

Et le travail ?

La en temps qu'étudiant ça va, c'est vrai que les chaises ne sont pas très confortable mais bon (rire), ça se passe bien, c'est des cours magistraux dans la branche commerciale. Il y a mal de voiture par contre il faut une voiture confortable.

Et le sport ?

Toujours motivé, ça ne me dérange pas, sauf si j'ai vraiment mal et qua je ne peux pas continuer mais sinon non.

Ca t'angoisse des fois le mal de dos ?

Non pas vraiment, ça me trotte dans la tête quand je commence un match mais après je l'oubli assez vite, sauf quand c'est vraiment douloureux, je demande à sortir.

Tu prends des comprimés des fois ?

Doliprane de temps en temps.

Et l’évolution ?

Pour moi le mal de dos ça dure, même si on fait du kiné, je pense que ça va me rester, après je ne sais pas je peux me tromper. L’osteo d’hier m’a enlevé les points de blocage, après je verrais bien si ça continu. On m’a dit qu’il y avait des séquelles à vie dans les accidents, je pense que celui la je le garderais, avec des traces plus ou moins fortes.

Et c’est apparu un mois après…

Oui, le premier osteo, il m’a remis les cervicales en place.

Dans ta vie quotidienne

Ca ne me dérange pas, je ne m’empêche pas de faire des choses parce que j’ai mal au dos.

Et dans la famille

Les 2 parents ont mal aux dos, alors ont essayent de se partager un peu les taches.

Faut s’adapter on a pas le choix !

Entretien n°15

Parlez moi de votre mal de dos…

Le mal de dos ça fait des années que je traine ça, ça a commencer il y a 10 ou 15 ans.

Je suis quelqu'un qui travaille beaucoup, je suis un agent technique polyvalent, je suis un touche à tout, c’est pour ça que j’ai créé mon entreprise…J’ai fait beaucoup de métier et je pense que mon mal est dos est du à toute l’énergie que je met dans tout ça.

Quand j’étais petit je me suis cassé la clavicule, on m’a mis des anneaux claviculaire et peut être que ça ça a commencé à jouer sur ma colonne vertébrale ou sur l’ensemble osseux je sais pas. Si j’ai mal aux « reins » c’est par rapport à des efforts de manutention.

D’après ce que j’ai compris j’ai de l’arthrose qui fait que ça accentue les douleurs.

J’ai vraiment commencé à me poser des questions vis à vis de la douleur, je ne suis pas quelqu'un qui me plaint mais le dos tout mon entourage m’a toujours entendu dire que j’avais mal au dos, c’est récurrent, c’est pénible.

Il y a quelque année j’étais en conflit avec un de mes employeurs qui ne voulait pas reconnaître une de mes qualifications et en même temps il a été découvert par un médecin rhumatologue que je faisais de la fibromyalgie, les douleurs étaient toujours situées le long ou autour de la colonne vertébrale… Il avait lié mes douleurs avec le conflit et c’est vrai que j’ai découvert qu’une fois que j’ai traité ce conflit, un jour je me suis dit que je n’avais plus mal au dos, alors qu’avant ça me bouffait. Et puis je m’étais documenté à l’époque, et c’est vrai qu’au début qu’en on va voir un médecin et qu’on lui dit « j ‘ai mal la ; j’ai mal la » et pour se faire écouter, des fois on se dit qu’il doit nous prendre pour un fou ; quand on est en face d’un médecin rhumatologue qui sait de quoi on parle parce qu’il découvre que c’est la fibromyalgie, moi j’ai été effectivement soulagé de savoir que effectivement j’ai bien quelques choses, il y a bien un nom sur mon mal et ça c’est vrai que c’était hyper important à l’époque.

Et le retentissement dans votre vie quotidienne ?

Je le vivais mal parce que c’est vrai que dans mon entourage je me plaignais tout le temps de ça, moralement c’est usant. A force de dire à tout le monde j’ai mal au dos, parfois on ne le dit même plus, ça devient banal, on souffre intérieurement ; iL faut faire avec on a pas le choix de toute façon ça ne se voit pas quand on a mal, il n’y a pas de bleu, il n’y pas de sang, il n’y pas de bosse, il n’y rien c’est à l’intérieur. Ils se disent c’est un dépressif !

Ce qui m’a amené devant mon médecin traitant il y a 2 mois , c’est que je vais faire une cure. Par période dans l’année, et ça fait 2 ou 3 ans j’ai mal sur la colonne vertébrale, il y 2 points, on a découvert 2 hernies discales ; moi j’en fais pour le coup tout une montagne parce que ça me pince et que c’est très pointu très aigue comme mal mais c’est très périodique.

Et la depuis 3 mois c’est violent très aigu, et je me dis qu’il va falloir que je mette les pieds dans le plats que j’aille voir mon médecin traitant pour traiter et faire soit une cure soit opéré je n’en sais rien si c’est opérable.

C’est clair que je me pose beaucoup de questions sur ce mal la qui pourri mon moral c’est évident ! Mais je ne veux pas trop me plaindre parce que après quand on me dit…

Je suis aller faire une IRM il n’y a pas très longtemps, parce que j’ai demandé à mon médecin de faire la totale vu comme la douleur me pourrissait la vie je voulais voir ce qui se passe vraiment.

Et pendant les périodes aigues vous faites quoi ?

Rien, je ne prend pas de médicament, je ne prend rien parce que moi je ne suis pas médicaments, alors c'est peut être un tort je ne sais pas.

Et vos activités dans la vie quotidienne ?

En terme d'activité, en y réfléchissant, ce mal de dos qui est la de plus en plus fort, ça survient au moment ou j'ai arreté le sport et au moment ou j'ai créé mon activités c'est à dire juin 2009. Je consacre mon temps a mes 2 activités et je ne fais plus de sport, alors qu'avant je faisait du footing régulièrement, j'allais à la piscine sur mes jours de repos, je faisais du sport et j'étais mieux dans ma peau. Et c'est vrai que quand on fait du sport au début c'est difficile mais après on habitue son corps à l'endurance et on va bien.

Depuis 2009, il y a le stress de trouver des clients, je suis investi à 300% et je ne fais plus de sport.

Parfois j'essai de me relancer mais ça marche pas, parce que le quotidien, l'administratif de mon entreprise prennent le dessus et comme je suis obligé de le faire, je le fais au détriment du sport. Ce qui prend le dessus c'est l'activité d'entreprenariat, c'est presque du 24/24H. J'ai un stress permanent de par mon travail, je vis au jour le jour financièrement parlant. Donc les priorités changent.

C 'est pas un manque de motivation parce que j'ai envie mais c'est un manque de temps, mais le quotidien de l'entreprise me prend la tête.

Mais la j'ai décidé de prendre ma santé en main donc cure, IRM, consultation rhumatologue et on va jusqu'au bout, au moins pour que déjà je me sente mieux dans ma tête et dans mon corps.

A moyen terme je veux changer d'emploi pour essayer de retrouver une sérénité, et reprendre le sport.

Le radiologue qui m'a fait l'IRM m'a dit il n'y rien de grave, donc j'étais rassuré.

Mais quand on a mal et que le matin faut essayer de trouver la position qui va bien, parfois c'est compliqué.

En plus si la veille j’ai fait une journée difficile, ça se ressent sur mon état de santé.

Mais maintenant j’ai envie de me soigner.

Et puis je voudrais savoir quel comportement adopté pour la suite, parce que j’imagine bien que l’arthrose ca va se dégrader.

J’ai pas repris le livre que j’avais acheté mais je pense que ca va se dégrader, mais je voudrais que ca se fasse le plus lentement possible. Ca m’interesse de savoir quel comportement je dois avoir pour que ca se dégrade lentement. C’est quoi les geste au quotidien que je dois avoir.

Même si si j’ai bien compris mon état n’est pas alarmant pour les médecins, alors peut être que je me fais des films.

Pendant les vacances…

Je n’en prend pas beaucoup, et en temps qu’entrepreneur j’en prend jamais parce que le chiffre d’affaire il est pas fait. Il n’y a que le dimanche que je bosse pas.

Je n’ai jamais eu d’arrêt de travail lié à mon dos ! le trou de la sécu c’est pas moi !

Je voudrais refaire du footing, j’avais racheté des baskets, ma vie privée est compliquée aussi… je pense que depuis 2009 ça s’est aggravé parce que je ne fais plus de sport.

Je fais toutes les activités de la vies quotidiennes, le seul moment de repos c’est quand je regarde un film le soir devant la télé, c’est mon moment de détente.

Ma vie d’entrepreneur m’a mis des bâtons dans les roues au niveau de mon dos.

Je voudrais retrouvé un équilibre de vie, être mieux dans ma peau mais il va falloir que je fasse des chois de vie différent

Il y a que le sport qui pourra m’aider a passer cette étape la, mon médicament c’est le sport !

Entretien n°16

Parlez moi de votre lombalgie…

C’est gênant… enfin au niveau de la vie quoi. Parce que en voiture déjà il faut qu’on s’arrête souvent. Il faut que je fasse attention à tout ce que je fais.

Quand je veux faire du jardin par exemple, j’adore le jardinage mais je ne peux en faire que 10 minutes ¼ d’heure sinon j’ai mal. Et encore je le fais souvent avec une ceinture.

Pour les courses je ne peux pas porter de choses lourdes, donc par exemple les packs de lait, je dois les prendre un par un. Je ne peux pas parce que j’ai mal.

Maintenant c’est sur que j’appréhende plus souvent la douleur qu’avant. Et les crises sont plus rapprochées et plus longues qu’il y a dix ans ou cinq an, peut être que je les appréhende mais bon je ne me pose pas trop la question.

J’essaie quand même pour pas me coincer carrément et pas rester alité de faire le moins de chose que je sais qui ne me vont pas.

Ca m’est arrivée plusieurs fois de rester alitée, il y a des jours ou le matin je ne peux pas me lever même pour aller au WC, je suis tellement crispée, et coincé que je suis obligé d’appeler et d’avoir un injectable pour arriver à me lever. C’est cette crise là que j’essai de ne pas re-avoir.

J’en avais assez souvent quand je travaillais, ça m’arrivait surtout quand j’étais au travail, surtout les derniers temps dans mon travail je faisais deux jours et après j’avais mal au dos. C’est dès que je recommençais le travail, dès que je me penchais, il y avait plein de chose qui me faisait mal. Ca a commencé au travail, quand j’ai commencé à avoir mal au dos on a pas su tout de suite ce que c’était quoi, après j’avais une protubérence donc. Ca c’est accentué au fil des années mais je ne me rappelle plus bien quand. Au bout de 2 jours j’avais du mal à travailler. Et donc on m’a passé un autre scanner et on a vu qu’il y avait une autre hernie. Et la de toute façon c’est l’année ou je me suis arrêtée de travailler, ça correspondait à une pré-retraite.

L’activité physique en période de douleur c’était dure c’est la que j’ai pris pas mal d’anti inflammatoire. J’arrivais quand même à continuer j’étais à temps partiel à 80%, des fois j’avais 3 jours et donc s’était là pendant ces périodes de congés que je me coinçais en faisant presque rien à la maison, des fois juste en ouvrant un baril de lessive.

Le sport je faisais un peu de piscine à l’époque et un peu de marche mais moins que maintenant. La piscine quand j’y suis je ne sens plus mon corps dans l’eau, j’ai l’impression quand j’ai mal, je suis bien, je me décontracte et même dans l’heure qui suit je suis plus décontracté. Je vais à Royat tonique aussi c’est chaud ça fait du bien.

La marche aussi, bon j’en ai moins fait au mois de juin parce que j’avais mal et je ne voulais pas embêter. Parce que quand on commence quelque chose on est en groupe, on part pour trois ou quatre heures et quand je ne me sens pas bien j’y vais pas parce que j’avais trop mal et puis j’avais peur de faire arrêter la marche. On fait des WE mais par exemple je ne vais pas aux semaines plus loin parce que je sais que ça ne me convient pas après le trajet en voiture.

Le moral, je crois que c’est quand même le dos qui me gène le plus. J’ai eu donc ça ce mal de dos qui était quand même assez récurent plus l’hémochromatose, je me demandais lequel des 2 jouaient le plus. L’Hémochromatose pour le moment j’ai une rémission par contre le dos je ne vois pas ce que je peux faire de plus. Je ne me pose pas trop la question de savoir comment ça va évoluer parce que je vois pas bien.

Mon entourage sais que j’ai mal au dos mais souvent il l’oubli sauf quand je suis bloquée, ou la il faut qu’il m’aide. Et les intervenants médicaux…(hésitations), j’avais été voir un chirurgien il y a pas mal d’année quand j’étais très gênée, justement pour voir, et il m’a posé la question « vous vous arrêtez combien de fois dans l’année ? », je lui dis « je me suis arrêtez une fois » il m’a dit « c’est pas grave », donc les autres fois je me suis ça ne compte pas tout ce que j’ai mal. C’est l’arrêt de travail qui compte, j’ai trouvé ça un petit peu…

Maintenant je sais que même si j’ai très mal il faut que je me relève et que j’essai de marcher parce que sinon ça dure encore plus. Je m’en suis rendu compte en le faisant, qu’il faut que je dépasse cette douleur qui me vrille les lombaires et après si je marche je suis mieux, j’ai dépassé un stade.

Et après le reste c’est des petites douleurs à droite à gauche, ou si j’ai mal marché aussi.

Si on va quelque part il faut que je fasse attention a ne pas me coincer. Je ne vois pas partir marcher si je gène tout le monde.

Ici je marche une fois avec le club 4H, une fois avec mon mari mais on ne fais qu’une heure parce qu’il ne peux pas plus lui. Quand je suis seule je marche moins longtemps je ne vais pas dans les bois toute seule

FOCUS GROUPES

Focus groupe n°1

Durée : 30minutes

4 participants + 1 animateur + 1 observateur

Depuis quand et comment est apparue votre lombalgie ?

- 2010, accident du travail, et là-dessus il y a un peu de vieillerie certainement. La douleur est devenue constante. Avant c'était par épisodes et maintenant c'est constant.
- Moi ça fait un an et demi à peu près, j'ai fais une mauvaise chute qui a altéré ma scoliose j'dirais et les douleurs sont apparues à partir de là. Et pas du tout avant. Et maintenant j'ai mal tout le temps, surtout debout.
- Premier épisode en 2003, à cause d'une mauvaise manip' au travail. Au bout de 4 mois j'ai repris le travail et j'ai re-eu un épisode en 2008. Et là depuis octobre 2012, surmenage. J'ai pas pu me lever un matin. C'était dur au début, les premiers mois, maintenant je suis moins fatigué mais j'ai mal.
- Moi c'est un accident du travail. J'ai eu plusieurs arrêts de 10 jours, jusqu'au dernier accident du travail qui remonte au 15 janvier 2013. ça passe toujours pas, c'est de pire en pire, et j'peux pas être assis trop longtemps. La douleur, avant, elle passait au bout de 10 jours on va dire.

Et dans votre vie quotidienne comment cela se ressent-il ? Quels en sont les impacts ?

- Des bons p'tits plats.
- Des bons p'tits plats voilà.
- Moi c'est surtout que je suis très énervée, tout le monde en prend pour son grade. Je tourne en rond, j'arrive pas à canaliser la douleur. De pas travailler, moi ça me rend malade, de pas avoir d'avenir. Qu'est-ce qu'on va faire de moi ? La monotonie, le quotidien de tous les jours, la douleur tous les jours, les « cachetons » tous les jours … au niveau du sport, moi qui suis sportive, alors là alala…, au bout d'un moment le moral… on prend tout en négatif en fait.
- Y'a de gros problème aussi au niveau du temps.
- Oui au quotidien.
- Moi je vois par exemple un meuble en bas, je suis obligé de me mettre sur les genoux, j'préfère faire gaffe.
- Les autres, ils veulent, mon mari qui veut m'aider et qui dit « attend attend attend » et je lui dis « mais c'est bon je suis pas handicapé non plus, je vais le faire, je vais le faire, je vais le faire », mais les autres veulent le faire pour moi.
- Oui avec la douleur permanente, ça empêche pas. Moi je vois chez moi je bricole tout le temps.
- Oui les choses banales du quotidien, faire la vaisselle ou faire du shopping en général c'est une torture quoi. On peut pas faire les choses du quotidien comme les autres quoi.
- La moindre activité, rester sur place, piétiner, c'est une torture.

Cela a-t-il changé beaucoup de choses dans votre vie quotidienne ? Avez-vous adapté votre comportement ?

- Les mouvements surtout à mon boulot.
- Ca nous rappelle vite à l’ordre. On peut pas le faire ou si on le fait et qu’on a mal, on doit reprend à zéro pour éviter de nous bloquer.
- On s’y reprend à deux fois.

Et sur votre travail, est ce que cela a eu un impact ?

- Pour moi la position assise est confortable donc y a pas de soucis à ce niveau là mais dès que j’ai un cours où je dois rester debout … mais bon j’essaye de subir et d’écouter donc je me force. Mais des fois je peux pas y aller tellement j’ai mal.

Et au niveau de l’AP est ce que vous pratiquiez avant ? Et qu’est ce que c’est pour vous ?

- A mon niveau, au niveau de mon boulot, c’est de l’activité physique, donc le soir en rentrant j’avais pas spécialement envie d’aller me remettre au sport. J’avais plutôt envie de voilà, de me poser. Le dimanche aller faire du vélo avec les gamins ça va bien mais bon j’avais pas spécialement envie d’en faire.
- Moi je faisais du sport avant, avant l’armée, après j’ai plus le temps.
- Ha oui si.
- Moi j’étais une accro, je faisais des compèt’, sport collectif, aérobie, stepp. J’en faisais 10h par semaine. Enfin avant les enfants, après c’est à nous d’organiser et c’est autre chose et on pense à d’autres choses, à l’activité des autres. Et pareil moi je suis aide-soignante, je fais les 3/8 quand on a 8h debout dans les services on a plus envie de sortir et faire du sport. Oui effectivement, c’est de l’AP, tourner les malades, soulever les malades, faut dire que quand même, on n’est pas en dilettante.

Depuis le début de vos lombalgies, cela a-t-il eu un impact ?

- Bien évidemment, j’ai même plus envie de me bouger parce que je sais qu’une demi-heure de marche je vais le payer après. Je vais avoir mal, avoir du mal à m’endormir.
- Les médecins qui prescrivent de la marche pour le mal de dos, c’est zéro.
- Moi j’essaye parce que j’ai envi des fois d’aller avec la famille. De me sortir de cette maison mais je sais que je vais le payer donc des fois je me dis non.

Du coup vous avez complètement arrêté ?

- Moi à la base je suis pas du tout sportive, donc non.
- Moi je fais des travaux chez moi.
- Même passer la tondeuse c’est de l’activité physique.
- A la maison c’est moi qui passe l’aspirateur, je sais que ça me fais mal mais je me force, je le fais en plusieurs fois. Je préfère à la limite aller faire autre chose, même aller marcher même pendant une demi-heure même si je vais avoir mal, que de rester à rien faire.

Qu’est-ce qui vous freine à faire de l’AP ?

- La douleur.

- L’envie.
- La motivation.
- Et aussi la motivation.
- à des moments on la perd. Le mal pour le bien des fois, on sait pas bien, on comprend pas bien. On comprend qu’il faut marcher, comme n’importe qui, comme les personnes âgées, tout le monde doit marcher dans la journée. Mais on a beau essayer de faire, où est le bien ?
- Faut avoir la wii.
- Je l’ai moi, j’ai essayé ça. C’est pas haut et bah j’ai perdu l’équilibre, y a tout qui est perdu.

Et qu’est ce qui pourrait vous pousser à faire de l’AP ?

- Etre cadré.
- Plus avoir un coach, qui nous pousse forcément, qui nous aide à faire l’exercice, à nous corriger. Quand on est tout seul, voilà… on a plus tendance à se laisser aller, on fait pas l’exercice à fond.
- Ne pas avoir mal.
- Etre encadré dans le mouvement car moi j’ai peur de me faire mal, d’empirer ce que j’ai, d’aggraver.
- Pas trop réfléchi… oui ptre être encadré dans les mouvements.

Vous sentiriez vous capable de pratiquer de l’AP chez vous si au préalable vous aviez eu des séances pour apprendre les exercices à faire ?

- C’est la motivation..
- Après c’est la motivation, enfin j’pense.
- C’est la motivation ça c’est sûre.
- C’est l’envie de faire.
- Après je m’occupe, enfin je bricole.

Focus groupe n°2

Durée : 22 min

3 participants + 1 animateur + 1 observateur

Quel est le retentissement de vos lombalgies dans la vie quotidienne ?

- Le ménage j’suis allergique (rire). Quand j’étais petit et que je prenais le balai, ma mère me tapait sur la tête et me disait « lâche-ça ». Depuis je suis allergique au balai (rire).
- -(rires général)
- N’empêche, passer l’aspirateur ça va, passer la serpillière ça pique.
- Prends un manche plus long (rire).

Et tout ce qui est cuisine, bricolage, repassage ?

- La cuisine du moment qu’elle est adaptée ça va. Si vous avez un plan de table standard à 80, moi je peux pas. Moi j’ai fait ma maison au détriment de ma femme, tous mes plans de travail sont à 1m.
- - Mais ta femme elle fait 2m ?
- - Non, elle fait 1m50. Pour attraper les assiettes elle en chie (rires).
-

Et le reste des activités du quotidien ?

- Le bois ça coince.
- Quand je m’appuie sur le chariot ça va (rires).
- Certains jours ça va aller, d’autres non.

Qu’est-ce que l’activité physique pour vous ?

- Ben le sport, en règle générale.
- La marche, la vaisselle, le ménage, le câlin avec Madame.
- Non ça c’est du travail (rire) !!!
- Plaisanteries mises à part, faut en parler aussi. Ça en fait partie.
- Et j’aimais plutôt cette activité moi ! Quoique le dos des fois ne le permet pas.
- Voilà c’est ça !

Vous êtes gêné par vos lombalgies pour cette activité-là ?

- Non (rires).
- Ça peut arriver.

Qu’est-ce qui vous empêche de faire plus d’activité physique ?

- Un peu l’état feignant aussi.
- La motivation des fois c’est…Quand on a mal on est moins motivé.
- Le temps.
- Parce que des fois les journées elles sont un peu courtes. Quand on commence à 6h et qu’on finit à 20h le soir c’est…
- Moi jusqu’à présent c’est les prescriptions médicales.

Les médecins vous avaient recommandés de ne pas faire d’activité physique ?

- Mon médecin traitant, mon neurochirurgien, mon kiné, et ma femme, surtout Madame.

Pratiquiez-vous du sport auparavant ?

- Un peu oui.

Avez-vous continué d’en faire ?

- Ouai.

Comment avez-vous adapté le sport à vos douleurs ?

- Moi je suis plus allé à la salle, je me suis fabriqué ma salle dans mon garage. Du coup je me suis bien ruiné, avec du poids et de la fonte.
- Pourquoi t’as été acheter du matériel ? Tu prends un bout de bois avec deux troncs d’arbre et puis voilà ! Tu changes les roues du tracteur à la main et puis voilà !
- (rires)
- Et la course à pied j’ai arrêté parce que bon, voilà, trop douloureux.

Est-ce que le fait d’avoir un travail physique peut entrainer une baisse de motivation à faire de l’AP en dehors ?

- Non moi question physique au boulot…. Je suis assis pratiquement toute la journée. A part prendre le balai de temps en temps… parce que sinon rester 8h à attendre ça m’emmerde quoi, j’aime pas rester….

Aimeriez-vous faire plus d’activité physique ?

- Si je pouvais oui. Si le dos pouvait suivre oui.
- Ah ben reprendre, reprendre oui.
- C’est vrai il faut avoir un peu le temps après.

Qu’est-ce qui vous aiderait à reprendre ?

- Bah déjà quand on a moins mal on a plus le moral. Quand on a mal c’est vrai que…
- Avec les beaux jours c’est plus facile.
- L’hiver c’est vrai que... Quand on sort du boulot on a pas envie de se promener tandis que maintenant avec les jours qui se rallongent…

Est-ce que le fait de faire du sport en groupe vous motiverait d’avantage ?

- Peut-être oui.
- Je pense aussi.

- C’est toujours plus motivant d’être avec quelqu’un, sans parler d’un groupe, juste avec un collègue, on se motive. Parce que si tu veux y aller tout seul t’es là « ohh pas ce soir, demain ».
- On repousse toujours l’échéance quand on est tout seul.
- Tandis que là avec Madame maintenant qu’il fait beau c’est « allez on va se promener, bon allez on y va ».

Seriez-vous plus enclin à pratiquer de l’activité physique au sein d’une infrastructure ?

- Chez nous on a peut-être la mauvaise habitude de faire des mauvais mouvements, les gestes qu’il faudrait pas faire, alors que encadré c’est mieux, je pense.
- On va pas aussi loin dans l’effort aussi, quand t’en a marre t’arrêtes. Quand t’es à la maison que t’a fait tes 20 min de vélo tu dis allez c‘est bon. En salle on travaille le cardio c‘est 45 voire 1h30 min , t’y va c’est tout, tu pédales. T’en a marre tu continues.

La présence d’un coach vous aiderait ?

- Bah déjà la mauvaise position il va nous la dire.

Si on vous apprend des exercices, allez-vous les reproduire chez vous ?

- Oui, bien sûr.
- Je sais pas si ça va marcher. Ça va marcher un temps puis après…
- On en fera dans un temps puis après pfff…

Qu’est-ce qui vous aiderait à poursuivre ces exercices plus longtemps ?

- Un suivi. Ne serait-ce qu’un suivi par courrier ou par un coup de téléphone. Même un suivi par mail.
- Ça j’avais dit à Vichy ça. Parce que j’en ai déjà fait de ça là-bas.
- Comme ça on se fixe un impératif et on essaye de s’y tenir. Ça dépend de la motivation de chacun après.
- C’est pareil quand t’a fait ta journée de boulot…Des fois je prends à 5h du matin et je sors à 20h-21h. T’arrive t’a pas envie de faire des exercices. Tu t’occupes un peu de la cht’it et après c’est dodo.
-

Quelle est l’attitude de vos familles par rapport à l’activité physique ?

- C’est plus le fait de me mettre dans un cocon.
- Ouai, « fais pas ci », « Va pas faire du bois tu vas avoir mal au dos ».
- J’ai recommencé à faire mon petit vélo, elle, elle allait faire ses cours en salle et moi je suis resté sur le côté vélo. C’est sympa.
- Moi j’ai revendu le vélo la semaine dernière ! (rires)

- Moi c’est le terrain à côté de chez moi c’est pas trop…. Ça descend mais quand tu reviens faut tout remonter…
- Marche à pied ça me va très bien, 2 km.

Et vous arrivez à vous motiver pour la marche à pied ?

- Oui. Avec les beaux jours oui. C’est surtout ça maintenant. Les beaux jours… Ce WE ont a fait que ça, samedi dimanche. Le soir quand elle rentre quand elle finit pas trop tard on va faire un tour. Ça fait que 2 km mais bon tous les soirs un petit peu ça fait pas de mal.

A quelle fréquence aimeriez-vous faire de l’activité physique ?

- Dans un contexte idéal, tous les jours.
- T’es un grand sportif toi ! (rires)
- Moi je veux juste retrouver ce que j’avais avant en fait. Pas plus.
- Bah oui normal.
- Mais surtout on vieillit, ça va baisser.
- On vieillit, on vieillit, on n’est pas mort on n’est pas grabataire ! On n’a pas de déambulateur, faut y aller !
- Oui dans l’idéal en refaire le plus souvent possible.
- Pas tous les jours.
- Y’a des jours c’est vrai qu’on va être motivé, puis d’autres jours y’aura la fatigue…
- Y’aura des douleurs…
- Voilà c’est..

Avec un suivi arriveriez-vous à vous motiver pour les exercices à domicile ?

- Il y a une motivation du coup derrière. De se dire bah au bout de 2 mois on va voir nos progressions…C’est une forme de motivation.

Quel serait la fréquence de suivi idéal selon vous ?

- Moi j’aurais dit au départ tous les mois. Et après…

Et vous ?

- Ça va être dur quoi. Bah si vous voulez j’ai jamais fait de sport. C’est dur de s’y tenir. Je vois bien chaque année j’essaye et puis j’abandonne parce que…
- Tu sais tu viendras avec moi, t’habites pas loin en plus de chez moi. Tu viendras avec moi faire du bois. Rien que de te baisser et te relever tu verras !
- Non ça ira !
- C’est le plus dur, couper ça va mais après, tout le ramassage qui est le plus chiant.
- Je conduirais le tracteur.

La motivation pêcherait malgré le suivi ?

- Oui je pense. Après c’est une histoire de temps aussi. Y’a des semaines je les fais du lundi au dimanche des fois. Ca dépend…Après, j’ai pas envi... A part l’été, de barboter un peu, mais sinon…

Quelles *seraient les autres sources de motivation pour pratiquer l’AP ? La proximité des infrastructures ? En avez-vous besoin de ses infrastructures ?*

- Les structures sont un peu loin de chez moi ouai.
- Ah bah pour être bien encadrés au début ouai c’est sûr.
- C’est plus facile d’avoir un encadrement.
- Toi tu vas faire de la muscu chez toi tu vas faire un mauvais mouvement tu vas être coincé. Tandis que en structures, t’es suivi quoi. On va te dire « tiens lève les genoux, rapproche les jambes, écarte les jambes ».

Pensez-vous que les supports numériques pourraient vous aider ?

- Moins, en tout cas pour moi perso moins. Il me faut quelqu’un qui me gueule dessus .
- La Wii c’est complètement débile ce truc, c’est là où on se fait plus mal que faire des exercices en réel. Je vous dis ça franchement, je trouve que les Wii c‘est…
- En guise de complément.
- Mais alors des bons exercices. Parce que tous les jeux qu’il y a en ce moment à la Wii...
- Moi je dirais en complément, par exemple par rapport à des semaines trop chargées, se dire allez je mets une petite demie heure de machin je vais essayer de la faire.

La ceinture lombaire vous permet-elle de faire plus d’activité physique ?

- Non. Ça soulage, surtout si on porte des charges lourdes.
- Ca maintien un peu plus quoi.
- Oui ça maintien, ça soulage.

Et vous la portez souvent ?

- Le dimanche (rires). Nan , quand…
- Quand j’ai des charges lourdes à transporter, parce que sinon c’est.. c’est comme tout faut pas s’habituer à la porter tout le temps sinon ça sert à rien.
- L’été ça tient chaud.
- Je la porte essentiellement en voiture. Même pour conduire des fois je la mets, malgré qu’il y ai des sièges adéquats, je la mets de temps en temps.

Focus groupe n°3

Durée : 39 min

3 participants + 1 animateur + 1 observateur

Qu’est-ce que l’activité physique pour vous ?

- Ben l’activité physique ça peut être le sport, ou le travail si on a un travail physique, ou le jardinage, le bricolage.
- Les loisirs ça peut être physique aussi.
- Moi c’est mon but ça ouais c’est de faire un exercice après quoi, enfin je veux dire admettons que je sors d’ici, on me dit voilà faudra faire toujours le même geste pour toi, par exemple tous les matins pendant 1h faire ça, pour ce que j’ai, et ben je le ferais, je m’adapterais, parce que on me dit toujours bon faut aller à la piscine, mais si tu nages mal, tu te fais mal plus qu’autre chose, puis l’eau est froide alors… Si vous voulez, c’est bien beau les conseils mais voilà, voir par rapport à ce qu’on a. Le but c’est qu’après on continue une activité, par exemple le sport que je rêvais faire, moi c’était le tennis mais… on oublie. Mais voilà on me dit faut que tu fasses ça tous les jours, par exemple une demie heure de vélo, une demie heure de vélo d’appartement, j’sais pas les trucs dont m’avait parlé le cardio. Si faut acheter tout ça et le faire, c’est ça que j’attends…mais les bons gestes quoi. Aussi répétitif tous les jours, parce que pour moi pour mes rhumatismes c’est ça, c’est décoincer tout ça quoi, mais les bons gestes, j’attends ça quoi.
- Ben c’est tout ce qu’on voudrait faire et qu’on ne peut pas faire à cause du mal de dos, le sport, le…Le quotidien quoi.
- Le quotidien ouais.
- Sortir d’un canapé c’est un exploit déjà. c’était déjà un exploit à une période donc euh…
- Pouvoir refaire des loisirs qu’on peut plus faire, pouvoir les refaire si on peut si c’est possible. Moi c’est la moto, j’ai été obligé d’arrêter. Parce que ça tirait trop sur le dos. J’ai été obligé de la vendre et d’arrêter. J’aimerais pouvoir faire des ballades et tout ça, mais c’est pas très conseillé pour le dos.
- Bah non non puis quand t’a mal c’est pas agréable ; c’est ça le problème, quand tu fais un truc et que tu souffres.
- Même en voiture. Suffit qu’on aille en vacances qu’on a 4h de route c’est horrible. Obligé de s’arrêter, de pouvoir se mettre comme ça pour s’étirer, c’est vraiment horrible.

Votre mal de dos a-t-il eu des conséquences sur votre quotidien ?

- Sur tout, ça change tout.
- Ça me gâche la vie.
- Chaque chose que je fais dans la journée, bah si j'avais pas eu mal au dos je l'aurais pas fait pareil. C'est différent. Je fais plus attention, mais c'est la douleur tout le temps, en permanence.
- Ben oui parce qu'en fait on fait des trucs qu'on est obligé de faire comme travailler parce qu'il faut bien vivre, et puis le reste le jardinage parce que personne va le faire à ma place, alors que je sais très bien que même en mettant une ceinture le soir je vais avoir mal quoi. Y'a des choses qu'on est obligé de faire, mais justement qu'on voudrait faire sans avoir mal. Pour ma part au quotidien c'est bien pénible. A la longue…
- C'est plus un plaisir quoi…
- On est obligé de le faire en ayant mal en fait c'est ça.

Vous essayez tout de même de faire les activités du quotidien ?

- Ah oui moi je me dis quitte à avoir mal.
- Y'a des obligations aussi.
- Oui mais y'a des choses qui vous rappellent à l'ordre. Y'a des choses…

Est-ce que avant l'apparition de votre mal de dos vous pratiquiez un sport ?

- Moi je faisais du tennis. Et j'avais acheté un Buggy pour en faire avec ma fille. Je l'ai gardé un an, c'était excellent, j'ai toujours la vidéo et tout avec ma fille à côté, sans faire des sauts hein. Je l'ai vendu, je pouvais plus tenir dedans, donc euh… Si je pouvais remonter juste sur un quad, juste pour aller me promener, là c'est le… Sur la route hein, juste sur la route. Des trucs comme ça quoi, au quotidien quoi, ça me… voilà. J'ai des beaux-frères qui me disent ouais viens on va voir la porte-ouverte de ça. Bah ça me… Voilà je les regarde les trucs mais...je peux pas…voilà au quotidien les loisirs c'est finis quoi. Juste ça quoi.

Vous avez l'impression que votre vie tourne seulement autour du travail ?

- Voilà, travail, maison et tout. Argent c'est bien beau d'en gagner mais si on peut même pas le dépenser c'est même pas la peine. Voilà j'en suis arrivé là quoi… J'avais même acheté un VTT avec suspension et tout. J'ai commencé par le vélo d'appartement tout doucement. Puis quand je suis remonté dessus j'en ai pleuré. Je pouvais pas rester sur la selle. Ça me tannait quoi. Le dos j'avais tellement mal, j'en ai pleuré j'ai arrêté quoi. Mes copains y m'ont dit qu'est-ce qui t'arrive et tout. J'ai craqué quoi, j'ai craqué, j'en pouvais plus. Et là ça fout un coup au moral. 38 ans vous vous dites… Le vélo c'est…j'le prends, j'l'ai jeté, j'ai rangé. C'est ça c'est le quotidien quoi, c'est le quotidien, c'était une ballade quoi , on allait faire une ballade

quoi…Avant j’ai fait du vélo d’appartement comme ça j’aurais l’air moins bête avec les copains…Voilà je l’ai rangé. Alors la ça vous fout le moral à zéro quoi.

- Ben moi j’ai toujours fait du sport, j’ai fait 18 ans d’armée donc j’étais assez sportif. Et là bon par rapport à lui c’est que j’arrive à faire une sortie en vélo mais c’est après que j’ai mal quoi, pareil avec la course. Voilà c’est comme le travail quoi, c’est chaud donc euh…Mais le soir après la douche ou en regardant la télé ou dans le lit on le paye quoi.
- Moi je fais plus de sport. Je faisais de la musculation, y’a pas longtemps, j’en ai fait pendant un an. Ça allait mieux au niveau de mon dos. Mais après en fait quand j’ai commencé à porter des charges plus lourdes, parce que…pour me muscler plus. Après ça me faisait des grosses douleurs. Donc du coup bah j’ai arrêté, pour l’instant, tant que ça va pas mieux. Après voilà bah c’était de la moto que je faisais tout le temps, tout le temps tout le temps. Je faisais des acrobaties puis j’ai été obligé d’arrêter parce que c’était des acrobaties que je peux plus faire maintenant. Je suis plus aussi souple, parce que mon dos maintenant m’en empêche, c’est… Après si c’est pour tomber et se faire encore plus mal c’est pas la peine quoi.

Donc c’est au quotidien ?

- Ah oui c’est au quotidien.

Qu’est-ce qui vous empêche le plus de pratiquer une activité physique ?

- La douleur.
- La douleur et puis des fois même si on a pas mal tout de suite, de savoir qu’on va avoir mal plus tard, dans 2h, ou juste après, 3h après, ça va être une douleur énorme. Des fois on tente quand même, et après on a une douleur et on dit si on avait su j’aurais peut-être pas fait. Mais des fois on est obligé de le faire.
- Bah oui voilà t’es obligé des fois.
- Bah oui c’est la douleur. J’ai même fait du vélo et de la course, contre-indiqué parce que le kiné ne voulait pas trop que j’en fasse. Je me suis dit je vais essayer quand même voir où j’en suis. Et puis tout de suite bon j’ai senti que j’avais mal quoi, avec les chocs…

Y a-t-il d’autres freins que la douleur à la pratique d’activité physique ?

- Si on enlève la douleur déjà on n’est pas là. Et puis on vit, on est heureux quoi. Le beau temps arrive, le moral est retrouvé, on peut faire quasiment tout ce qu’on veut sans la douleur.

Il y a également un retentissement sur le moral ?

- Ah bah oui, quand on voit pas le bout, qu’on a mal malgré les antidouleurs, les anti-inflammatoires, malgré la kiné. Le résultat est le même, ça devient long et ça agit sur tout quoi.
- Plus ça passe et plus on prend des cachets ; et après du coup les doses doivent être augmentées, augmentées, et après du coup y’a plus de doses… on est arrivé à un stade après ou y’a plus rien d’autre… Les cachets ça fait un mal pour un bien quoi, un bien pour un mal. ça soigne d’un côté, et ça fait du mal de l’autre côté.
- Après on relativise aussi, on apprend à vivre avec la douleur. Et on se dit que y’a plus malheureux que nous, y’a... quand on voit des gens qui sont handicapés pires que nous bah…mais quand même ça agit sur la tête.
- Quand on en voit qui ont rien et puis qui font rien, qui font pas de sport moi ça me……. Ça me rend malade quoi. Ah ça me rend malade. Ah j’ai des cousins, pfff…. Si j’avais la santé comme eux…Voilà c’est au quotidien quoi c’est…
- Rien que quand je vois les gamins qui peuvent pas faire 3 km à pied qu’on est obligé de les emmener en voiture et tout, pfff….

Vous avez envie de bouger ?

- Ah oui. On bouge quand même, enfin pour ma part, des choses que je fais que je devrais pas faire mais bon si je le fais pas personne va le faire donc heu…
- Voila

La motivation pour pratiquer de l’activité physique est toujours intacte ?

- Ah oui oui oui.
- Oui.
- Elle est toujours là oui. C’est physiquement que ça suit pas.

Y a-t-il eu un impact au niveau social et familial ?

- On est bien entouré quoi.
- Bah moi y’a eu un petit impact c’est que ma copine des fois elle me dit j’aimerais qu’on fasse ça ça ça et des fois on peut pas. Se balader, faire des choses…Je sais pas, des petites ballades ou quoique ce soit, on peut pas. Parce que je sais que j’ai mal et voilà. Pour partir en vacances, j’suis parti en vacances, et je suis le seul à conduire. J’ai fait 13h de route, et j’suis arrivé là-bas bah j’étais plus malade que.. Je suis arrivé là-bas je suis resté couché tout le temps parce que en fait j’étais là-bas pour rien… Je suis allé là-bas pour la première fois je me suis dit je vais passer un bon moment, mais en fait non, c’était le contraire, vraiment le contraire.
- Moi c’est pareil. J’étais tellement en crise au mois de décembre. On avait loué un chalet avec des amis dans les Vosges. Ben je dis j’abandonne et tout ; « Ben non vas-y viens avec nous ». Alors le père d’un beau-frère nous a prêté sa voiture qui est

vachement confortable et tout pour que j’y aille et tout. J’ai fait le tour du lac, j’me suis entravé dans une racine, ça m’a foutu…je pouvais plus bouger. Si j’avais pas le beau-frère qui est costaud pour me ramener à la voiture, alors là…J’passais mon temps aux cachets et sur le lit. Alors là vous vous dites les vacances, super ; et je prenais des cachets pour justement retourner avec eux pour me promener tout ça. J’ai fait que ça. Alors là, le réveillon… Le moral c’est vrai que…Là la route, pfff, c’était horrible ; Pas en voiture, c’était…fatigué quoi...et j’étais bien que couché. Donc là vous faites pas grand-chose.

- Moi j’voulais essayer de faire, j’ai jamais fait de ski donc j’ai voulu essayer d’en faire. Et le jour où je devais y aller, j’avais énormément mal au dos. Et du coup j’ai dit c’est pas la peine d’y aller. J’voulais en faire du snowboard des choses comme ça, mais j’ai pas pu en faire ; J’ai mal et j’me suis dit si j’tombe ça va être encore pire… en fait ça fait vachement réfléchir. Parce que y’a des choses qu’on a envie de faire, et avant de le faire on va réfléchir et se dire : est-ce que ça va me faire plus mal ou pas?
- Ouai un truc tout bête on a été au Portuquad pour les quads avec les beaux-frères pas plus tard que ce week-end. J’avais pris les cachets et tout, la ceinture ; J’ai dit on y va. Si jamais on en essaye un, je vais essayer de monter dessus quand même, même sur le goudron, juste pour…ben voilà quoi . C’était… J’avais prévu quoi, la ceinture et tout quoi. Je l’avais pas dit aux beaux-frères, la ceinture sur moi et tout. Et voilà c’est des trucs… j’me dis si on va là-bas, s’ils nous font essayer, ça serait bien de monter dessus quand même ; donc voilà. Le truc voilà c’est comme ça quoi, se préparer à...
- Toujours anticiper, tout le temps.
- Voilà, tout le temps, tout le temps, tout le temps, tout le temps.

Vous c’est pareil vous anticipé toutes vos sorties ?

- Ben pff , anticiper pff, oui et non ; enfin moi j’en fait pas trop part parce que bon, chacun a ses problèmes, alors à la limite je souffre en silence. Mais bon des fois moi je vais dehors parce que sinon je m’énerverais pour un oui ou pour un non quoi. Alors que…

Vous êtes plus susceptible ?

- Ouai voilà.

Est-ce que vous êtes soutenu par vos familles pour vous inciter à faire de l’activité physique ou alors êtes-vous surprotéger ?

- Oui c’est un peu ça oui . Et puis…Enfin moi c’est différent c’est depuis l’âge de 11 ans donc c’est vrai que j’ai ma mère…si jamais…d’ ailleurs je lui cache des fois tellement elle est…Je lui cache des trucs parce que.. « ça va aujourd’hui ? » « oui

oui », alors que j’ai pas été au travail. C’est… Parce que sinon, au moindre truc… J’ai marché le matin : « t’en a fait de trop, va doucement, tu vas plus pouvoir marcher, vas-y progressivement et tout ». C’est depuis longtemps donc bon… Ma femme c’est pareil. C’est je me lève pas du canapé, au petit soin et tout. C’est gênant quoi. Tout le monde, tous ceux de ma famille maintenant ils savent que voilà c’est comme ça. Tous mes copains tous, le savent. S’ils font un truc c’est par rapport à moi quoi. Voilà s’ils veulent faire ça, voilà pour M. ça ira. Voilà c’est ça. Mais bon je les ai toujours. Ils vont pas faire un truc sans…voilà c’est ce qui est bien quoi. Je suis bien entouré de ce côté-là. C’est vrai que le moral c’est vrai que. Au travail c’est pareil, tout le monde me comprend et tout. C’est pour ça que je cherche pas à avoir un poste aménagé. Je cherche pas à embêter mon travail pour ça parce que demain si ça va pas, repose toi quoi vas-y. Que je sois derrière un ordinateur ou quoique ce soit, avec cette maladie-là, j’ai mal pareil. Rester sur une chaise toute la journée… Donc c’est…Demander un poste aménagé non je les embête pas avec ça. Donc heu voilà, la médecine du travail ils ont rien vu du tout, j’ai été obligé de leur dire à la fin, ils voyaient rien. Et sinon voilà quoi, au quotidien, au travail ça va. Donc c’est vrai que quand on a tout ça… déjà c’est énorme. Parce que bon, si au travail ça va pas, que vous arrivez pas à suivre, ils peuvent vous…y ’en a c’est ça. Y’a des boites, allez, va voir ailleurs, inapte, et… C’est à double-tranchant. Quand t’es inapte quelque part après…vous êtes…faut aller voir ailleurs, puis y vont vous donner zéro quoi, pensions, quoique ce soit. Voilà quoi.

- Ouai c’est un peu marche ou crève quoi. Moi je vois je suis chauffeur, si je peux pas conduire, j’ai pas de place de secrétaire ou de…les autres postes c’est aussi physique que moi, donc c’est… Ou je me soigne ou sinon bah, je sais pas quoi.

Et votre famille et votre entourage ont quelle attitude ?

- Ben non, j’en parle pas trop. Ma femme elle sait que j’ai mal au dos mais comme moi je sais même pas ce que je vais devenir donc pour l’instant je dis voilà je fais ça je fais ça et puis j’espère que ça ira mieux et puis voilà. Elle elle a ses soucis, chacun à ses soucis donc c’est pas la peine d’en rajouter non plus. Y’a assez de problème dans la vie. J’espère bien, je touche du bois, pour que ça aille mieux, et puis que ça soit derrière moi quoi. On a qu’une vie et puis…
- Moi c’est un peu un mélange des deux. En fait, pour ma mère, c’est un mal de dos, mais voilà je suis jeune donc ça va se soigner. Ma copine par contre elle s’inquiète plus. Elle a plus tendance à m’aider, si ça va pas, à me dire attends reste là je vais le faire. Au travail y’a des personnes qui sont compréhensives, qui comprennent parce que de temps en temps ils ont mal au dos aussi. Donc ils savent ce que… comme eux c’est de temps en temps et que moi c’est tous les jours, donc ils savent que ça va pas des fois. Y’a des personnes au boulot qui disent les jours où je suis en maladie : « alors c’était bien les vacances ? »
- Ouai voilà..
- Ils comprennent pas et puis ça monte à la tête. C’est énervant à force, ça fait péter un plomb.
- Ah c’est sur ça. Le regard des autres ça.

- Pour eux en fait, y’en a y sont meilleur que tout le monde. Y’en a ils ont mal au dos mais c’est…si moi j’ai mal au dos tous les jours, lui il aura mal de temps en temps mais lui les douleurs qu’il aura ça sera plus fort que les miennes. Je sais pas il a pas à juger en fait si ses douleurs sont plus fortes que les miennes. Il est pas capable de le… nan mais c’est pas… Y’en a par contre qui sont compréhensifs. Mon chef d’atelier, il a eu des problèmes aussi. Du coup voilà, avant il me faisait charger les camions et tout, du coup il me dit non t’y va plus. Après quand on va commencer un chantier bah il me dit : « ouai y’a ça à faire est-ce que t’es capable de le faire ? Sinon si t’es pas capable je te donne un autre truc ». Donc il regarde bien avant. Donc ça c’est bien. Y’a des personnes par contre qui vont dire « ah mais regarde le lui il fait un plus petit chantier que le mien, moi je suis tout le temps sur des gros chantiers. » Voilà alors que ça fait des années qu’il est dedans, et puis déjà y’a pas le même salaire, et puis y’a pas les mêmes douleurs, c’est... Moi ça fait pas longtemps que je suis dans la boïte, et du coup en fait bah, c’est comme si c’était de la jalousie un peu.
- Les comédiens, les gens maintenant c’est ça…

C’est ce que vous ressentez aussi ?

- Ah oui oui. Parce que moi avec les rhumatismes bah ça fait…C’est différent j’ai tout le temps mal au dos. Mais le matin si je peux pas me lever, bah je vais pas au travail. Bah j’appelle mon médecin généraliste, elle m’arrête. Et le soir ma femme elle me dit « tu vas chercher la petite à l’école comme t’es arrêté ? » J’lui dit bah j’irais mais laisse-là à la garderie, parce que y’a des collègues ils vont pas comprendre. Parce que le soir, j’arrive, ils vont dire « et bah l’autre et bah ça y est, il est pas si mal que ça je l’ai vu chercher sa fille. ». Le matin je pouvais pas me lever, l’après-midi à force j’ai réussi à me sortir du lit ça va mieux. 16h30 oui je vais la chercher. Et bah j’attends 5h, 5h15, elle reste à la garderie, pour pas avoir le regard de mes collègues qui vont chercher leurs enfants à l’école. La réaction, peut-être qu’ils disent rien. Mais c’est facile de dire après au travail «oui je l’ai vu le grand là, il est pas si malade que ça lui. » Bon je cours pas mais… « oui oui il était pas mal là ». Rien que ça bah…C’est moi qui me fait peut-être des idées mais voilà c’est la mentalité elle est comme ça. J’ai l’impression oui. Puis je suis sûr que c’est ça. C’est ça c’est la mentalité, c’est ce qu’il vient de dire, c’est partout pareil. C’est une entreprise, on est une usine, on est pas loin de 300, voilà on peut pas plaire à tout le monde. Les gens, toujours des jaloux, toujours des… Le regard des gens, ils peuvent pas comprendre… C’est vrai j’ai un travail où je peux venir la journée du matin ou du soir. Et ben des fois le matin je peux pas me lever, pas grave, je me prends même plus la tête, je reste au lit, et je me lèverai, j’irai à 1h, c’est pas grave je finirais à 21h mais… Donc ma femme de ce côté-là s’adapte quoi. Des fois elle a entrainement de hand, ben je dis ouai mais bon ça m’embête t’as entrainement de hand : « non non mais c’est bon, vas-y c’est pas grave j’irais au prochain ». et voilà, à cause de ça ça change toute l’organisation de la famille ; la petite elle a peut-être pas envie de rester à la garderie mais je la laisse quand même à la garderie pour pas que les gens me regarde. Et c’est au quotidien, voilà c’est des choses…Y ’a pire hein, mais la vie bah c’est tout autour de moi quoi,

par rapport à moi. Si je peux pas faire un truc bah c'est pas la peine le week-end il tombe à l'eau. Comme tu dis, ta femme veut aller au ski toi tu peux pas, bah on dit on y va pas et puis… Bon voilà on a l'impression qu'on est un boulet presque pour les autres, et ça c'est…

- Les gens qui sont pas confrontés à ce problème peuvent pas comprendre. Enfin ils comprennent mal quoi. Moi c'est vrai que dans la boite j'ai un ou deux représentants qui se sont fait opérer d'une hernie discale, bon ils ont vu que ça trainait. Et puis les chefs pareil. Mais quelqu'un qui est bien portant il comprend pas qu'on est mal. C'est quoi le mal de dos. Et quand on écoute la télé, la radio, le mal de dos c'est un mal français, on se dit qu'on fait parti des français qu'on mal au dos mais…On se pose des questions, parce que pourquoi maintenant, avant on avait pas mal et puis...
- C'est ce qu'on disait ce matin. Sur une douleur de 1 à 10, je vais me mettre, parce que je souffre tellement, je vais me mettre 4. Y'en a ils auraient 1 dixième de ce qu'on a, ils mettraient peut-être 8 parce que voilà. Et moi je rigole maintenant parce que dans la métallurgie, c'est physique. J'ai des collègues qui étaient en pleine santé et, maintenant c'est juste si c'était pas moi qui était là le plus souvent quoi. Ca rattrape tout le monde. C'est pour ça je te dit toi t'es jeune, mais tu vas voir les autres après ils vont... à un moment ou à un autre ça tombe sur beaucoup de personnes. On arrive à des âges où ça te tombe dessus. Après pour se remettre, le corps il… donc ça arrive à tout le monde donc à un moment ou l'autre tous ceux qui ont rigolés... Tu vas voir si ça se trouve ça peut leur arriver et je vais te dire ce jour-là tu rigoles du coin de... tu rigoles pas devant eux mais....
- Une fois j'étais en arrêt maladie, ça faisait déjà trois jours, puis bah fallait que j'emmène.., ma copine avait pas le permis, donc je l'ai emmené pour faire les courses. Et y'avait un collègue du boulot qui avait posé une journée de congé donc il m'a vu. Et moi du coup bah moi j'ai même pas fait attention à lui, je l'ai pas vu. Lui il m'a vu mais il est pas venu me voir pour me dire bonjour, il est resté dans son coin. Puis le lendemain il est arrivé au boulot, moi j'étais encore en maladie jusqu'à la fin de la semaine « eh j'ai vu F. il était en train de s'amuser, il était en vacances, dans le magasin il s'éclatait, machin et tout. ». Donc pour eux même aller faire les courses…Si j'ai mal au dos faudrait que je reste chez moi, j'ai plus le droit d'aller faire des courses, d'aller chercher du pain…
- Faut pas oublier que les médecins ils mettent sorties libres aussi. Les gens ils oublient ça.
- Voilà pour eux il faudrait que je reste chez moi. Et par exemple si j'ai pas de courses bah faudrait que je reste sans rien. Faut pas bouger de chez soi. A peine ils vont te croiser dans la rue, tout de suite ils vont dire « bah l'autre il était là, il faisait autre chose, c'est qu'il était pas bien malade. »
- C'est la mentalité de maintenant. C'est partout. Les gens sont jaloux du voisins donc…
- L'autre fois j'étais en maladie, ils parlent de j'sais pas quoi des intérimaires qui venaient jamais au boulot, et puis ils disaient « ah moi je vais faire comme F. je vais venir quand j'ai envie ». Moi c'est pas passé, j'ai dit à mon chef bah je rentre chez moi.

- Ça fait mal en plus ça ; tu prends sur toi, au quotidien tu prends sur toi.
- Puis j’ai été voir le chef une fois, pour celui-là, il arrête pas de dire, de se moquer tout le temps... moi je dis à mon chef, moi si ça continue comme ça je démissionne. Je peux pas continuer avec des gens qui ont aucune mentalité. Et pour eux c’est…Ils jugent trop, trop pour rien.
- C’est dur d’évaluer la douleur quand même sur un chiffre. Parce que selon le moment de la journée, si c’est le matin par exemple on va dire ptin on a toute la journée à faire, sachant qu’on travaille, on va dire on souffre admettons à 8. Le soir on va dire bah on va se coucher, la journée est finie alors on va mettre 2, alors que la douleur sera toujours la même quoi ; C’est…Après savoir si depuis le temps que ça traine la douleur elle est peut-être pas à 10 tout le temps mais sur la durée ça fait que c’est énervant, c’est pénible…

Donc pour vous ce qui est le plus pénalisant c’est la douleur sur la durée ?

- Ah bah pour ma part oui c’est malgré les cachets, malgré les séances de kiné, c’est que la douleur soit toujours là, qu’elle soit persistante. Et oui, depuis le temps c’est… Je vois pas d’amélioration c’est ça le problème quoi. Je me dis, il me reste 12 ans à travailler. En gros si je dois me trimballer ma douleur pendant 12 ans ça fait beaucoup quoi. Sachant que ça peut empirer je pense, ça va pas s’arranger. C’est pour ça qu’on est là, qu’on compte beaucoup là-dessus, enfin pour ma part. On veut pas m’opérer, on veut plus me faire d’infiltration, donc après les cachets me font plus effet donc j’ai plu tellement de choix quoi.

Vous avez l’impression d’être seul face à votre problème ?

- Seul non, parce qu’on a vu quand même pas mal de professionnels de santé. On est un peu démuni quoi.
- Ne pas trouver le bout en fait du...L’impression que ça s’arrête pas. Pour ma part ça empire. Et je vois jamais le bout. Je me dis ouai ça va se régler, je vais faire ça ça ça, et puis en fait au bout du truc j’ai encore plus mal ou alors ça c’est empirer. Moi en fait plus le temps passe, et plus mon dos se courbe se courbe en avant. Je sais pas, pour moi ça va pas assez vite en fait. Jsais pas après on va me dire au bout d’un moment, voilà vous êtes trop tordu on peut plus rien faire. Alors que bah peut être que au bout de deux ans avant, et bah peut être ils auraient pu faire quelque chose, s’il n’y avait pas eu autant d’attente.
- Moi c’est... des fois le matin je me lève je suis bien, jsuis content. J’arrive au travail et tout : « ah t’a un torticolis ? ». « bah non pourquoi ? » « bah je sais pas tu peux pas te tourner plus que ça ? » « non non ça va, aujourd’hui je suis bien » « ah, bon bah d’accord. Mais t’es tout tordu redresse toi » « bah je peux pas être mieux ». Pour moi j’étais bien.

- On me dit tiens-toi droit. Moi je suis droit en fait. « Tiens-toi droit ». Pourtant moi je dis moi je suis droit. Des fois au boulot on m’appelle le bossu. Après on prend à la rigolade des fois, mais après à la longue...
- Bah c’est quand t’es bien et puis qu’on te dit « qu’est-ce qui t’arrive »
- Là après la journée elle est foutue.
- Ouai voilà le moral il finit à zéro.
- On a tous les trois mal au dos en fait. On a 3 âges différents et puis en fait on a mal au dos, mais on a pas la même pathologie quoi.
- Par contre on voit que par rapport aux différents mal de dos, y’a des choses qui sont identiques en fait. Au travail…Y ‘a beaucoup de choses qui sont… sur les personnes qui jugent les gens.
- La conjoncture actuelle, t’arrive à des âges où je veux dire demain, tu peux plus le faire, chauffeur routier, comme il dit, l'entreprise y peuvent te dire allez c’est bon au placard. T’as l’impression que par rapport à la société tu sers plus à rien. Alors on a trois âges différents, toi t’as 26, moi j’ai 38. Mais je dis attends y me reste encore du temps, je veux dire je vais faire quoi. Alors les médecins, facile, y’en a qui me disent ouai faut changer de métiers. Vous êtes marrants. J’ai entendu mais alors bon, changer de métier je veux bien, mais après…Mon rhumatologue y m’a dit attention c’est à double tranchant. Faire des demandes pour changer de poste et tout ça ça peut être dans les entreprises ils ressortent les feuilles et voilà moi j’ai pas trouvé de place donc il est inapte, au revoir, payer formation. C’est ce qu’ils sont en train de faire à une personne chez nous. Il était mal vu entre guillemets. Moi je suis peut-être un peu mieux vu que lui mais lui pendant 1 an ils vont l’amuser et après il dégage. Voilà où on en est arrivé. Il va faire quoi ce garçon ? La conjoncture actuelle... On est à Vichy, c’est pas le bassin... On n’est pas riche. Donc ça fait un peu peur quand même. Moi j’ai eu le boulot à Paris demain si je veux, mais si je peux éviter d’aller là-bas ça va m’arranger. Mais je sais que j’en ai, j’ai de la famille là-bas et tout. Mais si je peux rester, ça va pas me faire pleurer. Puis ça va encore tout changer à cause de moi quoi, ma femme... Donc euh, non non c’est pas évident.
- Et puis c’est pas parce qu’on aura un autre travail qu’on aura moins mal au dos aussi.
- Tout à fait.

Et vous vous avez pas fait de demande pour aménager votre poste de travail ?

- Moi ils le savent. Donc en fait ils m’ont mis à un poste… voilà ça va. Je veux dire y’a déjà des trucs que je fais plus. Je dépanne parce que des fois quand la personne est pas là, je sais faire marcher toute les machines. Je dépanne aussi, la boite me paye quand même. Mais voilà, y’a des choses…comme j’vous dis… La médecine du travail m’a juste dit de pas porter des charges lourdes. Je veux faire un truc, je veux dire je fais plus rien dans c’te boite, parce que ils me marquent inapte de partout. Donc euh je dis rien, voilà, mettez moi ça, et puis voilà, ça passe. Mais au travail ils le savent ce que j’ai. Maintenant voilà je suis là pendant 3 semaines, vas-y , fais ce que t’a à faire, ils m’embêtent pas quoi, ils me contrôlent pas quoi, c’est pas…déjà c’est déjà bien…Au mois de décembre c’était moins

marrant. J'me dis là, je fais quoi là. 1 mois et demi à la maison, et puis à me tenir après le bar. J'arrivais pas à me tenir, le dos j'arrivais pas. Là...38 ans…je vais faire quoi là. Mais là ça va mieux là, depuis le 10 janvier c'est vraiment mieux quoi, vraiment bien. Mais voilà ça peut revenir. On a pas eu un gros hiver. Parce que moi je sais le temps que ça va faire, je peux vous dire combien ça va faire demain, alors là c'est sûr, là vous êtes tranquille. Parce que quand le temps y change, j'ai pas besoin d'ouvrir le volet, c'est horrible. Donc voilà, c'est notre quotidien, puis voilà, je pense qu'on est tous pareil.

- C'est la crainte de perdre mon travail. Je pense je vais le garder, parce que ils m'ont dit que ça va bien je bosse bien et tout, mais euh, c'est toujours une crainte parce que, si je suis trop souvent en arrêt maladie ou quoique ce soit, que au bout d'un moment si ils sont obligés de licencier, que je serais peut-être le premier parti, parce que je suis souvent en arrêt maladie et que ça sera peut-être le motif pour eux…Enfin ça sera peut-être, pas le motif, ils vont se dire voilà qu'ils ont plus de boulot, qu'ils vont faire un licenciement économique, mais en fait la vraie raison ça sera que…

- Tu peux demander, je te coupe mais, tu peux demander…moi j'ai…on m'a proposé carrément, travailleur handicapé, eux ça les arrange aussi. Ils sont dans le quota, ils payent pas d'amende. Et toi après bah voilà..
- On m'a proposé aussi de changer de travail et tout. J'ai pas de…Voilà j'ai arrêté l'école en troisième, après j'ai passé un CAP…Enfin j'ai eu plusieurs CAP mais bon j'ai pas un niveau d'étude aussi très élevé. Donc on me demande des fois de faire un travail qui est moins manuel mais bon j'ai pas les diplômes aussi pour le faire. Quoiqu'il arrive j'aurais toujours un travail manuel. Même si je pense à en trouver un autre, quoiqu'il arrive j'aurais toujours un travail manuel. Parce que le travail manuel aussi ça me plait. Voilà c'est…

Vous avez peur de demander des aménagements ?

- J'ai changé 7 fois de métiers, j'ai fait plusieurs métiers, j'ai essayé plusieurs choses : carrosserie, plâtrier-peintre, façadeur, soudeur…Et y'en a aucun qui a réussi à me soulager, enfin comment dire, à moins forcer au niveau du dos. Ça a tout le temps été…Enfin ça a pas été…Enfin on forçait différemment mais enfin la douleur était la même quoi.

Est-ce que certains ont appris en séance de kinésithérapie des auto-exercices à faire à la maison ?

- Oui.

- Moi les seuls exercices que j'ai c'est un DVD qu'on m'a donné en rhumatologie.

Et alors du coup est-ce que vous arrivez à poursuivre ces exercices dans le temps ?

- Oui. Le dernier kiné qui m'a fait faire…Enfin la seule qui m'a fait faire des exercices comme on a fait avec votre kiné, la chaise entre autre, bah c'est pas le plus plaisant mais bon, renforcement abdo, étirement, beaucoup d'étirement.

Et vous arrivez à vous y tenir chez vous ?

- Euh…Oui. Enfin oui jusqu'à venir là. Parce que c'est vrai que des fois c'est tellement pénible d'avoir encore mal. On se dit tiens on va y aller pendant 3 semaines toute la journée. Bon…on verra après... Là ils nous ont bien dit que faudrait qu'on continue, ça sera un travail continuel quoi.

- Moi ils m'ont tout arrêté. Le kiné, ils ont arrêté parce que ça me faisait trop mal. Les exercices chez moi ils m'ont dit d'arrêté parce que ça allait pas non plus… N'importe quel exercice qu'on faisait ça me faisait une douleur vraiment insupportable.

Pensez-vous que si on vous donne un support multimédia cela vous inciterait à faire les exercices au long cours ?

- Oui, ça peut aider pour les faire. Au moins on voit la chose, on essaye de reproduire le même…

Si on vous montre les exercices sans support autre, serez-vous capable de les refaire chez vous au long court ?

- Oui.

- Ah oui.

Et qu'est-ce qui vous motiverait ?

- En se disant qu'on aura peut-être plus mal.

- Qu'on pourra reprendre des activités.

Est-ce que le fait qu'il y ai une structure autour ça vous aiderait ?

-Juste de voir que la douleur pourrait disparaitre et qu'on pourrait refaire des loisirs qu'on pouvait pas faire avant. Juste ça c'est déjà une motivation énorme.

- C'est la motivation, comme pour tout. Si on veut faire un régime faut être motivé, si on veut arrêter de fumer, faut être motivé. Si on veut…J'sais pas moi c'est…

- En fait c’est de pouvoir vivre normalement en fait. Juste ça ça serait déjà énorme.

-Moi je me suis fixé des objectifs.

- A forcer différemment. Ça empêchera que ça empire.

Focus groupe n°4

Durée : 18 min

3 participants + 1 animateur + 1 observateur

Quelle est l’histoire de votre lombalgie ?

- A force de travailler, ça fait deux ans que j’ai arrêté, et ça veut pas passer.
- Moi ça fait pas t’a fait deux ans et j’ai repris au milieu le travail quelques mois et j’ai arrêté à nouveau, j’ai repris au bout de trois, j’ai du reprendre.
- Je pense que c’est… ben… je pense que à l’origine, à l’origine, je dis bien entre guillemets je suis pas médecin quand même, je pense qu’ils auraient pas dû me faire la péridurale, quand notre fils est né, à partir de là… j’ai un pied qui était paralysé on m’a fait une arthrodèse, après les douleurs ont commencées. J’me souviens ptre d’une fois, oui une fois, je m’étais arrêtée, bah le temps passant, le quotidien ayant pris le dessus j’ai laissé courir, j’ai vu le kiné, j’ai été suivie médicalement, c’est pas ce que je veux dire, mais au point de vue douleur ça a commencé… avant, peut- être en 2009.

Pour vous, qu’est-ce que l’activité physique ?

- Se dépenser.
- Transpirer, suer.
- Moi je courrais beaucoup derrière mes chiens, je faisais beaucoup de cheval et j’avais un travail très physique où on transpirait beaucoup.
- Moi j’en ai plus d’activité ça fait plus de 15 ans… j’ai tout arrêté.
- Moi aussi j’ai fait un ptit peu de cheval avant de me marier, j’avais fait un peu de piscine, j’avais essayé, mais comme je suis pas bien courageuse au niveau physique… ah si j’ai fait beaucoup beaucoup de kiné, ah par contre ça…

Avez-vous eu des séances de rééducation ?

- Oui mais massage, et des électrodes.

Ces séances vous ont-elles permis de reprendre un peu d’PA par la suite ?

- Oh moi j’en fais bien un peu, je vais surtout aux champignons marcher dans les bois , c’est tout . C’est déjà pas mal.

Comment s’est passé la dernière fois où vous avez fait de l’activité physique ?

- Ah bah moi je fais le jardin 5 min j’ai mal. Je tronçonne 10min j’ai mal, je porte surement mal les trucs j’ai mal, je vais dans les bois, passer sous les branches au bout d’un moment j’y arrive plus, je cours 200m j’ai mal. Sinon je marche, j’ai pas mal et je continu à faire un ptit peu de cheval, tout seul tranquillement. J’en fais régulièrement. Ce WE j’ai voulu en faire mais au bout d’une heure ça m’a calmé. Sinon j’en fais régulièrement quand même.
- Au début j’essayais bien de bricoler mais ça dépend, des jours ça va faire mal et d’autre non. Au repos des fois ça fait mal, d’autre fois pas, et en bricolant des fois ça fait mal d’autre fois pas, ça dépend des postures jpense. Ca dépend, c’est des crise à mon avis.
- J’en ai pas fait depuis 20 ans. Le cheval depuis que je suis marié je suis pas remonté dessus. 22 ans presque. Oui 22 ans qu’on est marié, puis bon notre fils, comme je me suis marié un peu tard, bah il est né , on a voulu un enfant tout de suite, ça a marché tout de suite. Quand j’étais enceinte il a fallu que je reste allongée, au bout du 6ème mois le petit venait au monde, le col s’ouvrait le petit venait au monde. Je crois que c’est 6 ou 7ème mois . Non mais vous savez je faisais les trajets Vichy/Moulins par contre je ne travaillais qu’à 80%. Autrement j’ai rien fait. Vous savez le travail de maman c’est tout, voilà ça s’arrêtait là ! (rires). Et non j’ai rien fait.

Qu’est-ce qui vous freine à faire de l’activité physique ?

- Le temps, on prend pas le temps ptre.
- Moi le temps je l’ai, ah moi je l’ai.
- Moi peut-être un manque de volonté, certainement.
- Moi que la douleur, uniquement, j’ai le temps, j’ai la volonté.
- Et puis maintenant c’est la douleur, mais y’a quelques années c’était la volonté. J’avais laissé tomber le kiné. On a tout laissé tomber pendant quelques années, on a dit ça va bien allez hop hop hop c’est bon. Donc là ouai bah là maintenant j’ai vraiment…Puis j’ai peur, maintenant j’ai peur. Par exemple de remonter à cheval, on aurait possibilité...Mon mari a des clients qui ont des chevaux, ils m’en prêteraient bien un qui soit docile mais j’aurais peur de tomber. Il y a les deux enfants, faut quand même penser à eux. C’est vrai que des fois comme vous me disiez il existait des étrillés, parce que mon pied est bloqué presque à 45° avec l’arthrodèse, il existe des étrillés vous dites plats ?
- Ah oui t’a le pied qui est complètement sur un étrillés, sur une planche qui est tenue, donc t’es bien à plat.
- Donc y’a pas ce mouvement..
- Non y’a pas le mouvement de la cheville.

Qu’est-ce qui a changé dans votre quotidien ?

- Moins de courage.
- Oui moins de courage.
- Bah je travaille plus déjà.
- Moi non plus je travaille plus.
- Oui moins de courage. Après ça va repartir.

Quelle est l’attitude de votre entourage ?

- Moi il me freine.
- Oui oui moi aussi il me freine.
- Sinon je me fais engueuler.
- Oui moi aussi je me fais engueuler, ils m’ont dit « tu veux finir dans un fauteuil roulant ». « Continuer comme ça sur cette lancée et vous finirez dans un fauteuil roulant ; Là ça sera finit vous courerez plus »
- Moi on m’engueule mais je fais.
- Ouai moi je m’en fous.

Qu’est-ce qui vous motiverait à pratiquer plus d’activité physique ?

- Bah si j’avais pas mal.

Le fait de pratiquer à plusieurs vous aiderait-il ?

- Non non avant je partais à cheval tout seul pendant des heures ça ne me gênait pas du tout.
- Qu’on soit plusieurs oui.
- Ah moi je m’en fous complètement.

Qu’est-ce qui vous aiderait à maintenir les exercices dans le temps ?

- Qu’on me mette des tartes si je les fais pas.
- Oui peut-être oui peut-être, t’as ptre raison.

Donc un suivi ?

- J’suis sûr que ça aiderait oui.
- Moi aussi toute seule je fais rien. Si y’avait pas le kiné toute les semaines…
- Moi j’en ferais bien mais c’est vrai qu’au bout d’un moment les exercices tu te fais chier, c’est pas des plus amusant.

La présence d’infrastructures à proximité pour faire de l’activité physique vous aiderait-elle ?

- Si j’allais mieux en faisant des exercices je pense que ça tiendrait.

Y a-t-il des activités que vous faites moins qu’auparavant ?

- Ah bah moi je fais plus de jardin, j’ai du terrain je m’en occupe plus, j’avais un grand verger que j’ai laissé tomber. Je m’occupe moins bien des chevaux. Oui bah oui j’en

fais beaucoup beaucoup moins. Bah tâches ménagères j’en faisais pas beaucoup donc euh (rires). Non mais en extérieur énormément moins oui.

- Moi j’essaye… par exemple je sais pas les courses, je m’étais un petit peu accroché avec Vichy parce que je leur ai dit qu’on était plus au moyen-Age même en pleine cambrousse (rires). On me disait : « comment vous faites pour vivre, la vie quotidienne ? », bah je dis les courses c’est tellement facile avec internet un coup de clic, DRIVE, et puis les courses ils les mettent dans votre voiture. Moi je dis après moi j’ai mon mari et mes 2 fils ils sont adorables. Jamais ils me laisseront trainer des packs d’eau des choses lourdes du quotidien ; Les sacs tout ça c’est eux qui s’en occupent… Tout ce qui est ménage on a réduit au maximum pour notre plus grand plaisir avec mon mari. On a réduit au maximum, parce que on a quelqu’un mais pour qu’elle vienne pas trop souvent. Puis voilà.

Pratiquiez-vous une activité physique avant le début de vos lombalgiques ?

- Ah non le sport je l’ai arrêté quand j’étais au lycée. J’ai tout arrêté.

Qu’est-ce qui pourrait vous donner envie de reprendre une AP ?

- Bah c’est qu’après si je reprends le sport et tout c’est qu’après si je me fais mal je peux plus aller au boulot. Après c’est une contrainte pour aller au boulot quoi.

Et si quelqu’un vous encadrait et vous montrait les bons gestes ?

- Mais faut aimer ce qu’il y a à faire. Parce que moi c’était que du sport physique et maintenant… plus le courage de courir et tout. A 35 ans aller courir et tout…
- Tu verras quand t’as 50 ça revient ! (rires). D’ailleurs pour cet été j’organise une randonnée à cheval. On doit partir 5 jours pour faire 220 km. Mais j’ai prévu que si jamais ça va pas, au lieu d’en faire 50 dans la journée je peux en faire que 8. L’an dernier on est pas parti, et l’année d’avant on était parti. On le fait tous les ans. Donc j’espère pouvoir le faire encore cette année.

Vous faisiez beaucoup de sport avant ?

- Non non le cheval c’était vraiment une détente, j’étais pas très physique je préférais un livre. Maintenant.. pfffff

Qu’est-ce qui vous inciterait à vous y remettre ?

- Je sais pas…. Là toute seule… Mon mari ne sait pas monter à cheval puis ça l’intéresse pas, mes enfants non plus.

Le fait d’être en groupe ?

- Ben oui éventuellement. Voyez comme vous , faire un essai, je serais en confiance. Mais là...A mon âge c’est bon.. c’est pas bien grave (rires…)

Un support multimédia vous aiderait-il à faire de l’AP ?

- Je sais pas…faudrait essayer..
- Y’a bien la WII, mais la Wii moi j’y touche pas (rires), y’a ben des jeux et tout à faire dessus mais…
- C’est vachement différent de faire des exercices pour maintenir sa forme comme on fait ici par exemple quand on est tout seul que d’aller faire un tour de cheval ou d’aller marcher ou d’aller faire du sport ou se dégourdir les jambes, ça j’ai pas besoin de…. Ca va tout seul quoi. Mais c’est vrai que faire des exercices pour entretenir sa forme c’est sûr que tout seul c’est compliqué.
- Marcher et courir tout seul non, mais marcher dans les bois oui ça me dérange pas. Mais autrement courir tout seul pfff…

Il y a une notion de plaisir alors ?

- Ah oui oui.
- Il y aurait des champignons toute l’année j’irais toute l’année marcher. (rires)
