# Supplementary material for: Facilitators and barriers to physical activity in people with chronic low back pain: A qualitative study
Source: PLoS One. 2017 Jul 25;12(7):e0179826. doi: 10.1371/journal.pone.0179826 (PMC5526504; doi:10.1371/journal.pone.0179826)
Supplement: S4 File — (PDF) [file pone.0179826.s004.pdf]

ANNEXE 3

FORMULAIRE D'INFORMATION

Evaluation de la pratique de l'activité physique chez les patients lombalgiques chroniques

- **Promoteur**
  C.H.U. de Clermont-Ferrand
  58 Rue Montalembert
  63003 Clermont-Ferrand Cedex 1

- **Investigateur principal** :
  Pr Emmanuel Coudeyre, Service de Médecine Physique et de Réadaptation (MPR),
  CHU Clermont-Ferrand / Hôpital Nord, BP 30056, 63118 Cébazat
  Emilie Richard, Interne en médecine générale (IMG) affiliée à la faculté de Clermont Ferrand.

Etude :

Etude prospective, qualitative auprès d'une cohorte d'une vingtaine de patients souffrant de lombalgies chroniques évoluant depuis plus de 3 mois, dans le but d'évaluer, à partir des données initiales de la littérature et d'entretiens individuels, les habitudes à la pratique de l' activité physique.

Madame, Monsieur,

Votre médecin généraliste ou MPR vous propose de participer à une étude dans le cadre d'un travail de thèse, au sein de la région auvergne et du CHU de Clermont-Ferrand.

Cette étude a pour objet d'étudier la pratique de l'activité physique chez les patients lombalgiques chroniques. Il est important que vous ayez bien compris le principe et le déroulement de cette étude, avant d'accepter d'y participer. Nous vous remercions de bien vouloir prendre le temps nécessaire à la lecture détaillée de ce document. Il est possible que ce formulaire contienne des termes difficiles à comprendre. Votre médecin répondra volontiers à toutes les questions que vous jugerez utiles de lui poser sur le contenu de ce document.

1. *Objectifs de l'étude*

L'objectif principal de cette étude est d'obtenir un maximum d'information sur le vécu de votre lombalgie, ainsi que sur votre pratique de l'activité physique. Ces données seront recueillies lors d'un entretien individuel unique.

2. *Déroulement de l'étude*

Cette étude se compose d'un entretien individuel soit au cabinet de votre médecin généraliste, à votre domicile, ou bien au CHU de Clermont Ferrand dans le service de MPR.

Il se déroule dans un premier temps par un interrogatoire médical comprenant des données générales (âge, profession, loisir...) et médicales sur votre lombalgie et vos antécédents. Dans un second temps que l'on appelle entretien semi dirigé vous vous livrerez sur le vécu et le ressenti de votre lombalgie. Il n'y a pas de durée définie pour l'entretien.

Vous pouvez bien sûr arrêter, à votre convenance l'étude.

3. *Respect de l'anonymat*

Il est nécessaire de recueillir des données sur des fiches, et des données informatiques vous concernant. Chacun des patients correspond à un numéro d'identification par ordre chronologique d'entretien. Votre nom n'apparaît à aucun moment de l'étude afin de garantir votre anonymat, selon la procédure habituelle dans ce type d'étude. Seuls les noms et identités de quelques patients seront conservés sur leurs fiches (non informatisée) afin que l'on puisse leur faire lire les résultats de l'étude. Seulement après obtention de leur consentement oral. Ceci a pour but d'augmenter la validité de l'étude.

Le projet de l'étude est en cours d'acceptation auprès de la Commission nationale de l'informatique et des libertés qui veille à protéger la vie privée et la liberté dans le monde numérique et donc à respecter l'anonymat.

Les données de l'étude pourront également être utilisées dans des publications scientifiques. Dans tous les cas, votre anonymat sera préservé.

4. *Droit des patients et conditions de participation à l'étude*

Votre participation à cette étude est entièrement volontaire ; vous êtes libre d'accepter ou de refuser d'y prendre part. Si vous décidez de ne pas participer à l'étude, votre traitement et l'attitude de votre médecin à votre égard ne seront pas affectés.

Si vous acceptez de participer, vous pourrez néanmoins interrompre votre contribution à l'étude à tout moment sans que cela ait une influence sur la qualité des soins qui vous sont apportés. Cependant, nous vous demandons d'avertir de cette décision le plus rapidement possible.

Selon les dispositions de la loi « Informatique et Libertés », vous disposez d'un droit d'accès et de rectification des données informatiques vous concernant, recueillies par l'intermédiaire de votre médecin. La présentation du résultat du traitement des données ne permettra en aucun cas votre identification directe ou indirecte. En accord avec la loi du 4 mars 2002 relative aux droits des malades, vous serez informé(e), si vous le souhaitez, des résultats globaux de l'étude par l'intermédiaire de votre médecin généraliste ou MPR, lorsque les résultats seront disponibles.

Votre participation à cette étude n'engendrera pour vous aucun frais supplémentaire par rapport à ceux que vous auriez dans le suivi habituel. Toutefois, pour pouvoir participer à cette recherche vous devez être affilié(e) ou bénéficier d'un régime de sécurité sociale.

Le CHU de Clermont-Ferrand, qui organise cette étude en qualité de promoteur, a contracté une assurance conformément aux dispositions législatives, garantissant sa responsabilité civile et celle de tout intervenant auprès de la Société Hospitalière d'Assurances Mutuelles (SHAM, contrat n°126-016).

Cette recherche a reçu l'avis favorable du Comité d'éthique (Clermont-Ferrand) le 16 Janvier 2012. Il est possible que cette recherche soit interrompue, si les circonstances le nécessitent, par le promoteur ou à la demande de l'autorité de santé.

Pour toute information complémentaire au cours de l'étude, vous pourrez contacter un des deux investigateurs principaux de l'étude.

FORMULAIRE DE NON OPPOSITION A UNE ETUDE MEDICALE

Évaluation de la pratique de l'activité physique chez les patients lombalgiques chroniques

Investigateurs principaux :
Pr Emmanuel COUDEYRE, Emilie RICHARD (IMG)
Service de Médecine Physique et de Réadaptation, CHU Clermont-Ferrand / Hôpital Nord, BP 30056, 63118 Cébazat

FORMULAIRE DE NON OPPOSITION, PATIENT – INVESTIGATEUR

Je soussigné(e)……………………………. autorise par la présente RICHARD E. (Interne en médecine générale (IMG)) en sa qualité d'investigateur dans le cadre de l'étude, à m'inclure dans cette étude.

RICHARD E. (IMG) m'a clairement indiqué que c'est une étude prospective qualitative, non interventionnelle qui vise à étudier la pratique de l'activité physique des personnes lombalgiques chroniques et qui a reçu un avis favorable du Comité d'éthique (Clermont Ferrand) en date du ……….

RICHARD E. (IMG) m'a précisé qu'il s'agissait d'un entretien individuel unique.

J'ai bien noté que mes coordonnées ne seront pas divulguées ni au Promoteur ni à tout autre instance administrative. Mon dossier restera entièrement confidentiel. Cependant, certains détails médicaux anonymes seront examinés par les représentants agréés du Promoteur. En signant, ce formulaire, j'autorise les représentants visés ci-dessus à accéder au jeu de données anonymes afin de faciliter le bon déroulement de cette étude.

Selon les dispositions de la loi « Informatique et Libertés », je disposerai d'un droit d'accès et de rectification des données informatiques me concernant. Je pourrai m'opposer au traitement informatisé des données me concernant. La présentation du résultat du traitement des données ne permettra en aucun cas mon identification directe ou indirecte.

Si pour une raison quelconque, je devais décider de me retirer de l'étude, je serais en mesure de faire parvenir ma décision à RICHARD E. (IMG) et toute saisie de données vers la base de données prendrait aussitôt fin. Cette décision ne modifierait en aucune manière la prise en charge et le traitement de mes pathologies.
